# Inhibiting the biogenesis of myeloid-derived suppressor cells enhances immunotherapy efficacy against mammary tumor progression

**DOI:** 10.1172/JCI158661

**Published:** 2022-12-01

**Authors:** Sean H. Colligan, Andrea M. Amitrano, Robert A. Zollo, Jennifer Peresie, Elliot D. Kramer, Brian Morreale, Joseph Barbi, Prashant K. Singh, Han Yu, Jianmin Wang, Mateusz Opyrchal, David B. Sykes, Michael J. Nemeth, Scott I. Abrams

**Affiliations:** 1 Department of Immunology,; 2 Department of Thoracic Surgery,; 3 Department of Cancer Genetics and Genomics, and; 4 Department of Biostatistics and Bioinformatics, Roswell Park Comprehensive Cancer Center, Buffalo, New York, USA.; 5 Department of Medicine, Indiana University, Indianapolis, Indiana, USA.; 6 Center for Regenerative Medicine, Massachusetts General Hospital, Boston, Massachusetts, USA.; 7 Harvard Stem Cell Institute, Cambridge, Massachusetts, USA.; 8 Department of Stem Cell and Regenerative Biology, Harvard University, Cambridge, Massachusetts, USA.

**Keywords:** Immunology, Cancer immunotherapy

## Abstract

While immune checkpoint inhibitors (ICIs) have transformed the therapeutic landscape in oncology, they are effective in select subsets of patients. Efficacy may be limited by tumor-driven immune suppression, of which 1 key mechanism is the development of myeloid-derived suppressor cells (MDSCs). A fundamental gap in MDSC therapeutics is the lack of approaches that target MDSC biogenesis. We hypothesized that targeting MDSC biogenesis would mitigate MDSC burden and bolster tumor responses to ICIs. We tested a class of agents, dihydroorotate dehydrogenase (DHODH) inhibitors, that have been previously shown to restore the terminal differentiation of leukemic myeloid progenitors. DHODH inhibitors have demonstrated preclinical safety and are under clinical study for hematologic malignancies. Using mouse models of mammary cancer that elicit robust MDSC responses, we demonstrated that the DHODH inhibitor brequinar (a) suppressed MDSC production from early-stage myeloid progenitors, which was accompanied by enhanced myeloid maturation; (b) augmented the antitumor and antimetastatic activities of programmed cell death 1–based (PD-1–based) ICI therapy in ICI-resistant mammary cancer models; and (c) acted in concert with PD-1 blockade through modulation of MDSC and CD8^+^ T cell responses. Moreover, brequinar facilitated myeloid maturation and inhibited immune-suppressive features in human bone marrow culture systems. These findings advance the concept of MDSC differentiation therapy in immuno-oncology.

## Introduction

The ability to harness the immune system through immunotherapy, namely immune checkpoint inhibitors (ICIs), has improved survival for patients with advanced cancers of diverse types, including renal cancer, non–small cell lung cancer, gastric cancer, head-and-neck cancer, breast cancer, and melanoma (1–3). However, except for melanoma, the responses of most solid tumors to ICIs are seen only in smaller subsets of patients, and the overall response rates remain low across those cancer types (2, 4, 5). Immune suppression within the tumor microenvironment (TME) has been demonstrated as an integral barrier to the efficacy of immunotherapy, impairing the ability of tumor-specific CD4^+^ or CD8^+^ T cells to respond with maximal activation or effector function (5–7). A major tumor-driven mechanism of immune suppression is the generation of lymphoid or myeloid cell populations, such as Tregs, tumor-associated macrophages (TAMs), or myeloid-derived suppressor cells (MDSCs), which impede antitumor T cell activity within the TME (8–10).

MDSCs have received considerable attention as a dominant cellular constituent of an immune-suppressive network (2, 3). MDSCs comprise heterogenous populations of immature myeloid cells reflecting various stages of differentiation and are defined by their ability to inhibit T cell activation, proliferation, and effector function (2, 3, 11–13). They are produced in multiple human and mouse cancers, negatively correlate with survival outcomes, and compromise responses to diverse treatment modalities, including chemotherapy, radiotherapy, and immunotherapy (2, 3, 9, 14). In preclinical models and in patients with cancer, 2 prevalent MDSC subsets have been defined: monocytic MDSCs (M-MDSCs) and granulocytic or polymorphonuclear MDSCs (PMN-MDSCs) (15–17). Targeting MDSCs in both preclinical and clinical settings can result in improved outcomes (2, 4, 9). Most current strategies aimed at eliminating MDSCs or inactivating their effector functions, such as the use of chemotherapies, kinase inhibitors, or neutralizing antibodies, are thought to target circulating MDSCs. However, therapies that attack MDSCs in the periphery or the TME are likely to exert a transient effect, as they do not target MDSC production or “biogenesis.” MDSCs can be replenished because chronic exposure of progenitors in the bone marrow (BM) to the secreted and circulating tumor-derived factors (TDFs) can sustain MDSC biogenesis (2, 3, 18). Thus, targeting the source of MDSCs could have a more sustainable benefit, analogous to the concept of targeting cancer stem cells (CSCs) to hamper the generation of neoplastic daughter cells.

We and others have demonstrated that MDSCs emerge from hematopoietic progenitors in the BM, particularly early myeloid progenitors such as granulocyte-monocyte progenitors (GMPs) (18–22). We hypothesized that targeting the BM source of MDSCs would rescue normal myeloid differentiation, reduce the peripheral MDSC burden, and enhance immunotherapy responses. To test this hypothesis, we turned to the concept of differentiation therapy, commonly discussed in the context of acute myeloid leukemia (AML). Although there are significant differences between AML and MDSC biogenesis, a common feature is the central role of the GMP population (21–25). In AML, leukemic GMPs are often the reservoir of leukemia-initiating cells, whereas in the context of solid tumors, GMPs undergo an aberrant differentiation program in response to TDFs, acting as the cells of origin of MDSCs. Thus, myeloid differentiation therapies that target GMPs in AML to mature the blasts into terminally differentiated myeloid cells may be applicable against other myeloid pathologies arising from the same parental source.

Several groups have demonstrated that inhibiting the enzyme dihydroorotate dehydrogenase (DHODH), a rate-limiting step in pyrimidine biosynthesis, unexpectedly and profoundly enforces the terminal differentiation of AML cells, mainly toward neutrophils (26–28). DHODH inhibitors also induced the differentiation of immortalized GMPs, which represent the same cellular origin of MDSCs. On the basis of the rationale that MDSCs, like the development of AML, arise from defects in myeloid differentiation at similar progenitor stages, we reasoned that DHODH inhibitors that target MDSC biogenesis at the GMP stage would enhance myeloid cell maturation, thereby reducing MDSC burden.

One such DHODH inhibitor is brequinar (BRQ). A study by Sykes et al. demonstrated that BRQ, an orally available DHODH inhibitor, had potent in vitro and in vivo anti-AML activity (26). In that study (26), inhibition of DHODH by BRQ induced the differentiation of immortalized GMPs into mature myeloid populations, namely neutrophils. In fact, years ago, BRQ was tested as an antineoplastic agent against multiple solid tumor types, and although the treatment was generally well tolerated, clinical efficacy was not observed across the various clinical trials (29–31). While BRQ treatment as a direct anticancer agent was not effective, we reasoned that BRQ may be effective in combinations, acting as an anti-MDSC therapeutic that could boost immunotherapy efficacy. In this study, we tested this notion by repurposing BRQ in preclinical models of mammary cancer, a disease characterized by high MDSC burden, especially the granulocytic or PMN-MDSC subset (32, 33). Indeed, PMN-MDSCs are the dominant subset in most cancer models (34, 35). Here, we report that BRQ bolstered ICI efficacy in ICI-resistant models of triple-negative breast cancer (TNBC) by affecting MDSC biogenesis. Our strategy, stemming from the concept of disordered myelopoiesis, may have important implications for the treatment of patients with cancer through the use of ICIs or other active or passive immunotherapies in which MDSCs impede therapeutic efficacy.

## Results

### BRQ inhibits the immune-suppressive activity of MDSCs.

To test our hypothesis that targeting MDSC biogenesis would reduce MDSC frequency or suppressive function, we first used an in vitro model of MDSC production. Our rationale for selecting BRQ as a potential MDSC differentiation agent was first supported by gene expression data that revealed significant upregulation of the pyrimidine (as well as purine) synthesis pathway in GMPs from tumor-bearing mice compared with GMPs from non-tumor-bearing (NTB) controls (Supplemental Figure 1A; supplemental material available online with this article; https://doi.org/10.1172/JCI158661DS1). Since pyrimidine synthesis is the specific target of BRQ (36), these data suggested that tumor-induced GMPs may be selectively receptive to BRQ-induced myeloid cell differentiation.

**Figure 1:**
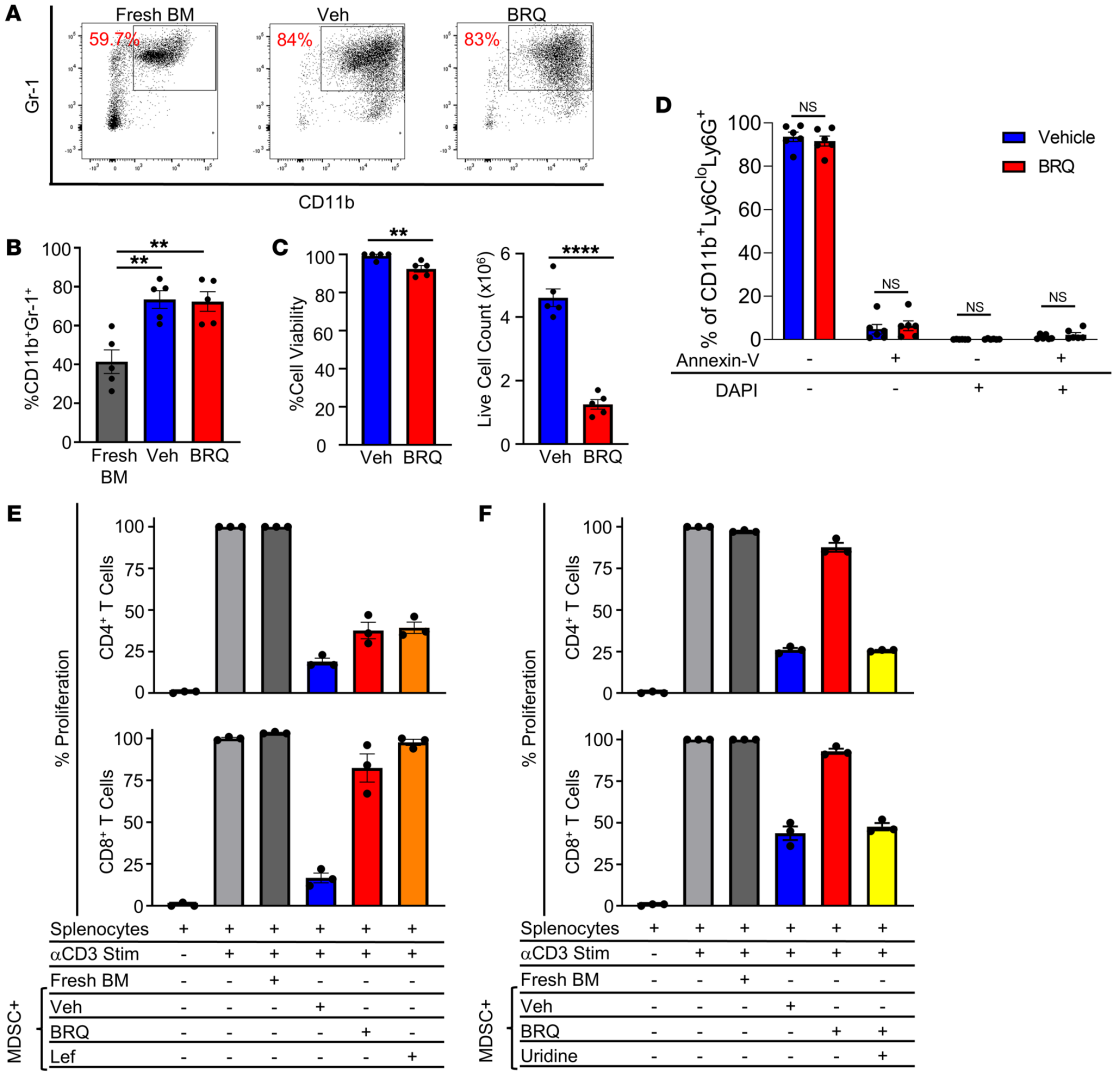
BRQ reverses the suppressive activity of MDSCs. Female BALB/c mouse BM cells were cultured with 40 ng/mL recombinant mouse (rm) G-CSF plus rmGM-CSF for 96 hours with or without 1 μM BRQ (Tocris). (**A**) Flow cytometric analysis of CD11b and Gr-1 expression in cultures treated with vehicle (Veh) or BRQ. (**B**) Percentage of CD11b^+^Gr-1^+^ cells. (**C**) Percentage of viable cells as determined by trypan blue staining and live cell quantification. (**D**) Percentage of apoptotic cells, as determined by annexin V and DAPI staining of vehicle- or BRQ-treated MDSCs. (**E**) CD4^+^ and CD8^+^ T cell proliferation following coculture with MDSCs generated with or without 1 μM BRQ (from Clear Creek) or 25 μM Lef. Splenocytes from naive syngeneic mice were used as a source of T cells and were stimulated with 1 μg/mL anti-CD3 (αCD3) mAb for 72 hours. Cell proliferation was measured using CellTrace Violet. (**F**) CD4^+^ and CD8^+^ T cell proliferation following coculture with MDSCs with or without BRQ in the absence or presence of 200 �M uridine. Data are presented as the mean ± SEM of 5 separate experiments (**B** and **C**), 6 separate mice (**D**), or triplicate determination (**E** and **F**). **P < 0.01 and ****P < 0.0001, by unpaired *t* test (**B**–**D**).

BM cells cultured with GM-CSF/G-CSF showed a significantly increased frequency of CD11b^+^Gr-1^+^ cells, a canonical phenotype of murine MDSCs, relative to freshly isolated BM cells (Figure 1, A and B, and Supplemental Figure 1B). BRQ treatment did not significantly inhibit the production of CD11b^+^Gr-1^+^ cells relative to the vehicle control (Figure 1B). Although cell viability was mildly reduced by BRQ treatment, viability still exceeded 92%. Interestingly, we observed that the number of viable cells recovered was markedly reduced, consistent with an antiproliferative effect (Figure 1C). To further explore a potential direct effect of BRQ on cell viability, we performed apoptosis assays using annexin V and DAPI staining (Figure 1D). As with trypan blue dye exclusion, 90% or more of the cells exposed to BRQ were viable, with 10% or less of the cells collectively showing evidence of either early- or late-stage cell death, and there were no significant differences between the vehicle- and BRQ-treated cultures at any of these stages. We then examined whether these myeloid cells exhibited MDSC activity on the basis of their ability to inhibit anti-CD3 mAb-induced T cell proliferation. Myeloid cells from the vehicle-treated controls inhibited CD4^+^ and CD8^+^ T cell proliferation compared with T cells cultured without myeloid cells or with fresh BM cells (Figure 1E and Supplemental Figure 1, C and D), indicating the generation of MDSCs. In contrast, BRQ reduced the ability of these MDSCs to inhibit CD4^+^ and CD8^+^ T cell proliferation compared with the controls (Figure 1E and Supplemental Figure 1D). We also tested leflunomide (Lef), another small-molecule DHODH inhibitor (37, 38), and found that Lef similarly reduced MDSC suppressive activity (Figure 1E).

Cells can negate the effects of DHODH inhibition by maintaining normal levels of pyrimidine biosynthesis through a uridine salvage pathway, assuming there is sufficient extracellular uridine (39). To confirm that BRQ reduced the ability of MDSCs to inhibit T cell proliferation specifically through DHODH inhibition, we supplemented the culture system with excess uridine, which bypasses the de novo pathway of pyrimidine biosynthesis that requires DHODH activity (40). The loss of MDSC-suppressive activity caused by BRQ treatment was reversed by extracellular uridine supplementation (Figure 1F and Supplemental Figure 1D), demonstrating that the effects of BRQ were both on target and DHODH specific. Together, these data indicated that targeting DHODH could inhibit MDSC-suppressive activity without inducing significant cell death, suggesting a mechanism of differentiation.

### BRQ inhibits the expression of genes associated with the MDSC-suppressive phenotypes and induces myeloid maturation.

**Figure 2:**
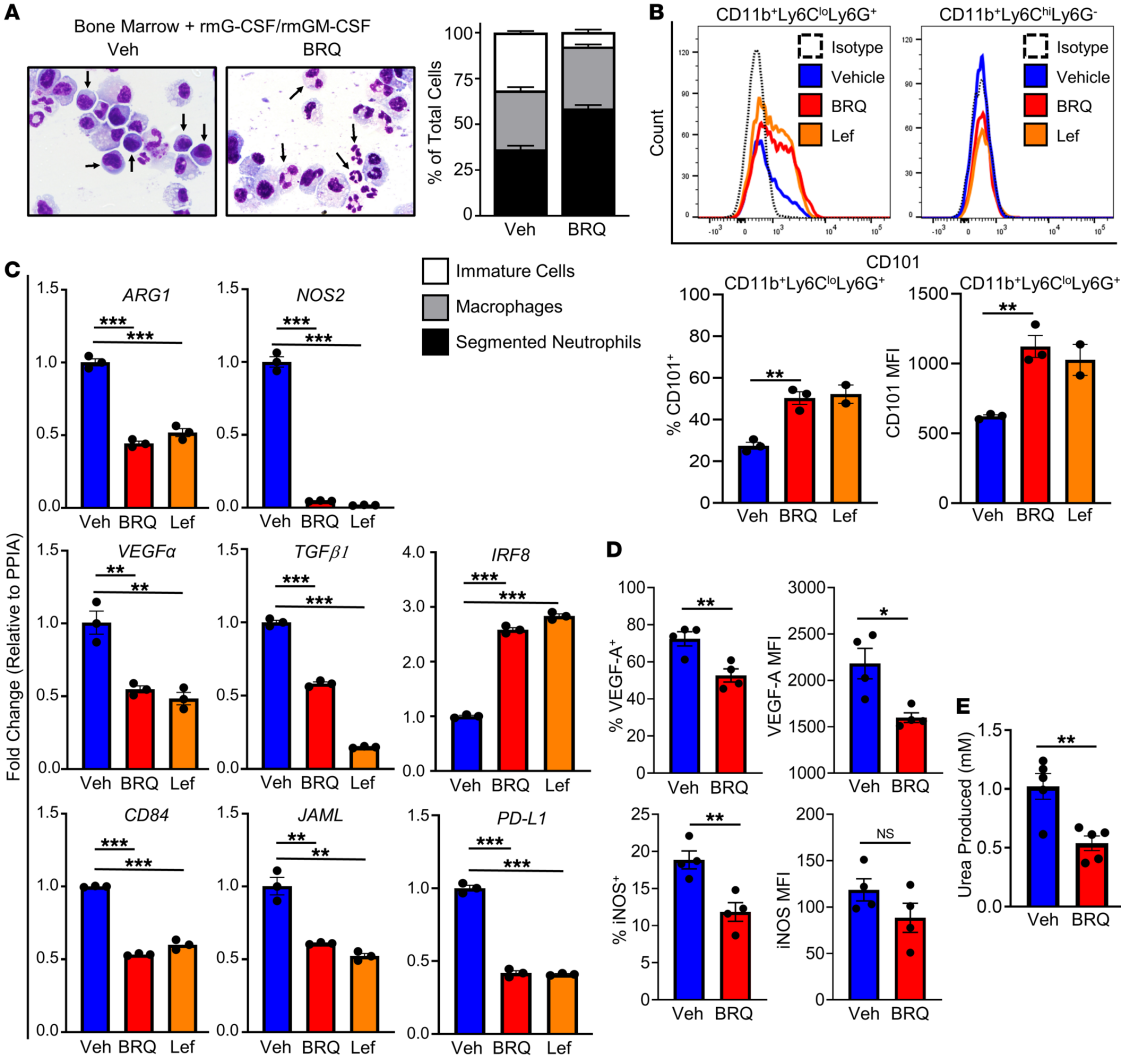
BRQ induces myeloid cell maturation and reduces the expression of immune-suppressive genes in MDSCs. (**A**) Cytospins of BM cultures were stained using Wright-Giemsa and analyzed for the indicated cell populations. Photomicrograph images show cells treated with vehicle or BRQ (from Tocris). Original magnification, ×1,000. The percentage of each cell type, shown in the graph, was quantified as follows: 300 cells/slide for each treatment condition were analyzed (100 cells/field × 3 fields) in biological duplicates. The average number of cells across those 6 fields covering the 2 separate slides possessing the indicated morphology was then recorded as a percentage of the total population reflecting those 3 scored cell types. (**B**–**E**) BM cells were cultured as in Figure 1 with or without BRQ (Clear Creek) or with 25 μM Lef. (**B**) CD11b^+^Ly6C^lo^Ly6G^+^ and CD11b^+^Ly6C^hi^Ly6G^–^ cells were analyzed by flow cytometry for surface CD101 expression. Top: Histograms depict CD101 expression. Bottom: Percentage of CD101^+^ cells (left) and CD101 MFI of the indicated cell subset. (**C**) PMN-MDSCs were recovered after in vitro culturing by a positive magnetic bead selection method (Miltenyi) and analyzed by RT-qPCR for expression of the indicated genes. (**D**) PMN-MDSCs were recovered after in vitro culturing and analyzed by flow cytometry for VEGF-A and iNOS expression. (**E**) Bulk MDSCs were lysed after in vitro culturing for an arginase activity assay, as measured by urea production. Data in **B** are presented as the mean ± SEM or SD of 3 (BRQ) or 2 (Lef) separate experiments, respectively. Data in **C** are presented as the mean ± SEM of triplicate determinations and are representative of 2 independent experiments with similar results. Data in **D** and **E** are presented as the mean ± SEM of results involving 4–5 separate mice. *P < 0.05, **P < 0.01, and ***P < 0.001, by unpaired *t* test (**B**–**E**).

Having shown that BRQ rendered in vitro–derived MDSCs less suppressive (Figure 1), we investigated whether this effect correlated with myeloid cell maturation and/or a reduction in genes associated with their immune-suppressive phenotype. On morphologic evaluation, MDSCs generated in vitro with or without BRQ showed that BRQ treatment increased the percentage of segmented neutrophils, compared with the vehicle control, inferring maturation (Figure 2A). The increase in segmented neutrophils was at the expense of immature cell types, without an appreciable effect on the proportion of macrophages (Figure 2A). Given the finding that BRQ treatment increased the frequency of neutrophils, we quantified changes in CD101 expression on the PMN-MDSC (CD11b^+^Ly6C^lo^Ly6G^+^) subset (Supplemental Figure 2A). Mature neutrophils express cell-surface CD101, whereas immature CD101^–^ neutrophils are associated with tumor progression (41). Consistent with our morphologic findings, we observed significant increases in CD101 expression on PMN-MDSCs in BRQ-treated samples compared with the vehicle control cells (Figure 2B, upper left and lower panels). We observed similar increases in CD101 expression following Lef treatment. CD101 expression was not detectable within the monocytic subset (CD11b^+^Ly6C^hi^Ly6G^–^), with or without BRQ or Lef treatment (Figure 2B, upper panel).

We then analyzed the PMN-MDSC subset for BRQ-induced alterations in the expression of genes associated with immune-suppressive or protumorigenic activity, including arginase 1 (*ARG1*), *NOS2*, *PDL1*, *TGFB1*, and *VEGFA* (42, 43). The expression of CD84 and junctional adhesion molecule (JAML) has been shown not only to further identify MDSCs, but also directly and functionally correlates with MDSC-suppressive capacity (44). BRQ treatment significantly reduced the expression of *ARG1*, *NOS2*, *VEGFA*, *TGFB1*, *PDL1*, *CD84*, and *JAML* (Figure 2C). We previously demonstrated that the myeloid transcription factor IRF8 is a critical negative regulator of MDSC development, particularly of the PMN-MDSC subset (14, 18). Here, we show that BRQ treatment significantly increased *IRF8* expression (Figure 2C**)**, consistent with the ability of BRQ to dampen the generation of MDSCs or their activities. We observed similar gene expression changes following Lef treatment. To extend these observations to a protein level, we focused on 3 of these effector mechanisms, VEGF-A and iNOS expression, by intracellular flow cytometry (i.e., percentage of positive and/or MFI values) (Figure 2D and Supplemental Figure 2B), and Arg1 by an enzymatic assay (Figure 2E). As with the mRNA analysis, our data showed significantly reduced expression of all 3 effector mechanisms from BRQ-treated cultures relative to the vehicle-treated controls. Together, these data support the hypothesis that DHODH inhibitors downregulate the expression of MDSC-associated immune-suppressive and protumorigenic genes, while promoting myeloid maturation.

### BRQ treatment alters MDSC phenotype and function in vivo in models of TNBC.

**Figure 3:**
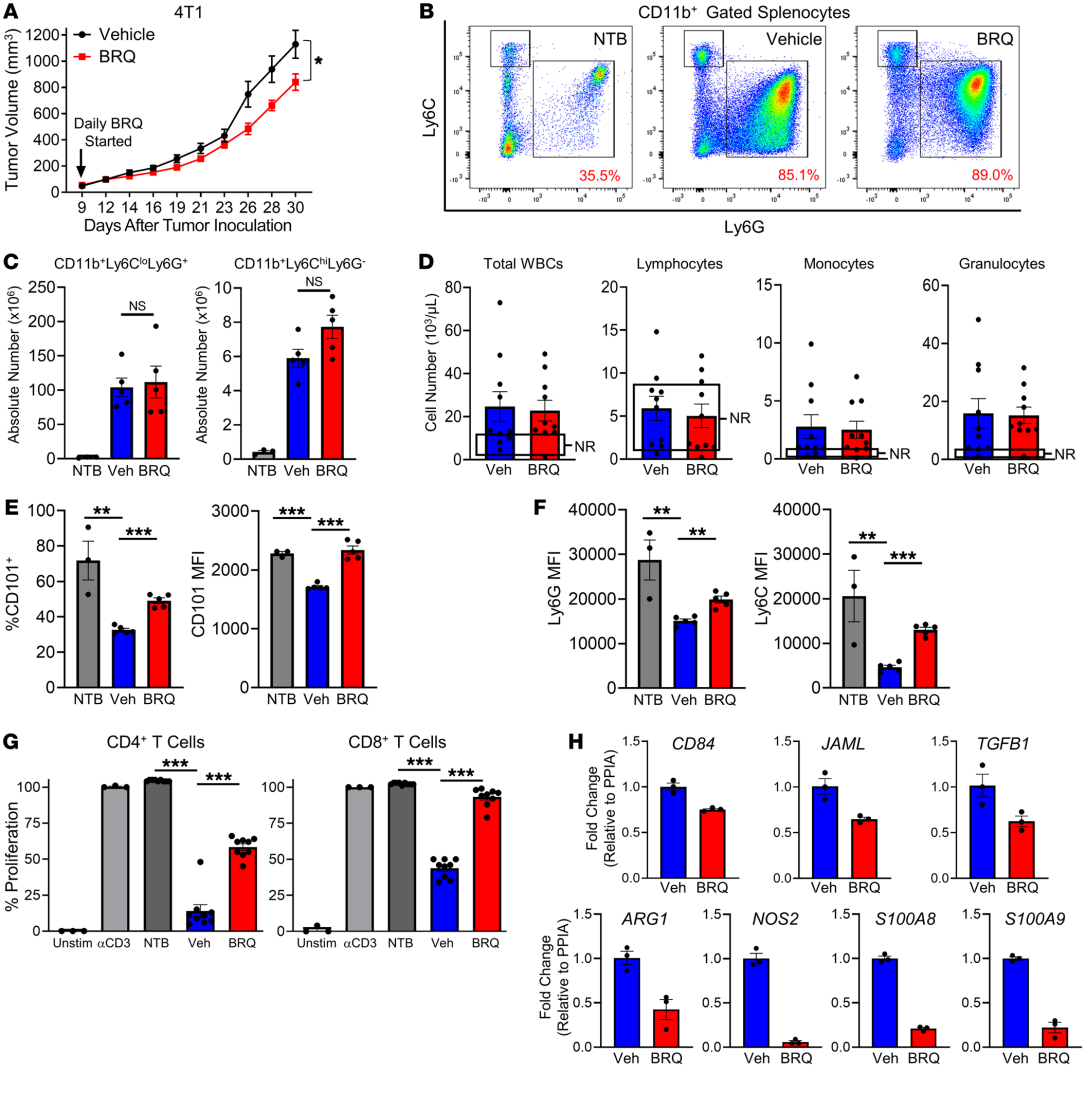
BRQ inhibits MDSC function in vivo. (**A**) 4T1 tumor growth in mice treated with vehicle or daily injections of 10 mg/kg BRQ (from Clear Creek), starting on day 9 after implantation. (**B**) Flow cytometric analysis of Ly6C and Ly6G expression on the gated splenic CD11b^+^ cells. (**C**) Absolute numbers of splenic CD11b^+^Ly6C^lo^Ly6G^+^ and CD11b^+^Ly6C^hi^Ly6G^–^ cells from **B**. (**D**) Numbers of WBCs, lymphocytes, monocytes, and granulocytes per microliter of peripheral blood. Boxed areas indicate the normal range (NR). (**E**) Expression of CD101 on the gated splenic CD11b^+^Ly6C^lo^Ly6G^+^ cells. The percentage of CD101^+^ cells and CD101 MFI are shown. (**F**) Expression of Ly6G and Ly6C on the gated CD11b^+^Ly6C^lo^Ly6G^+^ cells. (**G**) Proliferation of activated CD4^+^ or CD8^+^ T cells following coculturing with PMN-MDSCs isolated as in Figure 2C from 4T1-bearing animals. (**H**) Fold change in gene expression in purified MDSCs from the spleen (determined by RT-qPCR and normalized to *PPIA*). The Miltenyi Mouse Myeloid-Derived Suppressor Cell Isolation Kit was used for *CD84*, *JAML*, and *TGFB1*, and the STEMCELL EasySep Mouse MDSC isolation kit was used for *ARG1*, *NOS2*, *S100A8*, and *S100A9*. Data in all panels are presented as the mean ± SEM of the indicated data points. (**A**–**G**) *n* = 3–10 mice/group. Data in **H** are presented as the mean ± SEM of triplicate determinations and are representative of experiments using up to 3 separate mice, with similar results. *P < 0.05, **P < 0.01, and ***P < 0.001, by 2-sided Wald test (**A**) and unpaired *t* test (**C**–**G**).

We extended our studies to test whether BRQ exerted antitumor activity and/or reduced MDSC development or function in vivo. BRQ was chosen over Lef for the in vivo studies, given its higher potency, aqueous solubility, and relative ease of i.p. administration (26, 45). We used 2 metastatic TNBC models, 4T1 and E0771.ML-1 (a metastatic variant of the parental E0771 tumor cell line), which induce robust MDSC responses, especially of the PMN-MDSC subset (18, 46, 47). 4T1 and E0771.ML-1 tumor cells were orthotopically implanted into syngeneic female BALB/c and C57BL/6 mice, respectively. BRQ treatment (10 mg/kg i.p., given daily) was initiated on day 9, when tumors became measurable in mice bearing the 4T1 (Figure 3A) or E0771.ML-1 (Supplemental Figure 3A) tumors. In both models, BRQ had minimal to modest effects on the rates of tumor growth. We used the 4T1 model, a prototypical model for studies of MDSC tumor biology, to further investigate the effects of BRQ on MDSC frequencies. We found that BRQ did not significantly reduce splenomegaly (data not shown) or the accumulation of both PMN- and M-MDSCs in the spleen (a major reservoir for MDSCs) compared with spleens from the vehicle control–treated mice (Figure 3, B and C). Likewise, there were no significant differences in peripheral blood granulocytes, other leukocytes, RBCs, or platelets when comparing vehicle- and BRQ-treated tumor-bearing mice (Figure 3D and Supplemental Figure 3B).

Although BRQ did not seem to affect MDSCs quantitatively, these studies did not exclude the possibility that BRQ affected MDSCs qualitatively. Here, we performed 3 types of analyses, focusing on changes in CD101 expression, immune suppression, and gene expression. In support of our in vitro findings (Figure 2), we observed a higher percentage of CD101^+^ cells within splenic PMN-MDSCs from BRQ-treated mice compared with the PMN-MDSCs from vehicle control–treated mice. Similarly, we observed increased levels of CD101 expression, as measured by MFI values (Figure 3E and Supplemental Figure 3C). As with our in vitro analysis (Figure 2), we did not detect CD101 expression on the monocytic subset (Supplemental Figure 3C**)**. Interestingly, during this analysis, we observed an increase in the MFI values for the Ly6G and Ly6C markers on the PMN-MDSCs (Figure 3F and Supplemental Figure 3D) in response to BRQ treatment. Ly6G expression on granulocytes can be separated into Ly6G^hi^ and Ly6G^int^ fractions, the former of which represents a more mature cell population (48). Granulocytes expressing lower levels of Ly6C are expanded in relation to tumor growth (49). Thus, these studies suggested that increased expression of Ly6G and/or Ly6C may be indicative of granulocyte maturation. Our data showing that BRQ treatment of 4T1 tumor–bearing (4T1-bearing) mice resulted in significantly higher levels of Ly6G and Ly6C by the PMN-MDSCs (Figure 3F) are consistent with maturation. The potential importance of the association between Ly6G or Ly6C expression and myeloid maturation was strengthened by the finding that the expression of each marker was directly associated with CD101 expression (Supplemental Figure 3, D and E). We observed this relationship even in a group-to-group comparison, in which the CD101/Ly6G or CD101/Ly6C values were higher in the BRQ-treated, tumor-bearing mice compared with the vehicle controls. In contrast, we did not observe such a relationship between CD101 and CD11b expression, suggesting specificity for the association between CD101 and Ly6G or Ly6C in response to BRQ treatment (Supplemental Figure 3, D and E).

We examined whether BRQ treatment in vivo altered MDSC-suppressive activity. For these experiments, PMN-MDSCs were purified from the spleens of BRQ- or vehicle-treated, 4T1-bearing mice or of the NTB controls. We focused on PMN-MDSCs, as they represent the dominant tumor-induced MDSC subset, and our morphologic and immunophenotyping data supported the notion that PMN-MDSCs were a target of BRQ treatment. As expected, PMN-MDSCs isolated from the vehicle-treated, 4T1-bearing mice inhibited CD4^+^ and CD8^+^ T cell proliferation compared with phenotypically matched cells isolated from the NTB controls. In contrast, PMN-MDSCs from the BRQ-treated, 4T1-bearing mice exhibited a significantly diminished capacity to inhibit CD4^+^ or CD8^+^ T cell proliferation compared with PMN-MDSCs obtained from the vehicle-treated counterparts (Figure 3G). Consistent with a loss of MDSC-suppressive potency, we observed a reduction in the expression of the MDSC-associated markers *CD84*, *JAML*, *ARG1*, *NOS2*, *S100A8*, and *S100A9,* as well as the immune-suppressive factor *TGFB* (Figure 3H). Despite these changes, single-agent BRQ was mildly effective in suppressing primary tumor growth in 2 independent TNBC models (Figure 3A and Supplemental Figure 3A).

### BRQ enhances the antitumor activity of programmed cell death 1 blockade and reduces spontaneous lung metastases.

**Figure 4:**
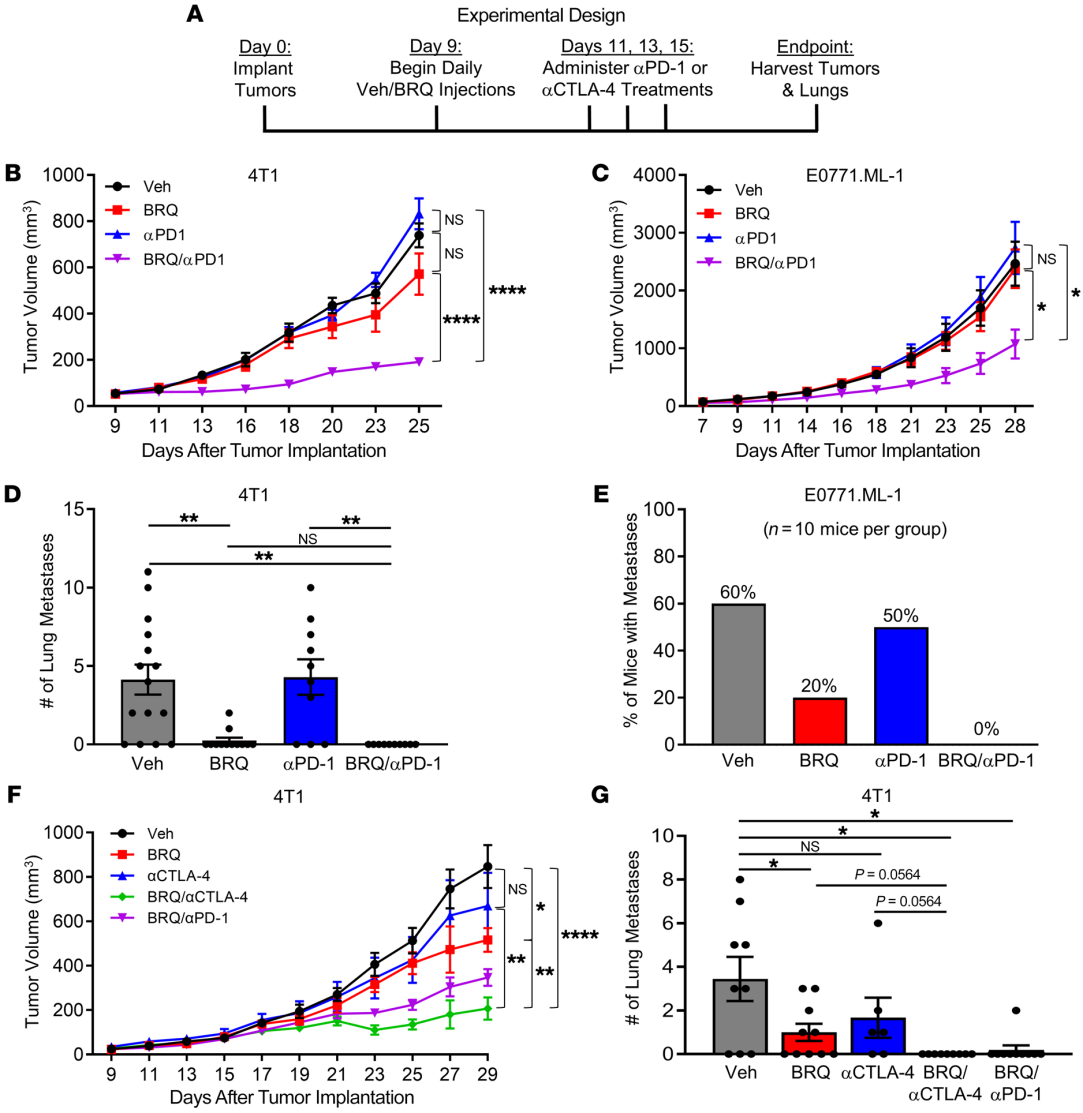
BRQ enhances the antitumor efficacy of anti–PD-1 therapy and reduces spontaneous lung metastases. (**A**) Scheme of the in vivo treatment of tumor-bearing mice with or without BRQ (from Clear Creek). (**B**) 4T1 and (**C**) E0771.ML-1 tumor growth in mice treated with vehicle, BRQ (10 mg/kg BRQ), anti–PD-1 (200 μg/injection), or the combination of BRQ and anti–PD-1, as shown in **A**. Tumors in the vehicle, BRQ alone, and anti–PD-1 alone treatment groups all had significantly higher growth rates than did tumors in the BRQ plus anti–PD-1 combination group. (**D**) Number of spontaneous lung metastases in 4T1-bearing mice receiving the treatments indicated in **B**. (**E**) Percentage of E0771.ML-1–bearing mice that had spontaneous lung metastases (*n* = 10 mice/group) after receiving the treatments indicated in **C**. (**F**) 4T1 tumor growth in mice treated with vehicle, BRQ (10 mg/kg), BRQ plus anti–PD-1 (200 μg/injection), anti–CTLA-4 (100 μg/injection), or BRQ plus anti–CTLA-4 as shown in **A**. Results for the combination treatment groups (BRQ plus anti–PD-1 and BRQ plus anti–CTLA-4) were not statistically different. (**G**) Number of spontaneous lung metastases in 4T1-bearing mice receiving the indicated treatments, as in **F**. Data are presented as the mean ± SEM of the indicated data points and represent 6–10 mice/group. *P < 0.05, **P < 0.01, and ****P < 0.0001, by 2-sided Wald test for the combination treatment group versus the vehicle control or the single-agent treatment groups for the tumor growth curves (**B**, **C**, and **F**) and by unpaired *t* test for lung metastasis data (**D** and **G**).

These data suggested that targeting MDSCs alone may be insufficient to achieve substantial antitumor activity. Thus, we hypothesized that BRQ enhances the therapeutic efficacy of ICIs. To test this hypothesis, we returned to the ICI-resistant 4T1 and E0771.ML-1 TNBC models (50–52). Tumor cells were orthotopically implanted, and mice were treated with either BRQ, anti–programmed cell death 1 (anti–PD-1) mAb, BRQ plus anti–PD-1 mAb, or the appropriate controls (Figure 4A). We focused on anti–PD-1–based therapy because an inhibitor of PD-1 (pembrolizumab) is FDA approved for use in certain populations of patients with TNBC. We found that BRQ or anti–PD-1 mAb alone had little to no effect on primary tumor growth in these 2 TNBC models (Figure 4, B and C, and Supplemental Figure 4, A and B), consistent with our earlier findings (Figure 3) and prior studies on de novo ICI (anti–PD-1) resistance (50, 52). However, BRQ plus anti–PD-1 mAb significantly reduced primary tumor growth in both tumor models, and the therapeutic efficacy of the combination regimen versus the single-agent treatments was synergistic (Figure 4, B and C, and Supplemental Figure 4, A and B). These results show that the combination of BRQ and anti–PD-1 mAb was effective across different cell lines and mouse strains.

Since both tumor models can metastasize to the lung (a common site for human TNBC spread), we examined the impact of mono- or combination therapy on spontaneous lung metastasis. The lungs from either 4T1- or E0771.ML-bearing mice were histologically quantified for metastatic lesions. Interestingly, we observed that BRQ treatment, either as a single agent or combined with anti–PD-1 mAb, significantly decreased lung metastasis (Figure 4, D and E), whereas single-agent anti–PD-1 mAb had no overt antimetastatic effects. Since the E0771.ML-1 model had a smaller number of metastatic nodules than the 4T1 model (*n* < 5 nodules per lung), data in this model are reported as the percentage of mice with evidence of metastases (Figure 4E). Together, these data indicated that BRQ enhanced the antitumor and antimetastatic activity of PD-1–based therapy in 2 ICI-resistant TNBC models. Moreover, we tested whether BRQ treatment could enhance the antitumor and/or antimetastatic activity of anti–CTLA-4 mAb, another ICI, in the 4T1 model (Figure 4, F and G, and Supplemental Figure 4B). As with anti–PD-1 mAb, anti–CTLA-4 mAb alone was ineffective, but it was highly effective when combined with BRQ, resulting in significant antitumor and antimetastatic activity. Statistical analyses further revealed that the BRQ plus anti–CTLA-4 mAb treatment combination was therapeutically comparable to that of the BRQ plus anti–PD-1 mAb combination and was synergistic relative to the single-agent treatments.

### BRQ enhances the efficacy of anti–PD-1 therapy through on-target effects in an MDSC- and CD8^+^ T cell–dependent manner.

**Figure 5:**
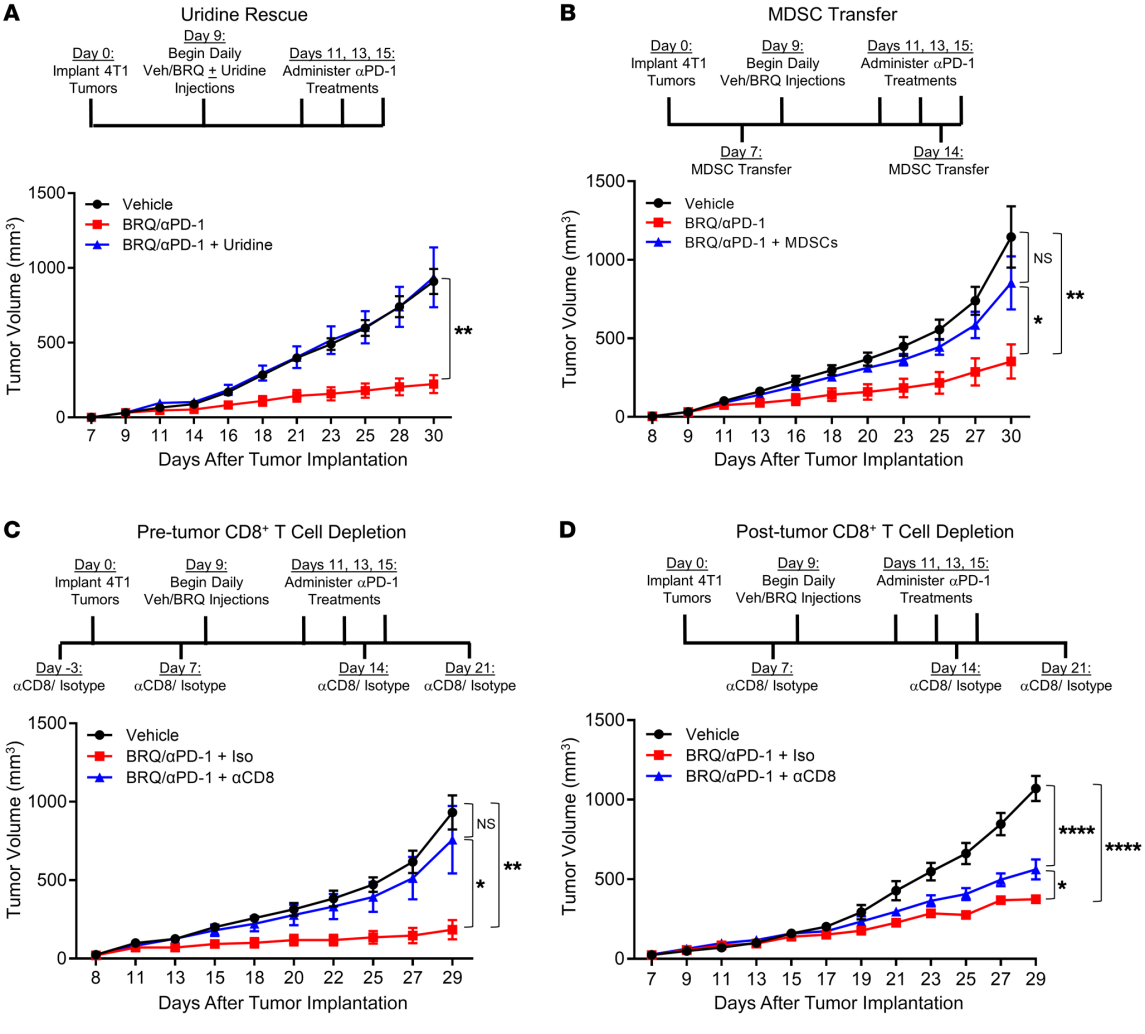
Inhibition of tumor growth by combined BRQ and anti–PD-1 therapy is dependent on depletion of uridine or MDSCs, and the presence of CD8^+^ T cells. (**A**) Experimental scheme and 4T1 tumor growth rates in mice treated with vehicle, BRQ (from Clear Creek) plus anti–PD-1, or BRQ plus anti–PD-1 plus uridine. Uridine (300 mg/kg i.p.) was administered concomitantly with BRQ for the duration of the experiment. (**B**) Experimental scheme and 4T1 tumor growth rates in mice treated with vehicle, BRQ plus anti–PD-1, or BRQ plus anti–PD-1 plus CD11b^+^Gr-1^+^ MDSCs. MDSCs were flow-sorted from the spleens of syngeneic female *Irf8^–/–^* mice (aged 10–12 weeks), and 1 × 10^6^ cells were administered i.v. on days 7 and 14. (**C** and **D**) Experimental schemes and 4T1 tumor growth rates in mice treated with vehicle, BRQ plus anti–PD-1, or BRQ plus anti–PD-1 plus anti-CD8–depleting antibody. Mice received anti-CD8–depleting antibody or isotype (400 μg/mouse i.p.) at the indicated time points. Mice in **C** received the first dose of anti-CD8–depleting antibody 3 days prior to 4T1 tumor implantation, and mice in **D** received the first dose of anti-CD8–depleting antibody 7 days after 4T1 tumor implantation. Data are presented as the mean ± SEM of multiple determinations (*n* = 5–10 mice/group). *P < 0.05, **P < 0.01, and ****P < 0.0001, by 2-sided Wald test for the combination treatment group versus the vehicle control or the specified experimental group in **A**–**D**.

We investigated potential mechanisms by which BRQ treatment enhances ICI efficacy, with a focus on 3 questions: Is the effect of BRQ (a) on target, (b) MDSC dependent, and (c) CD8^+^ T cell dependent (Figure 5 and Supplemental Figure 5)? First, we examined whether the effects of BRQ treatment in vivo were specific to inhibition of DHODH. As described earlier, the effects of BRQ treatment can be reversed by the addition of uridine, indicating that cells can escape the effects of DHODH inhibition through uridine salvage pathways (40, 45). Therefore, we tested whether the effects of BRQ plus anti–PD-1 mAb could be reversed by uridine supplementation. We treated 4T1-bearing mice with the combination regimen, with or without concomitant administration of uridine. The ability of the combination therapy to inhibit tumor growth was significantly abrogated by uridine supplementation (Figure 5A and Supplemental Figure 5, B and F), indicating that the effects of BRQ in this therapeutic paradigm acted through inhibition of DHODH.

Second, we tested our hypothesis that BRQ inhibits MDSC biogenesis at an early stage in myeloid cell differentiation. We reasoned that if BRQ is acting on MDSC biogenesis, then restoring peripheral MDSC numbers through adoptive transfer should antagonize BRQ efficacy. Conversely, if BRQ targets the MDSC itself, then the adoptive transfer of MDSCs should have a minimal impact. To test this idea, we used MDSCs (CD11b^+^Gr-1^+^ cells) isolated from the spleens of *Irf8^–/–^* mice, a model that generates high numbers of suppressive splenic MDSCs, even in the absence of tumor implantation. This observation was derived from our earlier work demonstrating that IRF8 is an integral negative regulator of MDSC biogenesis in the BM (14). We predicted that *Irf8^–/–^* MDSCs would be refractory to BRQ because the absence of IRF8 would preclude myeloid differentiation, which is supported by the observation that *Irf8^–/–^* MDSCs retain their suppressive activity against CD4^+^ and CD8^+^ T cells following BRQ treatment (performed as in Figure 1 and Supplemental Figure 5A). We then treated 2 groups of 4T1-bearing mice with the combination therapy, and 1 group received the adoptive transfer of splenic *Irf8^–/–^* CD11b^+^Gr-1^+^ cells at 2 separate time points. The adoptive transfer of these myeloid cells antagonized the therapeutic benefits of the combination regimen and restored tumor growth compared with the vehicle control–treated mice (Figure 5B and Supplemental Figure 5, C and G), suggesting that the effects of BRQ in the combination therapy were MDSC dependent.

Third, we investigated the contribution of the CD8^+^ T cell population in response to the BRQ plus anti–PD-1 mAb combination therapy, which has been shown to be important for the efficacy of PD-1 blockade (53). We examined the role of CD8^+^ T cells under 2 settings of CD8^+^ T cell depletion: before and after tumor implantation. First, mice were treated with a CD8^+^ T cell–specific depleting mAb or an isotype control prior to implantation of 4T1 cells (Figure 5C and Supplemental Figure 5E for the specificity and efficiency of CD8^+^ T cell depletion in the peripheral blood). 4T1-bearing mice were then treated with combination therapy. The depletion of CD8^+^ T cells significantly negated the effects of the combination therapy and restored tumor growth (Figure 5C and Supplemental Figure 5, D and H). Next, in a more aggressive setting, CD8^+^ T cells were depleted 7 days after tumor implantation, followed by treatment with the BRQ plus anti–PD-1 mAb regimen (Figure 5D and Supplemental Figure 5I). Our data showed that the depletion of CD8^+^ T cells under these conditions still led to a significant loss in therapeutic efficacy, consistent with the interpretation that the combination regimen was CD8^+^ T cell dependent. However, CD8^+^ T cell depletion before tumor implantation appeared to be a more effective strategy to reverse therapeutic efficacy compared with post-tumor implantation, suggesting that the timing of depletion was relevant to the therapeutic outcome. Together, these data support the hypotheses that the antitumor effects of BRQ when combined with anti–PD-1 mAb are reliant on de novo pyrimidine synthesis, governed by the loss of MDSCs, and CD8^+^ T cell dependent.

### BRQ alters the tumor immune microenvironment.

**Figure 6:**
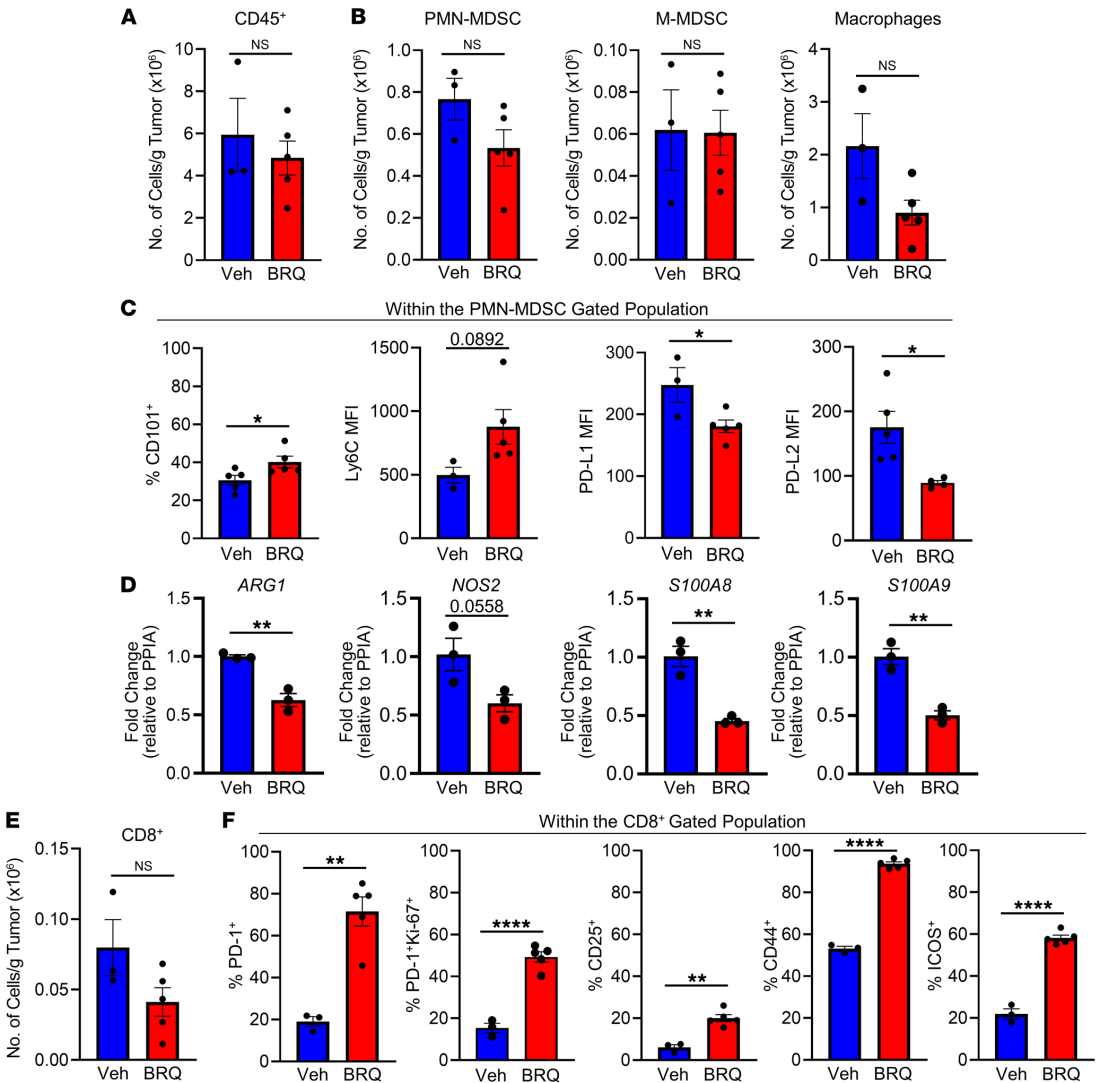
BRQ increases the activation state of CD8^+^ T cells and the maturation of PMN-MDSCs within the TME. 4T1-bearing mice were treated with or without BRQ (Clear Creek), as shown in the preceding figures. At the experimental endpoint (average tumor volume = 600–800 mm^3^), tumors were removed and analyzed by flow cytometry. (**A**) Absolute numbers of CD45^+^ cells, (**B**) PMN-MDSCs (left), M-MDSCs (middle), and macrophages (far right) per gram of tumor tissue. (**C**) Expression of CD101 (percentage), Ly6C (MFI), and PD-L1 and PD-L2 (MFI) by gated PMN-MDSCs. (**D**) CD11b^+^Gr-1^+^ MDSCs were recovered from individual 4T1 tumors using the STEMCELL EasySep Mouse MDSC isolation kit and analyzed by RT-qPCR for the indicated genes. (**E**) Absolute number of CD8^+^ T cells per gram of tumor tissue. (**F**) Percentage of CD8^+^ T cells expressing PD-1, PD-1 and Ki-67, CD25, CD44, or ICOS. Data are presented as the mean ± SEM of multiple determinations (shown as individual data points; *n* = 3–5 mice/group). Data in **D** are presented as the mean ± SEM of triplicate determinations and are representative of 2–3 separate mice, with similar results. *P < 0.05, **P < 0.01, and ****P < 0.0001, by unpaired *t* test.

To further gain insights into the effects of BRQ on the MDSC and CD8^+^ T cell responses, we turned our attention to the TME in the 4T1 tumor model (Figure 6 and Supplemental Figure 6). Here, we focused on BRQ treatment alone, given daily for 14 days after tumors first became measurable. We found that BRQ treatment relative to the vehicle control did not significantly alter the number of total CD45^+^ leukocytes (Figure 6A and Supplemental Figure 6A). As we observed in the spleen (Figure 3), the numbers of myeloid cells within the TME, including PMN-MDSCs, M-MDSCs, and macrophages, were unaffected by BRQ treatment (Figure 6B and Supplemental Figure 6B), consistent with the lack of a quantitative effect. Therefore, we next examined these PMN-MDSCs for phenotypic evidence of myeloid maturation, as in our earlier data (Figures 2 and 3). PMN-MDSCs within the TME of BRQ-treated mice showed significantly higher expression of CD101 and a trend toward higher Ly6C expression (Figure 6C), consistent with our earlier findings in the spleen. Additionally, there was a reduction in both programmed death ligand 1 (PD-L1) and PD-L2 MFIs in PMN-MDSCs from BRQ-treated tumor-bearing mice (Figure 6C), supporting the notion that these PMN-MDSCs are less suppressive relative to the vehicle-treated controls. To further assess the immune-suppressive phenotype of PMN-MDSCs in the TME, we performed reverse transcription quantitative PCR (RT-qPCR) to evaluate gene expression of *ARG1*, *NOS2*, *S100A8*, and *S100A9* (Figure 6D). PMN-MDSCs from BRQ-treated tumor-bearing mice had decreased expression of all these genes compared with the vehicle-treated control mice, suggesting that PMN-MDSCs within the TME of BRQ-treated mice had a reduced suppressive phenotype.

We next investigated the presence and activation status of CD8^+^ T cells within the TME (Figure 6, E and F), building on our earlier findings that these cells are critical for the efficacy of the combination therapy (Figure 5). As with the myeloid cells, the total number of CD8^+^ T cells was unchanged relative to the vehicle control (Figure 6E). However, CD8^+^ T cells within the TME of BRQ-treated mice showed significantly higher expression of PD-1 and the activation markers CD25, CD44, and ICOS (Figure 6F and Supplemental Figure 6A). The increase in PD-1 expression under these conditions was likely reflective of immune activation, since a significant increase was observed in PD-1^+^ cells coexpressing the proliferation marker Ki-67 (Figure 6F). These data indicate that BRQ treatment translates to beneficial changes in the immune (i.e., myeloid and CD8^+^ T cell) compartment of the TME, priming cells to respond to ICI therapy.

### BRQ suppresses MDSC biogenesis from early-stage BM myeloid progenitors.

To further test our hypothesis that BRQ suppresses MDSC biogenesis by targeting early-stage myeloid progenitors, we performed spectral flow cytometry to quantify populations of mature myeloid cells and hematopoietic stem and progenitor cells (HSPCs) in 4T1-bearing mice treated with vehicle or BRQ using the same schedule used for the TME analysis (Supplemental Figure 7, A and B). BRQ treatment resulted in increased numbers of CD101^+^ granulocytes in the BM, but no significant effect on BM monocyte or myeloid DC populations (Supplemental Figure 7, C and D). BRQ treatment resulted in modest increases in several HSPC populations including GMPs (Supplemental Figure 7E).

**Figure 7:**
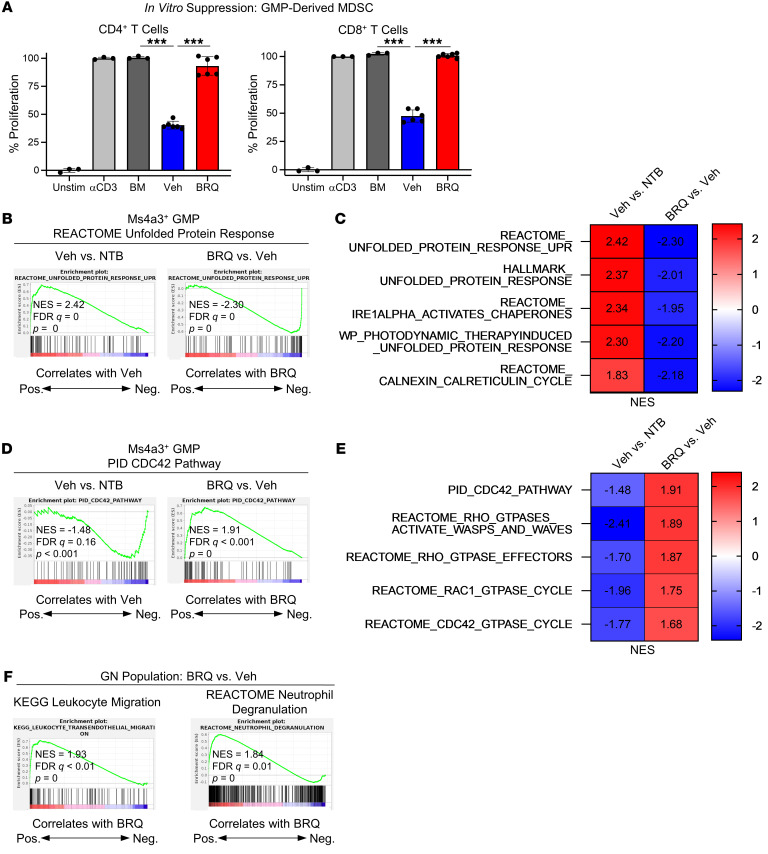
BRQ inhibits the development of MDSCs from BM myeloid progenitors. (**A**) Proliferation of activated CD4^+^ or CD8^+^ T cells following coculturing with GMP-derived MDSCs. Unstim, unstimulated. (**B**) scRNA-Seq experiments were performed on c-Kit^+^ BM cells isolated from NTB mice or 4T1-bearing mice treated with vehicle or BRQ. For each experimental group, 3 biologic replicates were pooled. GSEA of the REACTOME UPR pathway comparing Veh-GMPs with NTB-GMPs (left) and BRQ-GMPs with Veh-GMPs (right). (**C**) Heatmap showing up- and downregulation of UPR pathways (*P* < 0.01, FDR < 0.25) in GMPs, based on the indicated comparisons. (**D**) GSEA of the Pathway Interaction Database (PID) Cdc42 signaling pathway comparing Veh-GMPs versus NTB-GMPs (left) and BRQ-GMPs versus Veh-GMPs (right). (**E**) Heatmap showing up- and downregulation of pathways related to RhoGTPase signaling (*P* < 0.01, FDR < 0.25) in GMPs, based on the indicated comparisons. (**F**) GSEA of the Kyoto Encyclopedia of Genes and Genomes (KEGG) leukocyte migration and the REACTOME neutrophil degranulation pathways comparing BRQ-GNs and Veh-GNs. Data in **A** are presented as the mean ± SEM of triplicate determinations from 2 separate mice. ***P < 0.001, by unpaired *t* test. In **B**, **D**, and **F**, the normalized enrichment score, FDR (*q*), and nominal *P* value are shown. NES, normalized enrichment score; Neg., negative; Pos., positive.

Given that BRQ had a modest quantitative effect on GMPs, we investigated whether BRQ had a qualitative effect on GMPs by testing whether BRQ inhibited the ability of GMPs to differentiate into MDSCs. GMPs sorted from the BM of 4T1-bearing mice were treated with vehicle or BRQ and cultured in vitro with G-CSF to drive differentiation toward PMN-MDSCs (GMP-derived MDSCs) (18). Treatment of 4T1-bearing mice with BRQ resulted in a trend toward increased expression of CD101 by the GMP-derived MDSCs (Supplemental Figure 8A). We then tested GMP-derived MDSCs for their immune-suppressive activity (Figure 7A). GMPs from 4T1-bearing mice treated with BRQ showed a reduced capacity to develop into immune-suppressive MDSCs compared with 4T1-bearing mice treated with vehicle, further suggesting that BRQ suppresses MDSC biogenesis.

To further investigate the effects of BRQ on hematopoietic progenitors, specifically the GMP population, we performed single-cell gene expression analysis on c-Kit^+^ BM cells isolated from NTB mice and 4T1-bearing mice treated with vehicle or BRQ (Supplemental Figure 8B). Cell annotation was performed using the ImmGen Database (54). We further refined the GMP population using the expression of *Ms4a3*, a recently characterized marker of GMPs (Supplemental Figure 8C) (55). We performed gene set enrichment analysis (GSEA) to compare pathway signatures in Ms4a3^+^ GMPs isolated from vehicle-treated 4T1-bearing mice (Veh-GMPs) with NTB mice (NTB-GMPs) (Supplemental Tables 1 and 2). We observed upregulation of a pyrimidine metabolism signature in Veh-GMPs compared with NTB-GMPs, suggesting increased pyrimidine metabolism in these progenitors (Supplemental Figure 8D and Supplemental Table 1) and providing additional support for our hypothesis that this pathway represents a metabolic vulnerability targetable by BRQ.

To identify potential molecular mechanisms of action for BRQ, we focused on pathways where BRQ reversed tumor-driven effects on the GMP population (based on the Veh-GMP versus NTB GMP comparison) and that were known to be involved in MDSC biology or hematopoiesis (Supplemental Tables 3 and 4). Overall, we observed that GMPs from 4T1-bearing mice treated with BRQ (BRQ-GMPs) exhibited downregulation of ribosomal pathways compared with Veh-GMPs (Supplemental Table 4), consistent with a previously reported effect of DHODH inhibition (56) and further supporting our notion that BRQ is acting in the BM. Veh-GMPs exhibited upregulation of several pathways linked to the unfolded protein response (UPR) compared with NTB-GMPs (Figure 7, B and C). The UPR consists of multiple pathways that are activated upon accumulation of unfolded and misfolded proteins to restore proteostasis; additionally, the upregulation of UPR pathways is necessary for the suppressive function of MDSCs (13, 57–59). Treatment with BRQ reversed this effect, suggesting that reduced UPR activity may be 1 mechanism by which BRQ inhibits MDSC biogenesis.

We also examined pathways that were upregulated in BRQ-GMPs compared with Veh-GMPs. Here, we unexpectedly found that pathways regulated by members of the RhoGTPase family (Cdc42, Rac1, and Rhoa) were upregulated in BRQ-GMPs compared with Veh-GMPs (Figure 7, D and E, and Supplemental Table 3). RhoGTPases are a family of small G proteins that operate across multiple signal transduction pathways and cellular processes, including hematopoiesis and neutrophil migration and effector function (60–64), although a role in MDSC biogenesis has not been previously described. We found that these pathways were downregulated in Veh-GMPs compared with NTB-GMPs, suggesting that BRQ treatment reversed this effect and upregulated RhoGTPase signaling. Additionally, we observed that the UPR pathway genes *CHOP* and *DNAJB11* showed decreased expression in MDSCs within the TME of 4T1-bearing mice treated with BRQ compared with mice treated with vehicle (Supplemental Figure 8E). Conversely, the expression of *CDC42* was increased in MDSCs within the TME of 4T1-bearing mice treated with BRQ. These results were consistent with the single-cell RNA-Seq (scRNA-Seq) data and suggest that the effect of BRQ on gene expression in the BM progenitors was maintained in MDSCs within the TME.

To compare the differentiation trajectories of BM progenitors in 4T1-bearing mice treated with BRQ compared with those treated with vehicle, we performed pseudotime analysis on the scRNA-Seq data sets. We observed no marked difference in the trajectories between the treatment conditions (Supplemental Figure 8F). We then used GSEA to compare pathway signatures in the BM progenitor populations that lie at the terminus of the differentiation trajectories (annotated as “GN” in Supplemental Figure 8B) and found that BRQ treatment resulted in an upregulation of pathways associated with leukocyte migration and neutrophil effector function (Figure 7F and Supplemental Tables 5 and 6), suggesting that these cells may exhibit a more mature granulocytic phenotype. Together, these results suggest that BRQ alters multiple GMP pathways and point toward several mechanisms that may underlie MDSC biogenesis.

### BRQ promotes myeloid maturation and inhibits the expression of an immune-suppressive phenotype in a human BM culture system.

**Figure 8:**
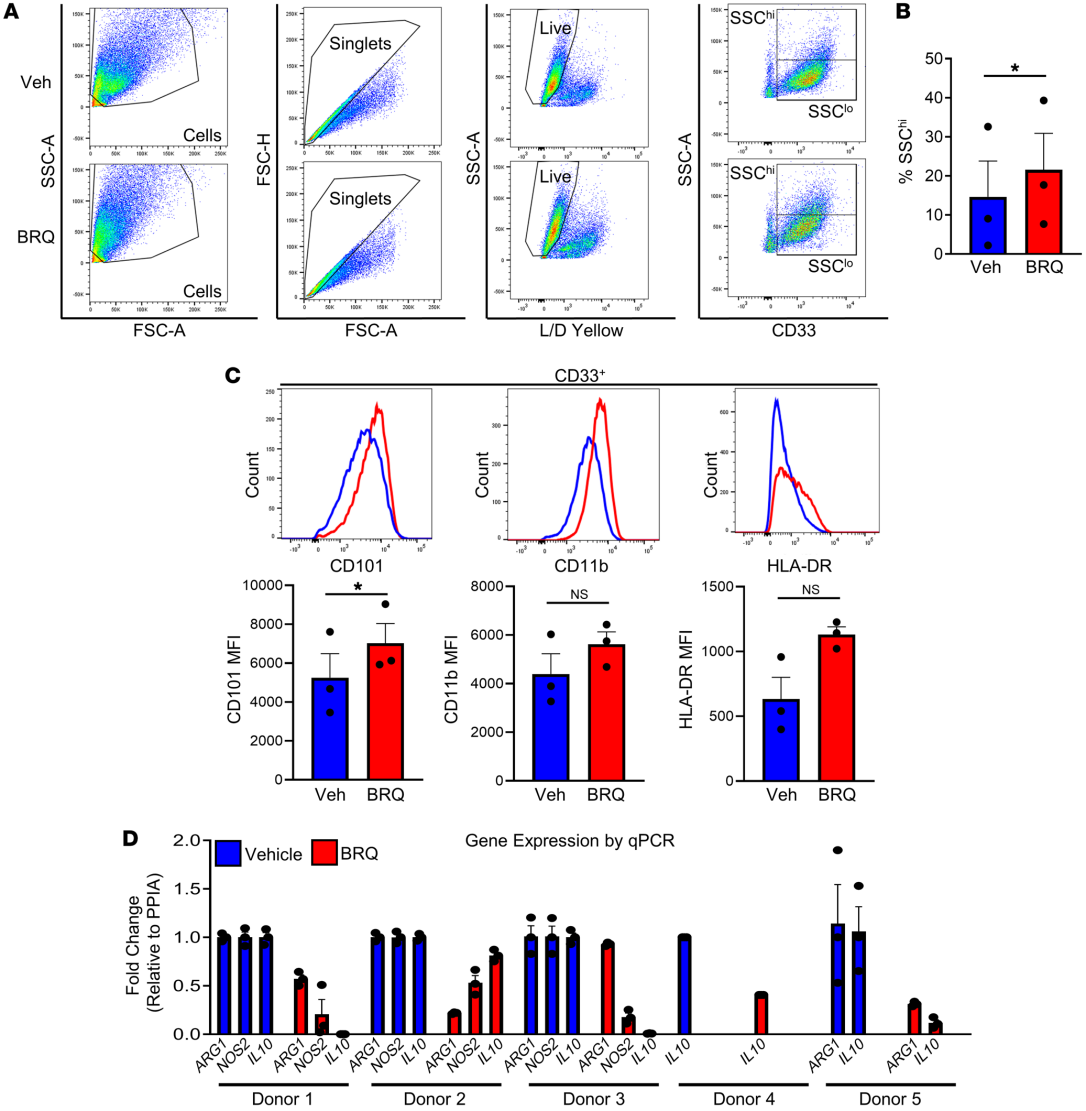
BRQ inhibits the development of an immunosuppressive phenotype in human myeloid cells. Human BM cells were cultured for 96 hours with recombinant human GM-CSF (rhGM-CSF) and rhG-CSF (40 ng/mL each) plus 1 μM BRQ (from Clear Creek) or vehicle. (**A**) Flow cytometric analysis of CD33 expression and SSC properties (84%–88% CD33^+^ with or without BRQ treatment). FSC-A, forward scatter area; FSC-H, forward scatter height. (**B**) Percentage of CD33^+^ myeloid cells exhibiting high SSC (SSC^hi^). (**C**) Histograms depicting CD101, CD11b, or HLA-DR expression by the gated CD33^+^ cells and quantification of the MFI values for CD101, CD11b, and HLA-DR by the gated CD33^+^ cells. Data are presented as the mean ± SEM of 3 separate donors. (**D**) Fold change in expression of *ARG1*, *NOS2*, and *IL10* (determined by RT-qPCR analysis) in cultured human BM cells. Expression was normalized to *PPIA* and is depicted for each donor (*n* = 5 separate donors, including the 3 donors from **A**–**C**). The absence of a bar indicates no detectable signal for the expression of either *ARG1*, *NOS2*, or *IL10*. Donor 1: female, age 29 years; donor 2: female, age 48 years; donor 3: female, age 13 years; donor 4: male, age 7 years; donor 5: male, age 12 years. *P < 0.05, by paired *t* test (**B** and **C**).

To validate our preclinical findings, we developed a human BM culture system akin to that used to produce murine MDSCs (Figure 1). Here, we cultured healthy donor unfractionated BM cells with human recombinant GM-CSF and G-CSF in the absence or presence of BRQ (Figure 8). Our analysis focused on the broader issues of myeloid maturation or activation, and we initially focused on the phenotype of the resultant myeloid cell populations based on CD33 expression, a pan-myeloid cell-surface marker (Figure 8A). As with the murine studies, cytokine treatment of human BM cells yielded similar frequencies of myeloid cells with or without BRQ, based on CD33 expression (Figure 8A). In humans, mature myeloid cells, particularly neutrophils, can be distinguished in part from immature myeloid cells (including MDSCs) on the basis of their side scatter (SSC) profile, with the former cell population being SSC^hi^ and the latter population being SSC^lo^ (65). Among 3 separate donors, we observed a significant increase in the SSC^hi^ population of the BRQ-treated CD33^+^ cells compared with the vehicle-treated control cells (Figure 8, A and B).

Next, we examined the expression of the myeloid markers CD11b and HLA-DR as well as CD101 on the CD33^+^-gated cell population (Figure 8C). In contrast to the expression of CD11b and HLA-DR, the expression of CD101 was significantly higher on the BRQ-treated CD33^+^ cells than on the vehicle-treated control cells. The increase in CD101 expression was consistent with our murine studies. Last, we investigated whether BRQ altered the expression of genes associated with immune-suppressive function. Here, we analyzed a total of 5 donors and found that BRQ treatment reduced the expression of *ARG1*, *NOS2*, and/or *IL10* (Figure 8D and Supplemental Figure 9A). Although these patterns of gene expression varied from donor to donor, we observed a reduction in at least 1 or more of these genes among all donors. Together, these data suggested that BRQ can act in the BM to diminish immune-suppressive features of both the murine and human myeloid response.

## Discussion

In this study, we focused on the underexplored concept of differentiation therapy to mitigate MDSC development and function to sensitize ICI-resistant tumor models to PD-1 blockade. Prior strategies to eliminate MDSCs or to inhibit their function have largely focused on targeting preexisting MDSCs in the circulation or the TME. Our group and others have demonstrated that MDSC biogenesis occurs through a process of dysregulated hematopoiesis that originates with early myeloid progenitors, such as GMPs, in the BM (2, 18, 21). We hypothesized that strategies targeting early myeloid progenitors in the BM would simultaneously reduce MDSC burden while preventing their replenishment. Our in silico observation that the pyrimidine synthesis pathway is upregulated in myeloid progenitors in tumor-bearing mice, combined with prior reports demonstrating that inhibiting pyrimidine synthesis by targeting DHODH induces differentiation of immortalized and leukemic myeloid progenitors (45), provided a strong rationale for testing whether a DHODH inhibitor such as BRQ would suppress MDSC biogenesis.

From these studies, we demonstrated that treatment with BRQ (a) inhibited the immune-suppressive activity of murine MDSCs, which was accompanied by enhanced myeloid maturation, especially affecting the PMN-MDSC subset; (b) showed significant antitumor and antimetastatic activities when combined with the ICI anti–PD-1 mAb in ICI-resistant mouse mammary TNBC models; (c) acted in the BM and reversed tumor-induced MDSC biogenesis originating from early myeloid progenitors; (d) boosted the antitumor activity of anti–PD-1 mAb via MDSC- and CD8^+^ T cell–dependent mechanisms; and (e) suppressed pathways, such as the UPR, that are known inducers of MDSC activity in the BM, potentially revealing mechanisms of MDSC biogenesis. We propose a model in which the ability of BRQ to enhance ICI efficacy can be mediated through an immune mechanism by which BRQ suppresses MDSC function that in turn enables the activation of CD8^+^ T cells (Supplemental Figure 9, B and C). Thus, it is important to point out that our findings regarding the impact of BRQ on improving the efficacy of ICIs are limited to cancer types in which MDSCs are a relevant determinant to the disease process or therapeutic response. Our observations that BRQ also improved anti–CTLA-4 mAb–based therapy suggests that the therapeutic efficacy of the BRQ and anti–PD-1 mAb combination is not necessarily unique to that combination and potentially has broader implications for other ICIs or immunotherapies.

The role of MDSCs is supported by our findings that BRQ reduced the immunosuppressive function of MDSCs using in vitro and in vivo approaches that measured multiple readouts for MDSC activity. Furthermore, we showed that the adoptive transfer of preexisting MDSCs (using *Irf8^–/–^* MDSCs as a model system) was able to reverse the antitumor efficacy of the BRQ plus anti–PD-1 mAb combination. Immunophenotyping showed that BRQ treatment did not have profound effects on the numbers of MDSCs, suggesting that the primary effect of BRQ was qualitative as opposed to quantitative. Thus, we hypothesized that BRQ treatment reduces the MDSC burden through a promotion of maturation rather than elimination, which is supported by our observation that BRQ treatment resulted in signatures associated with mature neutrophil function in BM progenitors. This hypothesis is strengthened by our findings that BRQ treatment increased the frequency of segmented neutrophils (a morphologic marker of maturation) and upregulated the expression of CD101, a recently discovered marker of mature granulocytes that is associated with reduced protumorigenic function (41). Future work is warranted, however, to determine in detail the functionality of the CD101^hi^ cell population induced by BRQ. Since PMN-MDSCs are the predominant MDSC subtype in our preclinical models and in patients with breast cancer (46, 66), we focused our studies on this population. BRQ could also affect the number or function of M-MDSCs or other myeloid cell populations, all of which requires a detailed investigation.

Our observation that the adoptive transfer of MDSCs was able to inhibit the effects of the combined BRQ and anti–PD-1 mAb regimen also supports the idea of a functional link between the reduction of MDSC function and restoration of a functional CD8^+^ T cell response. Further evidence for this interpretation comes from our observations that BRQ monotherapy resulted in an increased percentage of CD8^+^ T cells in the TME that exhibited an activated phenotype, including dual expression of PD-1 and Ki-67, suggesting that BRQ can sensitize CD8^+^ T cells to anti–PD-1 mAb-based therapy. Furthermore, intratumoral MDSCs from these same BRQ-treated mice showed reductions of PD-L1 and PD-L2 expression, as well as of *ARG1*, *NOS2*, *S100A8*, and *S100A9*, consistent with the idea that BRQ acted to dampen the immune-suppressive phenotype of these MDSCs. Altogether, these data strengthened the rationale for the combination immunotherapy and provided a potential molecular basis for the efficacy of BRQ plus anti–PD-1 mAb combination. Not surprisingly, we showed that the antitumor efficacy of the BRQ plus anti–PD-1 mAb regimen was dependent on the presence of CD8^+^ T cells. However, it is interesting to note that depleting CD8^+^ T cells prior to tumor implantation was more effective as a proof-of-concept approach to reverse therapeutic efficacy compared with post-tumor implantation. One potential explanation is that the brief window of time that CD8^+^ T cells were still intact in the latter setting was sufficient to mediate an antitumor effect. These observations, combined with our findings that CD8^+^ T cell numbers (in the periphery or TME) were unaffected by BRQ treatment, also allay potential concerns that treatment with BRQ, through its known mechanism of action as an inhibitor of pyrimidine synthesis, could suppress proliferation or antitumor activity of CD8^+^ T cells.

Although our findings did not reveal any overt negative effects of BRQ on the CD8^+^ T cell response in the context of our preclinical cancer models, DHODH inhibitors are being used clinically in the context of autoimmune disorders to dampen autoimmune T cell activity. For example, 2 different DHODH inhibitors are approved for the treatment of rheumatoid arthritis (Lef) and multiple sclerosis (MS) (teriflunomide) (67–69). With respect to MS, a recent study by Tilly and colleagues (67) showed a specific inhibitory effect of teriflunomide on the CD8^+^ T cell compartment. This was characterized by a reduction in homeostatic proliferation, the production of proinflammatory cytokines, and migratory properties of effector memory CD8^+^ T cells, as measured in vitro. In contrast, teriflunomide treatment did not alter the B cell or CD4^+^ T cell compartments. Determining the exact reasons underlying the differential effects of DHODH inhibitors on the CD8^+^ T cell response in our tumor studies versus the work in autoimmunity, however, will require further investigation including detailed studies of how BRQ directly or indirectly affects CD8^+^ T cell biology. In our work, it is possible that DHODH inhibition following BRQ treatment had both an anti-MDSC and an anti-CD8^+^ T cell effect, although the net effect was alleviation of the immune-suppressive TME, enabling an immune attack following anti–PD-1 mAb therapy. Other potential explanations may reflect different disease contexts with unique cellular targets receptive to DHODH inhibition or differences in the dosing, frequency, duration, and scheduling of the treatment regimens.

Although single-agent BRQ did not inhibit primary tumor growth in our preclinical models, single-agent BRQ was able to inhibit lung metastases. This observation is consistent with prior studies from our group and others demonstrating that a reduction of MDSCs has a significant effect on metastatic, but not necessarily primary, tumor growth (14, 70, 71). BRQ may also inhibit metastasis through immune-independent mechanisms (72), and, thus, the contributions of MDSCs and the immune system to the efficacy of BRQ in inhibiting metastatic versus primary tumor growth warrant further study. We hypothesize that BRQ reduces MDSC burden through inhibition of MDSC biogenesis at the progenitor stage. Our finding that BRQ treatment decreased the ability of GMPs to develop into MDSCs supports this hypothesis, but it is possible that BRQ can alter MDSC biogenesis and function at several stages. However, the fact that the adoptive transfer of preexisting MDSCs was able to block the effects of the BRQ plus anti–PD-1 mAb regimen argues against the concept that the effects of BRQ on the MDSC population are entirely attributable to the elimination of MDSCs in the periphery. Our observations that treatment with BRQ reversed tumor-driven changes in the molecular phenotype of the GMPs provide further support for the concept that BRQ targets early myeloid progenitors in the BM. Here, we found that BRQ treatment resulted in the up- and downregulation of several pathways involved in MDSC function and hematopoiesis. Interestingly, we found that various highly expressed genes up- or downregulated by BRQ in the BM progenitors were similarly affected in MDSCs isolated from the TME, suggesting that the effect of BRQ may persist throughout MDSC biogenesis.

The ability of BRQ to suppress MDSC biogenesis at the progenitor stage may result from these pathways working in combination. Thus, the ability of BRQ to upregulate pathways involved in hematopoiesis (e.g., RhoGTPase pathways) may work in concert with the downregulation of pathways that promote MDSC development and function (60–63). The downregulation of UPR pathways in GMPs from mice treated with BRQ is consistent with prior studies demonstrating that the downregulation of UPR pathways reduces MDSC function (13, 57–59). These studies focused on the role of the UPR in MDSCs, but our results suggest the intriguing possibility that the downregulation of UPR pathways may be a key step in early stages of MDSC biogenesis. While the molecular mechanisms of action of BRQ in MDSC biogenesis and hematopoiesis are still incompletely defined, our observations that the efficacy of BRQ was reversed by uridine supplementation are consistent with prior reports that the effects of BRQ are dependent on the inhibition of DHODH (73, 74). Future investigations will also be required to demonstrate the causal links between the inhibition of DHODH and the subsequent inhibition of MDSC biogenesis at the progenitor stage.

ICIs have shown clinical benefits across multiple types of cancer, but these benefits have been largely limited to a subset of patients. Breast cancer, including TNBC, has demonstrated significant resistance to ICIs. The preclinical results reported here suggest that the use of DHODH inhibitors (such as BRQ) can overcome this resistance in part through the inhibition of MDSC biogenesis. The applicability of this approach to the treatment of patients is supported by our observation that BRQ hindered the in vitro development of human MDSC-like populations. However, it is important to point out that these human studies made use of BM biospecimens from healthy individuals and not from patients with cancer. Thus, this work served as a proof of concept to demonstrate that BRQ can act on human myeloid cells and that future studies are warranted to investigate these objectives in detail in patients with cancer. The extensive experience in the clinical use of BRQ gained from prior and current clinical trials (e.g., NCT03760666, NCT03404726, and NCT03451084) suggests that this approach is feasible (29, 75–77). In summary, our studies indicate that targeting the early stages of MDSC biogenesis to improve the efficacy of ICIs or other active or passive immunotherapies is a viable therapeutic strategy that has the potential to significantly benefit patients with cancer and, perhaps, individuals with other chronic inflammatory pathologies in which MDSCs are induced and are thought to constitute a significant determinant of the disease process or therapeutic response.

## Methods

Detailed methods are provided in the Supplemental Methods.

### scRNA-Seq data.

The RNA-Seq data shown in Supplemental Figure 1 were deposited in the NCBI’s Gene Expression Omnibus (GEO) database under accession number GSE193263. The scRNA-Seq data shown in Figure 7 and Supplemental Figure 8 were deposited in the GEO database under accession number GSE190232.

### Study approval.

All studies involving mice were approved under protocols 1108M and 1117M and conducted in accordance with the IACUC of Roswell Park Comprehensive Cancer Center. All human BM specimens were deidentified, and the studies using these specimens were approved under protocol BDR 134520 as nonhuman subject research and conducted in accordance with the IRB of Roswell Park Comprehensive Cancer Center.

## Author contributions

SHC, MJN, and SIA supervised and designed the study. SHC, RAZ, JP, AMA, EDK, BM, and PKS performed the experiments. SHC, RAZ, AMA, EDK, JW, MJN, and SIA analyzed the data. JB and PKS provided essential reagents. SHC, AMA, JW, MJN, HY, and SIA performed and verified statistical analyses. SHC, AMA, PKS, JW, MJN, and SIA wrote the manuscript. JB, MO, and DBS provided feedback and helped edit the manuscript. SHC is listed first as co–first author because of the larger role he played in the original conceptualization. RAZ is listed first as co-second author because of the larger role he played in data analysis.

## Supplementary Material

Supplemental data

## Version 1 12/01/2022

Electronic publication
