# Supplementary material for: Inhibiting the biogenesis of myeloid-derived suppressor cells enhances immunotherapy efficacy against mammary tumor progression
Source: J Clin Invest. 2022 Dec 1;132(23):e158661. doi: 10.1172/JCI158661 (PMC9711879; doi:10.1172/JCI158661)
Supplement: Supplemental data [file jci-132-158661-s175.pdf]

Supplemental Methods

Mice

All experiments were performed under protocols (1108M, 1117M) approved by the Institutional Animal Care and Use Committee (IACUC) of the Roswell Park Comprehensive Cancer Center. All experiments were performed using female mice 8-12 weeks of age. BALB/c and C57BL/6 mice were obtained from Charles River Laboratories. IRF8-deficient mice (*Irf8*$^{-/-}$) on a C57BL/6 background were originally provided by Dr. K. Ozato (NIH, Bethesda, MD) and were backcrossed onto a BALB/c background *via* speed congenics at the Gene Targeting & Transgenic Shared Resource at Roswell Park in collaboration with Dr. Fernando Benavides (The University of Texas - M.D. Anderson Cancer Center), resulting in strain purity of ~99%. (See Supplemental Table 7 for a listing of all biological, chemical, and laboratory reagents/resources.)

Cell Lines and Tumor Growth Experiments

The 4T1 mammary tumor cell line was obtained from American Type Culture Collection (ATCC; Manassas, VA) and maintained, as described (46). The E0771.ML-1 mammary tumor cell line, a more aggressive variant of the parental E0771 cell line, was kindly provided by Dr. Vivek Mittal (Weill Medical College at Cornell University, New York, NY). Cell lines were confirmed to be mycoplasma-negative using the Mycoplasma PCR Primer Set (Agilent Technologies, Santa Clara, CA) and authenticated using the Mouse Cell STR Profiling Service provided by the ATCC. 4T1 and E0771.ML-1 cells were cultured in a RPMI-based culture media (containing 10% heat-inactivated FBS, 15 mM HEPES, 2 mM L-glutamine, 0.1 mM nonessential amino acids, 1 mM sodium pyruvate, 100 μg/mL penicillin-streptomycin Pen/Strep, and 50 μM 2-metcaptoethanol) at 37°C with 5% CO_2 and were removed from the flasks by trypsinization.

For the 4T1 tumor experiments, cells ($5x10^4$) were suspended in sterile DPBS and were implanted orthotopically into mammary gland no. 4 of syngeneic female BALB/c mice. For the E0771.ML-1 experiments, cells ($1x10^5$) were suspended in a Matrigel DPBS mixture (at a 1:1 ratio) and were similarly implanted orthotopically into mammary gland no. 4 of syngeneic female C57BL/6 mice. Tumor growth was measured 3 times per week, and the volumes were calculated using the formula $(w^2 \times l)/2$, where 'w' represents width and 'l' represents length.

Therapeutic Studies

Brequinar Sodium (either purchased from Tocris Bioscience, Bristol, UK, or received as a kind gift from Clear Creek Bio, Cambridge, MA, where indicated in the figure legends) was administered intraperitoneally (*ip*) in sterile H_2O at 10 mg/kg daily beginning when tumors first became measurable and was continued daily throughout the duration of the experiment. For the anti-PD-1 mAb experiments, anti-PD-1 mAb (BioXCell, Lebanon, NH) or the isotype control Ab (rat IgG2a BioXCell, Lebanon, NH) was injected *ip* at 200 μg/injection in sterile DPBS every other day for a total of 3 injections beginning 2 days after BRQ administration. On days when both BRQ/vehicle and anti-PD-1/isotype were administered, BRQ/vehicle was given in the morning and anti-PD-1/isotype was given in late afternoon no sooner than 6 hours following the previous injection on the adjacent side of the peritoneal cavity. For the anti-CTLA-4 mAb experiments, anti-CTLA-4 mAb (BioXCell) or the isotype control Ab (Syrian Hamster IgG Leinco, St. Louis, MO) was injected *ip* at 100 μg/injection in sterile DPBS every other day for a total of 3 injections beginning 2 days after BRQ administration.

For the uridine supplementation studies, uridine (Sigma-Aldrich, St. Louis, MO) was injected *ip* at 300 mg/kg daily concomitantly with BRQ for the duration of the experiment (78,

79). For the $CD8^+$ T cell depletion studies, anti-$CD8^+$ T cell-depleting mAb (BioXCell, Lebanon, NH) or the isotype (rat IgG2b BioXCell, Lebanon, NH in Figure 5C, or Leinco, St. Louis, MO in Figure 5D) was administered *ip* at 400 μg/injection in sterile DPBS. In Figure 5C, anti-CD8 mAb or isotype was given 3 days prior to tumor implantation, and then once a week for the duration of the experiment (80). While in Figure 5D, anti-CD8 mAb or isotype was given 7 days after tumor implantation and then once every 7 days for a total of 3 injections. $CD8^+$ T cell depletion in the peripheral blood was confirmed by flow cytometry 2 days following the administration of the mAb. On days when both BRQ/vehicle and anti-$CD8^+$ T cell/isotype Ab were administered, the former was administered in the morning and the latter was administered in late afternoon no sooner than 6 hours following the previous injection on the adjacent side of the peritoneal cavity.

Tissue Analysis

Primary tumor tissues were surgically removed (at the tumor volumes indicated in the specific figures or figure legends after euthanasia) and dissociated into a single cell suspension using the gentleMACS automatic tissue dissociator system (Miltenyi Biotec, North Rhine, Germany) and a collagenase/hyaluronidase cocktail (StemCell Technologies, Vancouver, CA), as per manufacturer's instructions. Dissociation occurred in a Hybaid rotating incubator (Phoenix Equipment) for 1 hour at 37°C. The resulting mixture was then strained through 100 μm SureStrain cell strainers (MTC Bio, Sayreville, NJ). Spleens were mechanically dissociated and then passed through a 100 μm SureStrain cell strainer. BM cells were collected by flushing or short centrifugation of tibias and femurs. For all tissues, RBCs were lysed using Ammonium-Chloride-Potassium (ACK) lysis buffer, and the cells were then used for further analysis, as described in the appropriate assays. For quantification of lung metastatic nodules, lungs were

isolated following euthanasia, washed in DPBS and fixed in 10% formalin. Slides were prepared from formalin-fixed paraffin-embedded blocks, followed by histologic staining with hematoxylin and eosin, and quantification of the metastatic nodules was done under a light microscope in a blinded manner.

Peripheral Blood Analysis

Peripheral blood was obtained *via* retro-orbital blood collection. Mice were placed under general anesthesia utilizing inhaled isoflurane. Blood was collected using heparinized capillary tubes (Chase Scientific, Lanley, WA) that flowed into collection tubes containing EDTA to a maximum volume of 200 μl. RBCs were removed using ACK lysis buffer and cells were analyzed by flow cytometry.

Flow Cytometry

Analysis of splenic/in vitro-derived MDSCs

Cells ($1x10^5$ – $1x10^6$) were immunostained with the appropriate mAbs for either 20 minutes at room temperature or 1 hour at 4 – 8°C in flow-based buffer (DPBS with 5% heat-inactivated FBS or 0.5% BSA) and washed once with the same buffer. Cells were fixed/permeabilized overnight (eBioscience, Santa Clara, CA, FoxP3/Transcription Factor Staining Buffer Kit) for intracellular staining of VEGF-A and iNOS. Cells were then incubated with the mAb at 4°C for 45 minutes or overnight. In all cases, cells were washed and fixed overnight in 2% paraformaldehyde at room temperature. The next day, cells were washed and resuspended in flow-based buffer and analyzed by flow cytometry. Samples were analyzed on a LSR Fortessa cytometer (BD Biosciences, Mississauga, ON) running FACSDiva version 6.1.3.

Doublets were excluded, based on forward and side scatter. Live cells were identified as LIVE/DEAD$^{lo/-}$. Data files were analyzed using FlowJo version 10. Gating strategies for the identification and quantification of mouse or human MDSC/myeloid subsets are included in **Supplementary Figure 2 and Figure 8**. Data are reported as either the percentage of the parent gate, percentage of live single cells, or absolute cell number.

Analysis of MDSC apoptosis

MDSCs ($5x10^5$) were first stained with surface Abs in flow-based buffer, as described above. Cells were then washed in Annexin-V binding buffer and stained for both DAPI and Annexin-V for 15 minutes at room temperature, following the manufacturer's protocols. Heat-shocked cells were used as a positive control.

Analysis of the tumor microenvironment

Tumors were enzymatically and mechanically dissociated, as described earlier. Afterwards, cells ($1x10^6$) were incubated with the appropriate Abs for 15 minutes at room temperature in a modified flow-based buffer (containing 2% heat-inactivated FBS and 2 mM EDTA). Cells were then washed in the same buffer and fixed/permeabilized overnight as described above for intracellular staining of Ki-67. Gating strategies for the identification and quantification of $CD45^+$ leukocytes, MDSC subsets, macrophages, and $CD8^+$ T cells can be found in Supplemental Figure 6. Cells/g Tumor were calculated as *Tumor Weight (g)/((% Live Cells/100) x Cell Count).* Additionally, MDSCs were collected from individual tumors of Veh- or BRQ-treated tumor-bearing mice using the EasySep^{TM} Mouse MDSC isolation kit (STEMCELL Technologies, Cambridge, MA), enriching for $CD11b^+Gr\text{-}1^+$ cells, per manufacturer's instructions, followed by RNA extraction and RT-qPCR analyses, as described below.

Analysis of bone marrow progenitors

BM cells from two hind limbs (femurs and tibias) per individual mouse were isolated using centrifugation and resuspended in 1 ml flow-based buffer (81). To lyse red blood cells, cells were suspended in 5 ml of ACK lysis buffer and incubated for 10 minutes on ice. Unfractionated BM cells (5×10^6) were resuspended in flow-based buffer and stained with rat anti-mouse Flt3 mAb for 10 minutes at room temperature. Cells were then washed, resuspended in Brilliant Stain buffer (BD Biosciences, Mississauga, ON) and stained with the indicated primary mAbs and LIVE/DEAD Fixable Blue Dead Cell Stain (Thermo Fisher Scientific, Waltham, MA) for 30 minutes on ice. Cells were washed and stained with streptavidin-BUV661 for an additional 30 minutes on ice, followed by analysis using an Aurora full spectrum flow cytometer (Cytek Biosciences, Fremont, CA). Doublets were excluded based on forward and side scatter. Live cells were identified as LIVE/DEAD$^{lo/-}$. Gating strategies for the identification of mature myeloid cells and hematopoietic stem and progenitor cells (HSPCs) are shown in Supplemental Figure 7 and are based on previously described immunophenotypes (18, 41, 81-86). Data were analyzed using FCS Express v. 7.06.0015 software (De Novo Software LLC, Pasadena, CA). Mature myeloid cell and HSPC populations are reported as percentages of live, single events.

Cell Sorting

For the experiments involving the isolation of MDSCs from in vitro cultures or freshly collected splenocytes, we used the Miltenyi Mouse Myeloid-Derived Suppressor Cell Isolation Kit (Cat # 130-094-538) following the manufacturer's protocol that enriched for the Gr-1^{high} Ly6G^{+} cells. Additionally, the EasySep^{TM} Mouse MDSC isolation kit (STEMCELL

Technologies, Cambridge, MA) was used for the isolation of MDSCs from splenocytes for some experiments in Figure 3 and for the isolation of MDSCs from tumors in Figure 6 and Supplemental Figure 8, see figure legends. For experiments generating MDSCs from GMPs, lineage-depletion of BM was performed by staining cells using anti-mouse mAbs reactive against Ter-119, Gr-1, B220, and CD3 (eBioscience). Stained Lin^+ cells were magnetically sorted and removed using BioMag goat anti-mouse/anti-rat IgG beads (Qiagen, Hilden, Germany). Lineage-negative (Lin^-) BM cells were stained with anti-mouse mAbs against c-Kit, Sca-1, CD16/32, and CD150 to identify GMPs (84). Cells were sorted using the FACS Aria II cell sorter running FACSDiva acquisition software. Gating strategies used to identify the various BM progenitors, including GMPs are shown in Supplemental Figure 7. For the experiments involving purified $CD11b^+Gr\text{-}1^+$ cells, splenocytes were collected and stained with anti-mouse mAbs reactive against CD11b and Gr-1. $CD11b^+Gr\text{-}1^+$ cells were sorted using the SONY MA900 Multi-Application Cell Sorter.

In Vitro Murine MDSC Differentiation

Unfractionated BM cells ($3x10^6$) were plated in complete RPMI media containing recombinant murine GM-CSF and G-CSF (Peprotech, East Windsor, NJ) at 40 ng/mL. At the start of the culture, either BRQ (TOCRIS or Clear Creek Bio as indicated in the results) at 0.5-2 μM or leflunomide (Lef; Sigma-Aldrich, St. Louis, MO) at 25 μM were added. Cells were incubated at 37°C for 4 days after which cells were subjected to vigorous pipetting to collect all non-adherent cells. For studies in which MDSCs were generated *ex vivo* from GMPs, GMPs were sorted from 4T1-bearing mice treated with either BRQ or vehicle control as described under "Cell Sorting". GMPs ($1x 10^6$) were plated in complete RPMI containing recombinant

murine G-CSF at 40 ng/ml for 4 days (18). After culture, cells were subjected to vigorous pipetting to collect all non-adherent cells and analyzed by flow cytometry or used in T cell proliferation assays as described below.

In Vitro Human Bone Marrow Culture System

De-identified human bone marrow (hBM) cells were obtained during the collection of products for use in BM transplant from healthy male or female donors. Human BM use was approved by an Internal Review Board (IRB) approved protocol (BDR 134520) at the Roswell Park Comprehensive Cancer Center. Human BM mononuclear cells were isolated by Ficoll-Hypaque density gradient centrifugation (GE, Boston, MA). Cells were spun over the gradient for 30 min at 1300 RPM. Cells remaining within the interface were collected by Pasteur pipette. Unfractionated BM cells ($3x10^6$) were cultured in complete RPMI media containing human recombinant GM-CSF and G-CSF (Peprotech) at 40 ng/ml each $\pm$ BRQ (Clear Creek Bio) at 1 μM for 4 days. Cells were then subjected to vigorous pipetting to collect all non-adherent cells and analyzed by flow cytometry or RT-qPCR, as described below.

T Cell Proliferation Assay

In vivo- or in vitro-derived MDSCs were co-incubated with splenocytes as a source of T cells, which had been pre-stained with CellTrace Violet (Thermo Fisher Scientific, Waltham, MA) per the manufacturer's instructions, at a 1:1 ratio for 72 hours in 96-well round-bottomed plates. Cultures were stimulated with or without agonistic anti-CD3 mAb (1 μg/ml). Dilution of CellTrace Violet in both $CD4^+$ and $CD8^+$ T cells was then analyzed by flow cytometry, as described (18). The percentage of proliferation was calculated as (ΔMFI anti-CD3–stimulated T

cells minus ΔMFI cocultured T cells) divided by ΔMFI anti-CD3-stimulated T cells, where the difference in mean fluorescence intensity (ΔMFI) was calculated by subtraction from the unstimulated controls.

Morphology Studies

In vitro-generated MDSCs were harvested as described earlier and suspended in DPBS at a concentration of $1x10^6$ cells/ml. Then 100 μl of cell suspension (1×10^5 cells) was cytocentrifuged onto Shandon Cytoslides (Thermo Fisher Scientific, Waltham, MA). Slides were air-dried for at least 10 minutes before staining with Wright-Giemsa stain. Morphologic quantification for the proportions of segmented neutrophils, macrophages, and immature cells was performed by Dr. Vishala Neppalli, a heme pathologist, in a blinded manner.

RT-qPCR

Total RNA was isolated using the RNeasy Mini Kit (Qiagen). cDNA was synthesized using the iScript cDNA synthesis kit (Bio-Rad). The cDNA was used for PCR amplification using specific primer sets. RT-qPCR was performed using CFX Maestro 1.1 (Bio-Rad). SYBR-Green (Invitrogen) was used as the dye for quantification. Data were quantified using the formula-fold change=$2^{-\Delta\Delta CT}$. All results were reported as a ratio of the specific mRNA signal normalized to the indicated housekeeping gene.

Arginase Activity Assay

A BCA assay (ThermoFisher) was used to quantify the protein concentrations of in vitro-generated MDSCs after lysis with a Tris-EDTA based buffer (10 mM Tris-HCl, 0.4% Triton X-

100, and HALT Protease Cocktail). The arginase activity assay (Sigma-Aldrich) was performed following the manufacturer's recommended protocol with 20 μg of cell lysate for each sample. A urea standard curve was used to convert the amount of urea produced into mM.

Single-cell RNA-seq Studies

Single cell libraries were generated using the 10X Genomics platform and Chromium Next GEM Single Cell 3' Reagent Kits v3.1 with Feature Barcode technology for Cell Surface Protein. First, Feature Barcode conjugated molecules were bound to cell surface proteins. Cell suspensions were assessed with Trypan Blue using a Countess FL automated cell counter (ThermoFisher) to determine concentration, viability and the absence of clumps and debris that could interfere with single cell capture. The cells were then loaded into the Chromium Controller (10X Genomics) where they are partitioned into nanoliter-scale Gel Beads-in-emulsion with a single barcode per cell. Reverse transcription was performed, and the resulting cDNA was amplified. Amplified cDNA was separated into full-length cDNA and Feature Barcode fractions using SPRISelect beads (Beckman Coulter). The full-length amplified cDNA was used to generate libraries by enzymatic fragmentation, end-repair, a-tailing, adapter ligation, and PCR to add Illumina compatible sequencing adapters. Feature barcode derived cDNA was PCR amplified to incorporate Illumina adapter sequences and unique sample indexes. The resulting libraries were evaluated on D1000 screentape using a TapeStation 4200 (Agilent Technologies) and quantitated using Kapa Biosystems qPCR quantitation kit for Illumina. Final libraries were then pooled, denatured, and diluted to 300 pM with 1% PhiX control library added. The resulting pool was loaded into the appropriate NovaSeq Reagent cartridge and sequenced on a NovaSeq6000 following the manufacturer's recommended protocol (Illumina Inc.).

The raw sequencing data from the Chromium 10x Genomics libraries were processed using Cellranger version 6.0.0 software. Then, the filtered gene-barcode matrices which contain barcodes with the Unique Molecular Identifier (UMI) counts that passed the cell detection algorithm were used for further analysis with Seurat single cell data analysis R package (87). First, the cells were demultiplexed based on HTO (hash tag oligos) and only singlet cells were kept for further analysis. Then cells with high mitochondrial RNA contents (>15%), low RNA contents (<500 transcripts) or high RNA contents (> 7500 transcripts) were removed. Additionally, the cell cycle assignment was performed by using CellCycl Scoring function of the Seurat package. Lists of cell cycle genes from Buettner, *et al*., were used to calculate S score and G2/M score, respectively, for each cell (88). The score is the average expression of the cell cycle genes subtracted by the aggregated expression of control feature sets, which has the same number of genes and similar average gene expression values as cell cycle genes. Then, the normalized and scaled UMI counts were calculated using the SCTransform method and regressed against the cell cycle scores. Dimension reductions including principal component analysis (PCA), UMAP and tSNE using the highly variable genes were performed. Cells are clustered using the shared nearest neighbor (SNN)-based method. The cells were annotated using SingleR package with ImmGen reference database (89). To study GMPs, cells with scaled Ms4a3 expression value greater than 3 (primary GMP cells) were selected to identify genes related to the treatment using MAST (90). Pathway analysis was performed on GMP and GN populations with the Gene Set Enrichment Analysis (GSEA) in pre-ranked mode with gene list ranked with average log2 fold changes for each comparison (91). Pathway analysis was run against MSigDB, a collection of annotated and curated gene set repositories provided by the developer of GSEA (Broad Institute MIT and Harvard). This particular run used C2 of version

7.4 collection, containing 2307 gene sets from various well-known and up-to-date pathway databases such as BioCarta, KEGG and Reactome, among others. Pseudotime and single cell trajectory analysis were done with Monocle 3 (92). The Seurat single cell object was converted to Monocle cell data set to keep the graphical representation consistent. The pseudotime and cell trajectory were generated using cells with maximum *Mecom* gene expression as the root. Cells are ordered by computed pseudotime and the trajectory graph was plotted to show the trajectory of different populations of cells. All single-cell RNA-seq data from this BM analysis are deposited under GSE190232.

Statistics

Data were analyzed using GraphPad Prism (v9.3.1) for all *t*-tests. Data were recorded as the mean ± SEM for the indicated number of mice or biologic or experimental replicates. Differences in metastatic outcome between treatment groups were determined by a 2-tailed unpaired *t*-test. Differences in cell populations as assessed by flow cytometry or mRNA expression levels for the indicated genes were determined by a 2-tailed unpaired *t*-test, as indicated, or in the case of the human donors, a 2-tailed paired *t*-test. Statistical analyses involving multiple comparisons for experiments with more than two groups were performed. When comparing multiple groups, the Holm-Bonferroni correction method was applied to the primary comparisons to assess a change in efficacy. In all cases, p-values < 0.05 were considered statistically significant. Differences in primary tumor growth over time between groups were determined by the two-sided Wald test. Tumor growth is assumed to be approximately linear within the time of measurements. The linear mixed model (LMM) was used to model the growth curves to account for the within-subject correlations. In this model, the group-by-time interaction

effect corresponds to the difference in growth rate compared to the reference group. The synergistic effect between two treatments were examined by a three-way interaction between the treatments with time. Differences between two groups were examined by appropriate contrasts. The restricted maximum likelihood method was used for model fitting. Statistical significance was obtained using the two-sided Wald test. A p-value < 0.05 was considered statistically significant. The methods for LMM were implemented using lme4 and lmerTest packages under R 4.1.2.

Supplemental Figure 1

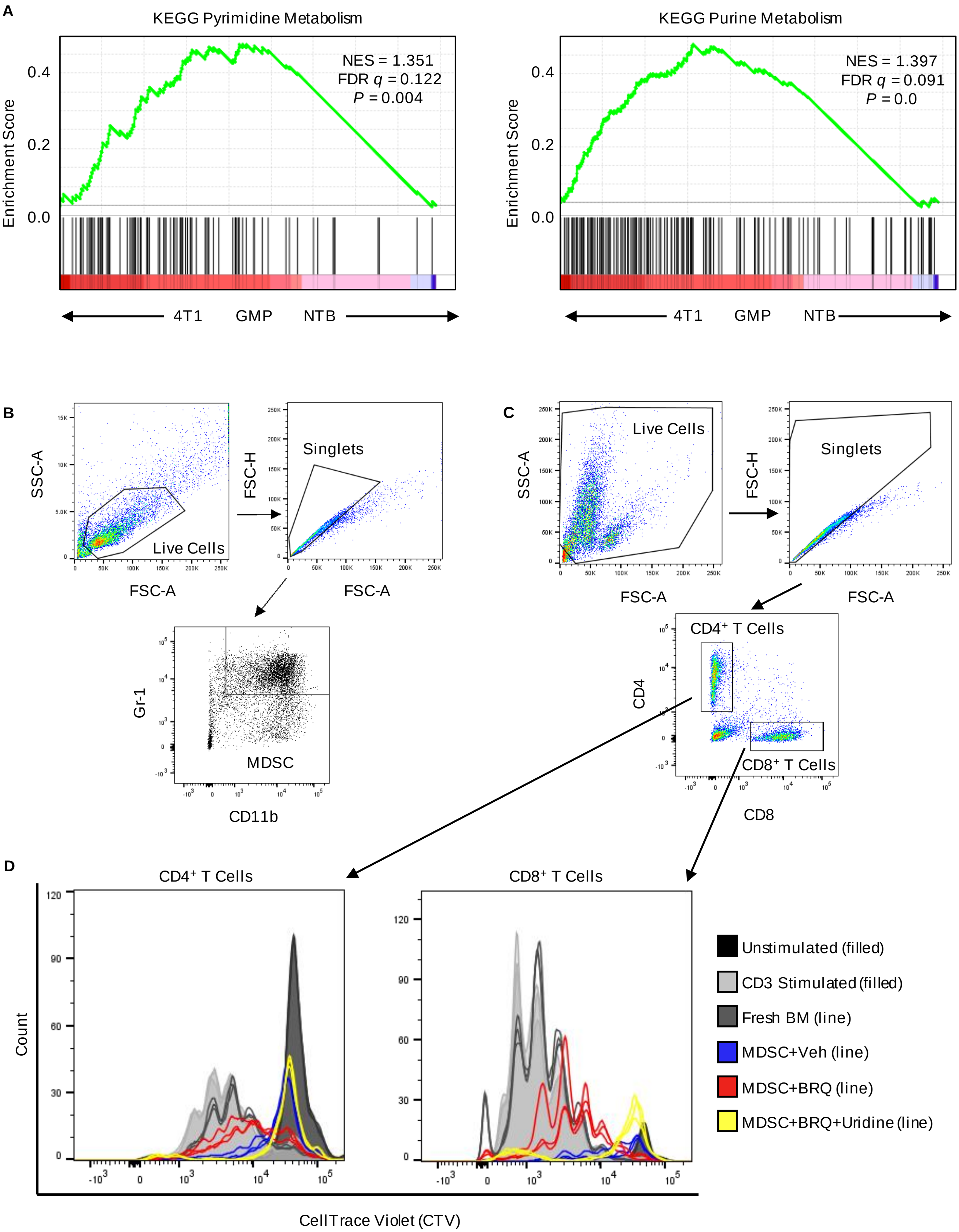

Supplemental Figure 1. Related to Figure 1. (***A***) Gene Set Enrichment Analysis (GSEA) of the pyrimidine (*left*) and purine (*right*) pathways expressed in GMPs purified in 4T1-bearing or non-tumor bearing (NTB) mice. RNA-seq data were analyzed under accession number GSE193263. Plots show the normalized enrichment score (NES) for pathway-associated genes, FDR *q* values, and nominal p-values. (***B***) Gating strategies for in vitro-generated MDSCs following co-culture experiments (see **Figure 1B**). (***C***) Gating strategies for T cell subsets following the co-culture experiments with MDSCs (see **Figure 1D and 1E**). (***D***) CTV histograms depicting T cell proliferation following the indicated co-culture conditions (see **Figure 1D and 1E**).

Supplemental Figure 2

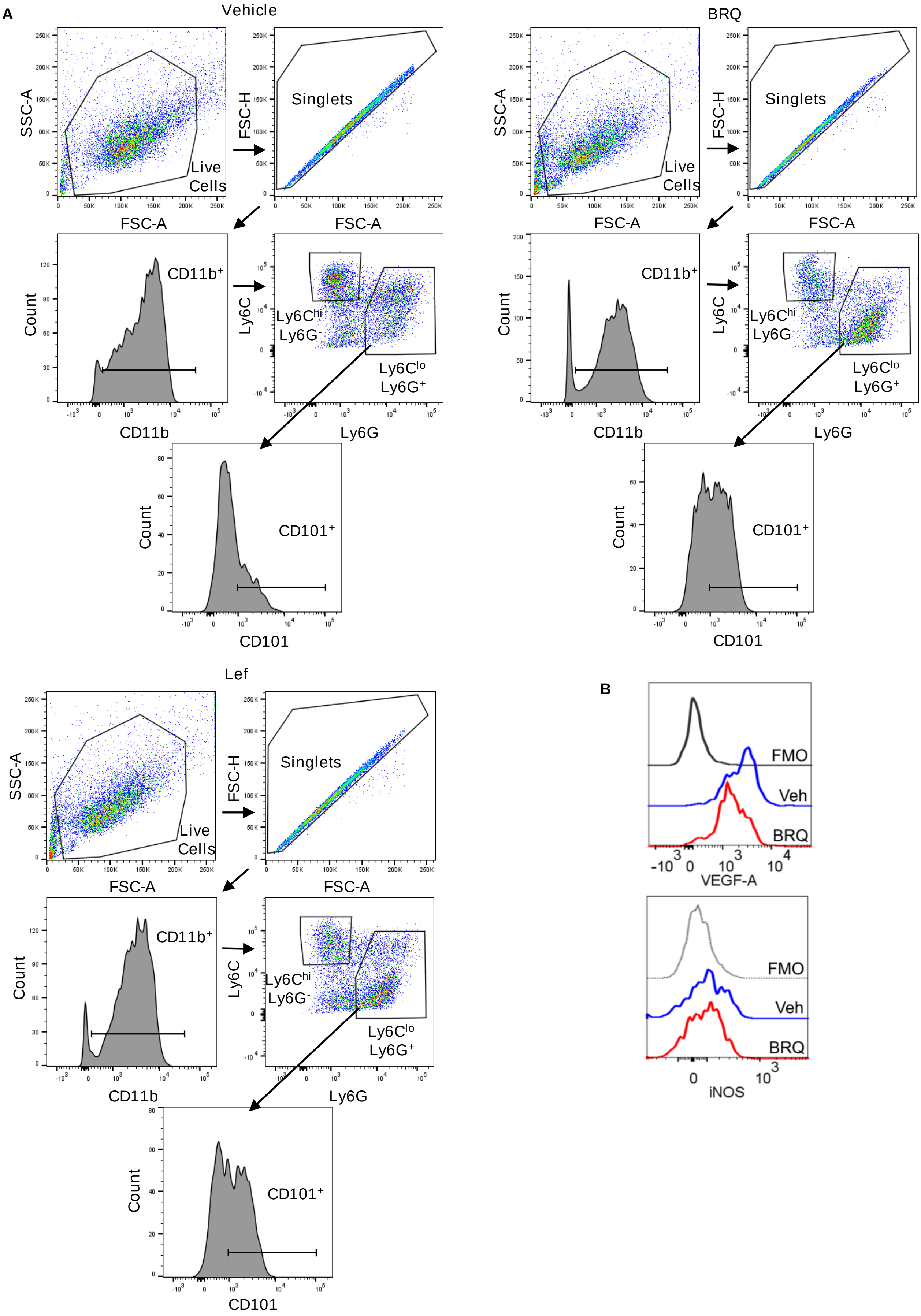

Supplemental Figure 2. Related to Figure 2. (***A***) Gating strategy used to identify the $CD11b^{+}Ly6C^{lo}Ly6G^{+}$ or $CD11b^{+}Ly6C^{hi}Ly6G^{-}$ subsets for CD101 expression (see **Figure 2B**). (***B***) Representative flow cytometry histograms of VEGF-A and iNOS of in vitro-derived PMN-MDSCs (see **Figure 2E**).

Supplemental Figure 3

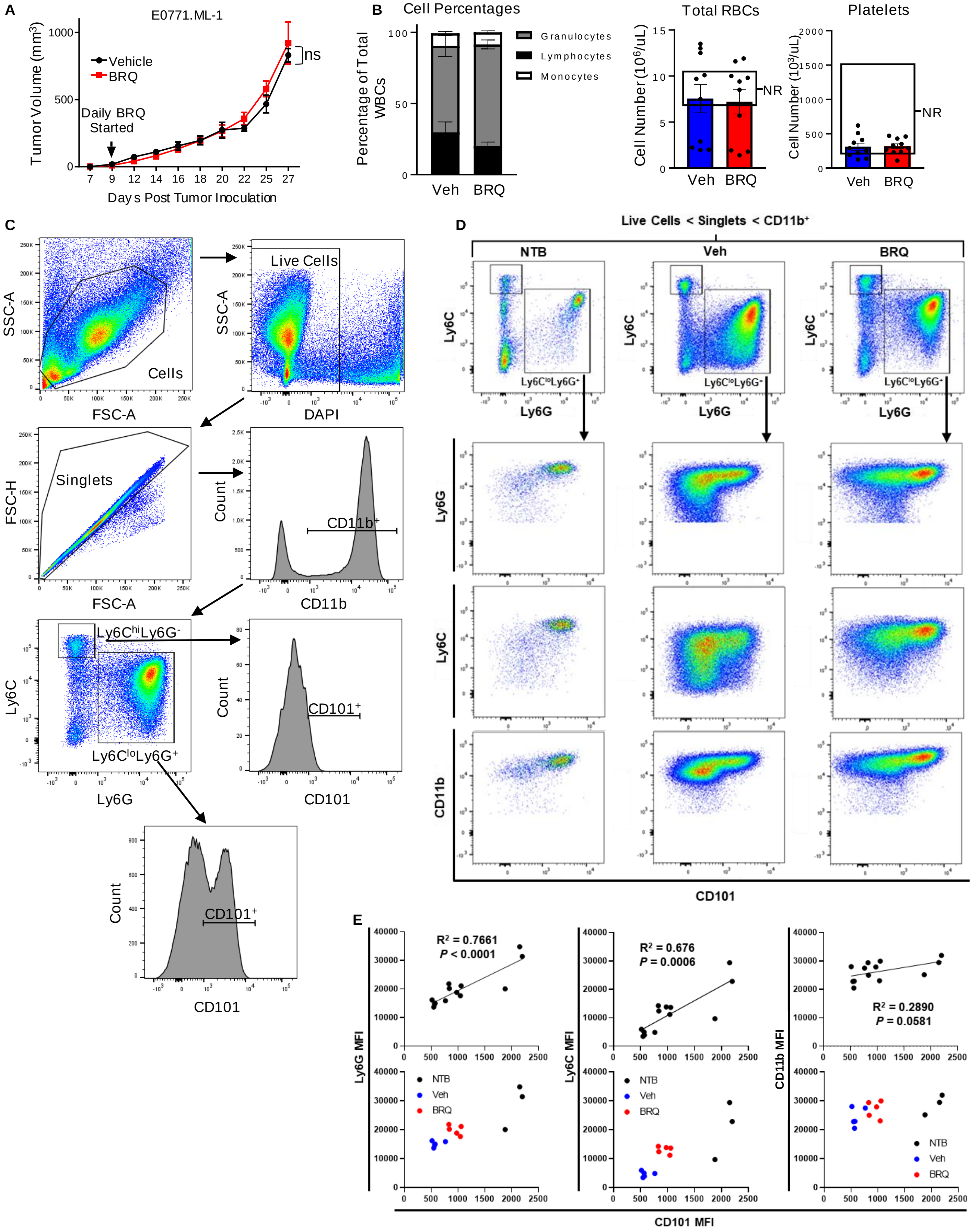

Supplemental Figure 3. Related to Figure 3. (***A***) E0771.ML-1 tumor growth in female C57BL/6 mice treated with daily administration of BRQ as in **Figure 3A**. (***B***) Percentages and cell numbers of indicated cell populations from the peripheral blood of 4T1-bearing mice treated with Veh or BRQ (see **Figure 3D**). Boxed areas indicate normal range (NR). (***C***) Gating strategy used to quantify CD101 expression on the MDSC subsets (see **Figure 3E**). (***D***) Flow cytometry of the various combinations of Ly6C, Ly6G, CD11b and CD101 expression of representative NTB, Veh- or BRQ-treated mice. (***E***) Linear regression of CD101 *versus* Ly6C, Ly6G, or CD11b expression (n=3-5 mice/group). Data are recorded as the mean ± SEM of the indicated data points.

Supplemental Figure 4

A

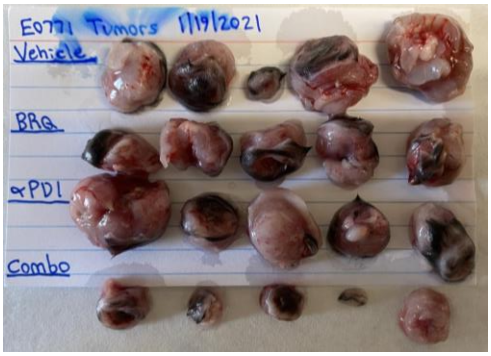

B

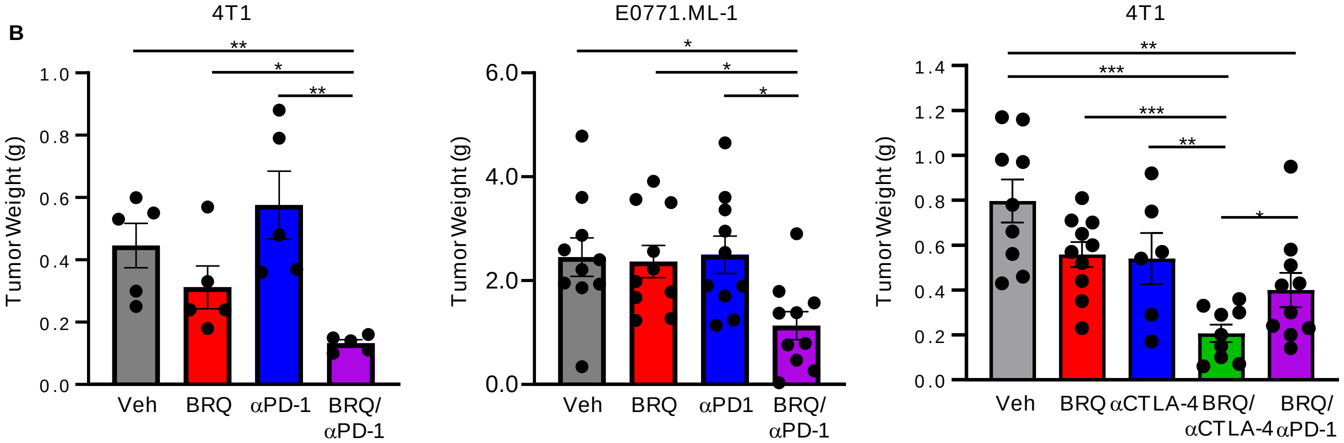

Supplemental Figure 4. Related to Figure 4. (***A***) Photograph of endpoint E0771.ML-1 tumors from the different treatment groups. (***B***) Tumor weights from 4T1 (*left and right*) or E0771.ML-1 (*middle*) treatment groups from **Figure 4**. Data are recorded as the mean ± SEM of indicated data points and represent n=5-10 mice/group. Unpaired *t*-test: ns = not significant; * = $p < 0.05$, ** = $p < 0.01$, *** = $p < 0.001$.

Supplemental Figure 5

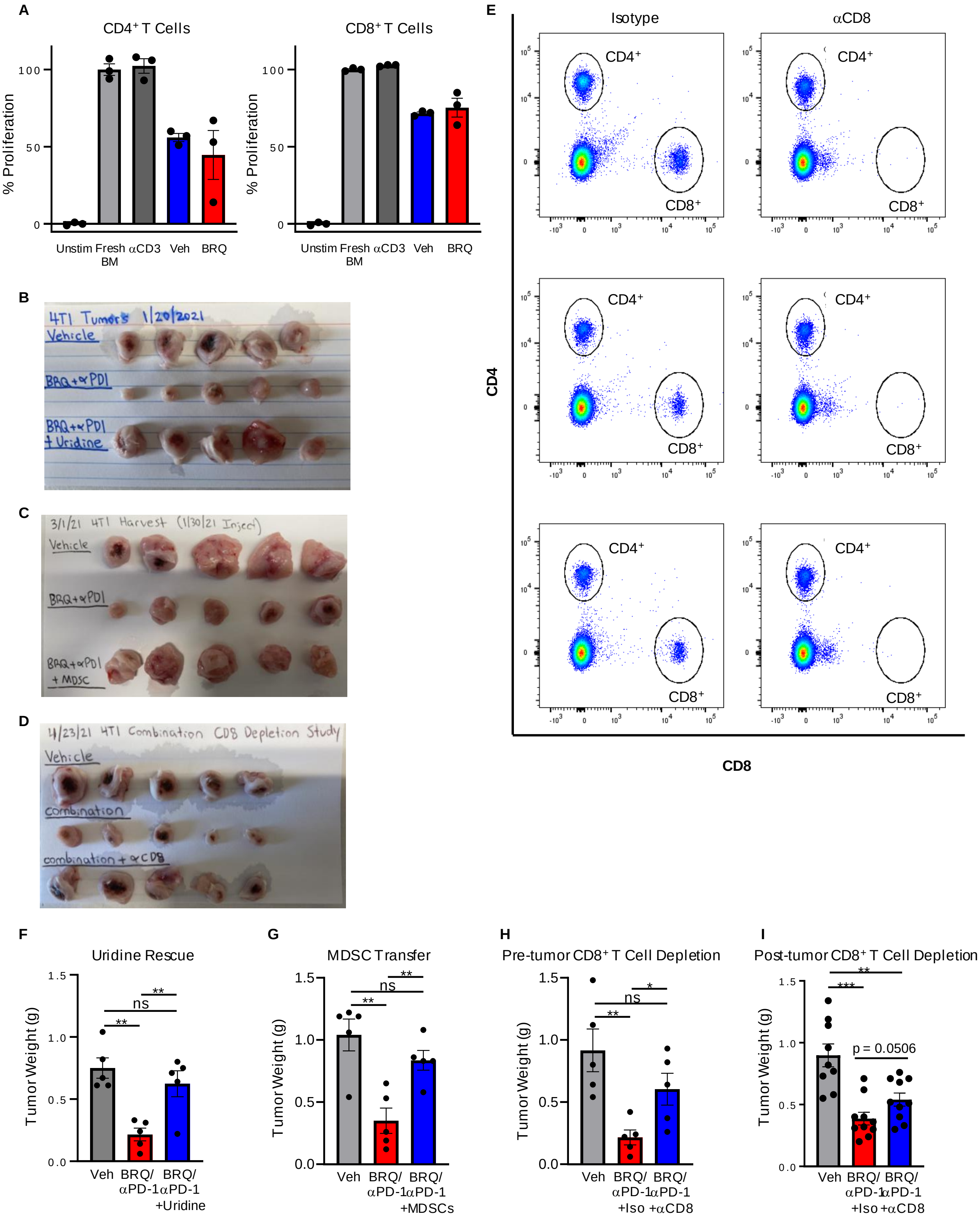

Supplemental Figure 5. Related to Figure 5. (***A)*** *Irf8*$^{-/-}$ BM cells were cultured with cytokines ± BRQ, as in **Figure 1**. After culture, the myeloid cells were assayed for their ability to inhibit $CD4^{+}$ or $CD8^{+}$ T cell proliferation. Data are recorded as the mean ± SEM of triplicate determination and are representative of two independent experiments. (***B*)** Photographs of endpoint 4T1 tumors collected from mice treated with vehicle, BRQ + αPD-1, or BRQ + αPD-1 + uridine (see **Figure 5A**). (***C*)** Photographs of endpoint 4T1 tumors collected from mice treated with vehicle, BRQ + αPD-1, or BRQ + αPD-1 + MDSCs (see **Figure 5B**). (***D***) Photographs of endpoint 4T1 tumors harvested from mice treated with vehicle, BRQ + αPD-1, or BRQ + αPD-1 + αCD8-depleting antibody (see **Figure 5C)**. (***E***) Flow dot plots of CD4/CD8 expression in peripheral blood collected 2 days following first treatment with the isotype/αCD8 antibody as in Figure 5C. (***F-I***) 4T1 tumor weights at endpoint corresponding to Figure 5. (***F-I***) Data are recorded as the mean ± SEM of multiple determinations (n=5-10 mice/group). Unpaired *t*-test: ns = not significant; * = $p < 0.05$, ** = $p < 0.01$, *** = $p < 0.001$.

Supplemental Figure 6

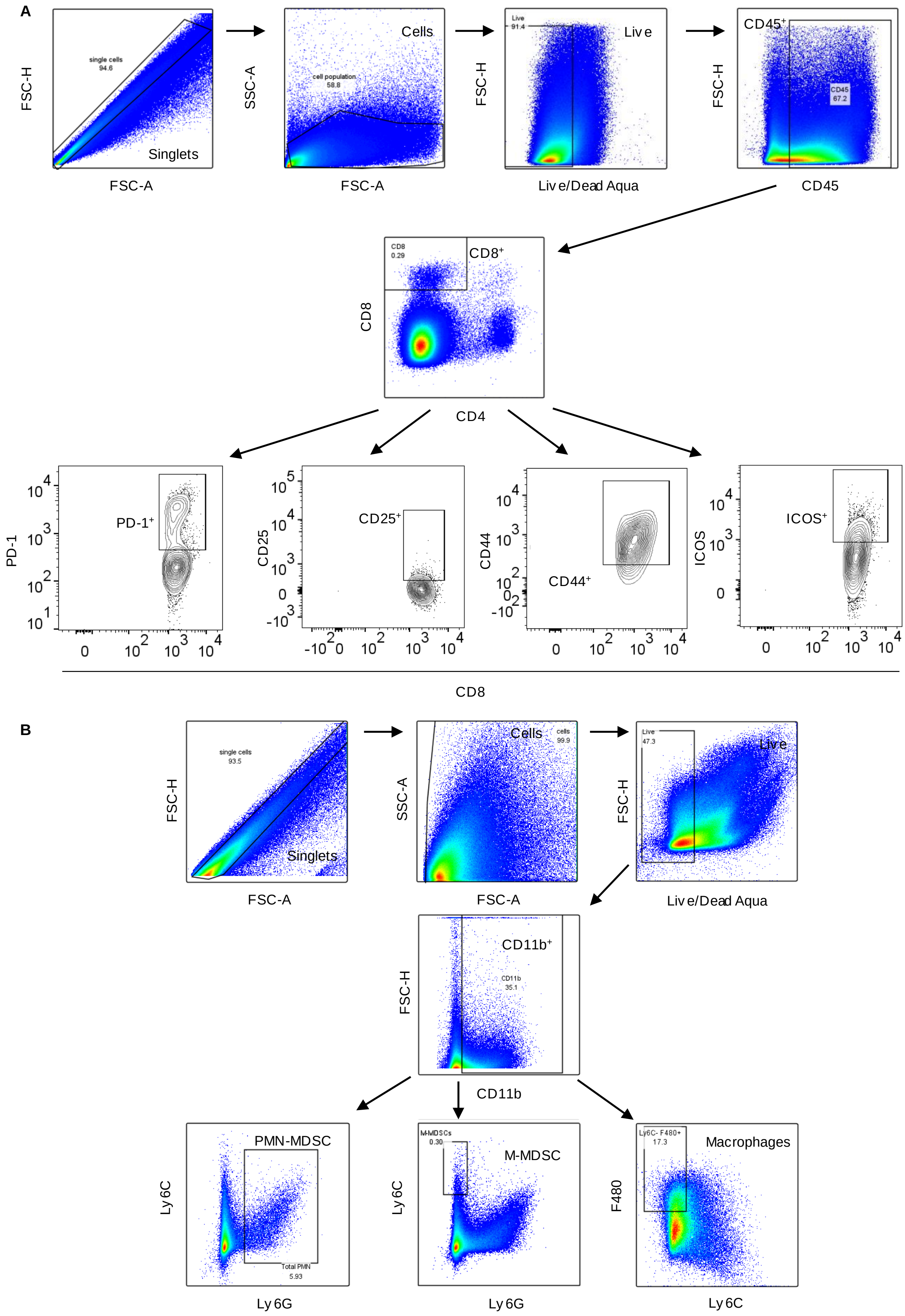

Supplemental Figure 6. Related to Figure 6. (***A***) Flow gating strategy used to identify CD8^{+} T cells, and their expression of PD-1, CD25, CD44, and ICOS in 4T1 tumors collected at endpoint for **Figure 6**. (***B***) Flow gating strategy to identify PMN-MDSCs, M-MDSCs, and macrophage populations from the 4T1 tumors collected at endpoint.

Supplemental Figure 7

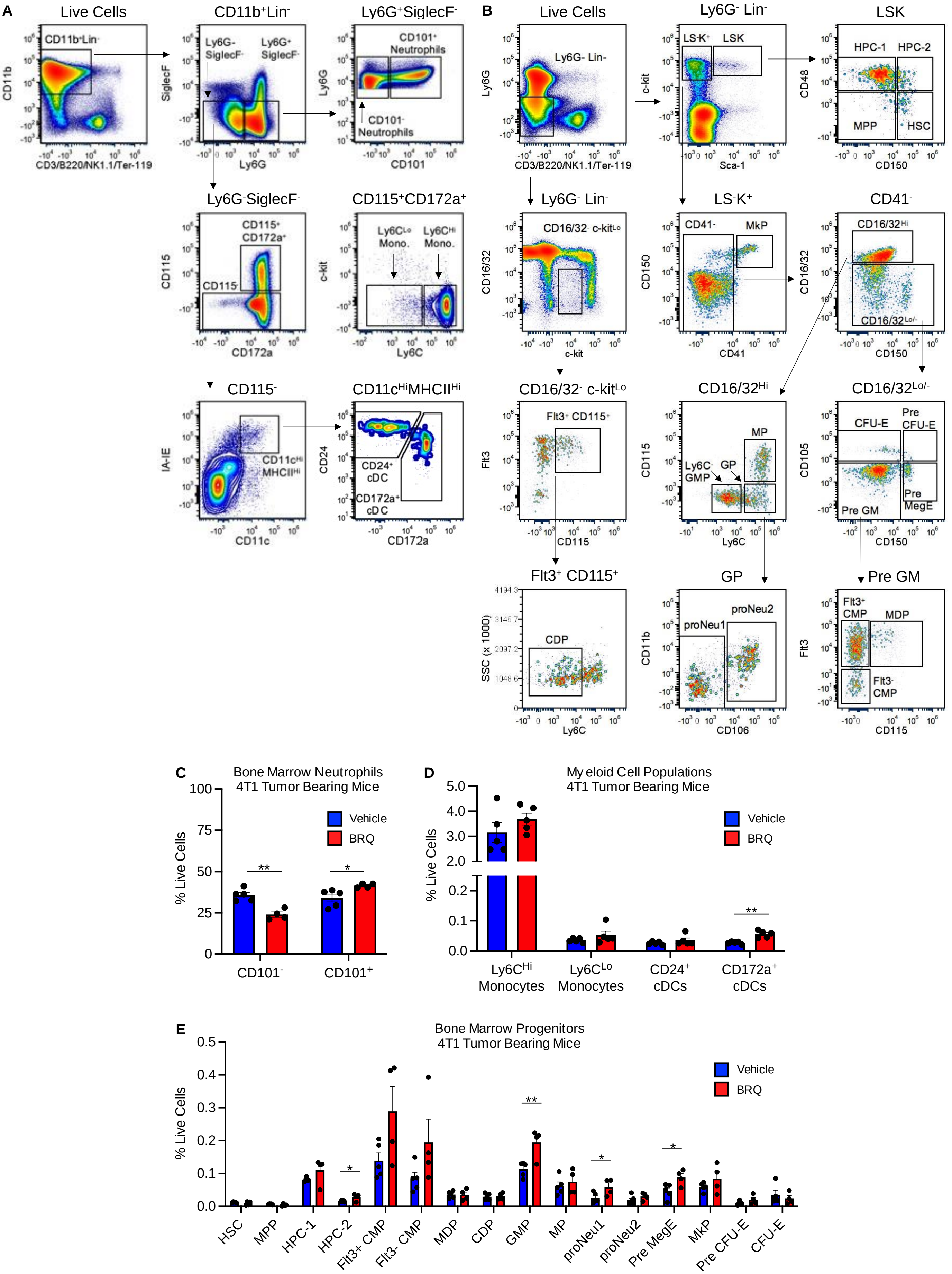

Supplemental Figure 7. Related to Figure 7. (***A***) Flow gating strategy to detect neutrophil, monocyte and dendritic cell (DC) populations in the BM of 4T1-bearing mice. (***B***) Flow gating strategy to detect hematopoietic stem and progenitor (HSPC) populations in the BM of 4T1-bearing mice. Lin^-: Lineage (CD3/B220/NK1.1/Ter-119)-Negative; LSK: $Lin^-Sca\text{-}1^+c\text{-}kit^+$; LS^-K^+: $Lin^-Sca\text{-}1^-c\text{-}kit^+$; Abbreviations for specific HSPC populations reported were based on the following immunophenotypes:

HSC (Hematopoietic stem cell): LSK, $CD150^+CD48^-$

MPP (Multipotent Progenitor): LSK, $CD150^-CD48^-$

HPC-1 (Hematopoietic Progenitor-1): LSK, $CD150^-CD48^+$

HPC-2 (Hematopoietic Progenitor-2): LSK, $CD150^+CD48^+$

$Flt3^+$ CMP ($Flt3^+$ Common Myeloid Progenitor): LS^-K^+, $CD41^-CD16/32^{Lo}CD150^-CD105^-Flt3^+CD115^-$

$Flt3^-$ CMP ($Flt3^-$ Common Myeloid Progenitor): LS^-K^+, $CD41^-CD16/32^{Lo}CD150^-CD105^-Flt3^-CD115^-$

MDP (Monocyte-Dendritic Cell Progenitor): LS^-K^+, $CD41^-CD16/32^{Lo}CD150^-CD105^-Flt3^+CD115^+$

CDP (Common Dendritic Cell Progenitor): $Lin^-c\text{-}kit^{Lo}CD16/32^-CD150^-Flt3^+CD115^+Ly6C^-$

GMP (Granulocyte-Monocyte Progenitor): LS^-K^+, $CD41^-CD16/32^{Hi}CD150^-Ly6C^-CD115^-$

MP (Monocyte Progenitor): LS^-K^+, $CD41^-CD16/32^{Hi}CD150^-Ly6C^+CD115^+$

proNeu1 (proNeutrophil 1): LS^-K^+, $CD41^-CD16/32^{Hi}CD150^-Ly6C^+CD115^-CD11b^-CD106^-$

proNeu2 (proNeutrophil 2): LS^-K^+, $CD41^-CD16/32^{Hi}CD150^-Ly6C^+CD115^-CD11b^{Lo}CD106^+$

Pre MegE (Pre Megakaryocyte-Erythroid Progenitor): LS^-K^+, $CD41^-CD16/32^{Lo}CD150^+CD105^-$

MkP (Megakaryocyte Progenitor): LS^-K^+, $CD41^+CD150^+$

Pre CFU-E (Pre Colony-Forming Unit, Erythroid): LS^-K^+, $CD41^-CD16/32^{Lo}CD150^+CD105^+$

CFU-E (Colony-Forming Unit, Erythroid): LS^-K^+, $CD41^-CD16/32^{Lo}CD150^-CD105^+$

(***C***) Frequency of $CD101^-$ and $CD101^+$ neutrophils ($Lin^-CD11b^+Siglec\text{-}H^-Ly6G^+$) in the BM of 4T1-bearing mice treated with vehicle or BRQ (***D***) Frequency of monocyte and DC subsets in the BM of 4T1-bearing mice treated with vehicle or BRQ. (***E***) Frequency of hematopoietic stem and progenitor cell populations in the BM of 4T1-bearing mice treated with Veh or BRQ. In (***C*** – ***E***)**,** Vehicle (n = 5) and BRQ (n = 4). An outlier was removed from the BRQ group using ROUT analysis (GraphPad Prism v9.1.2). Data are recorded as the mean ± SEM (shown as individual data points). Unpaired *t*-test (*C*-*E*): * = $p < 0.05$, ** = $p < 0.01$, **** = $p < 0.001$.

Supplemental Figure 8

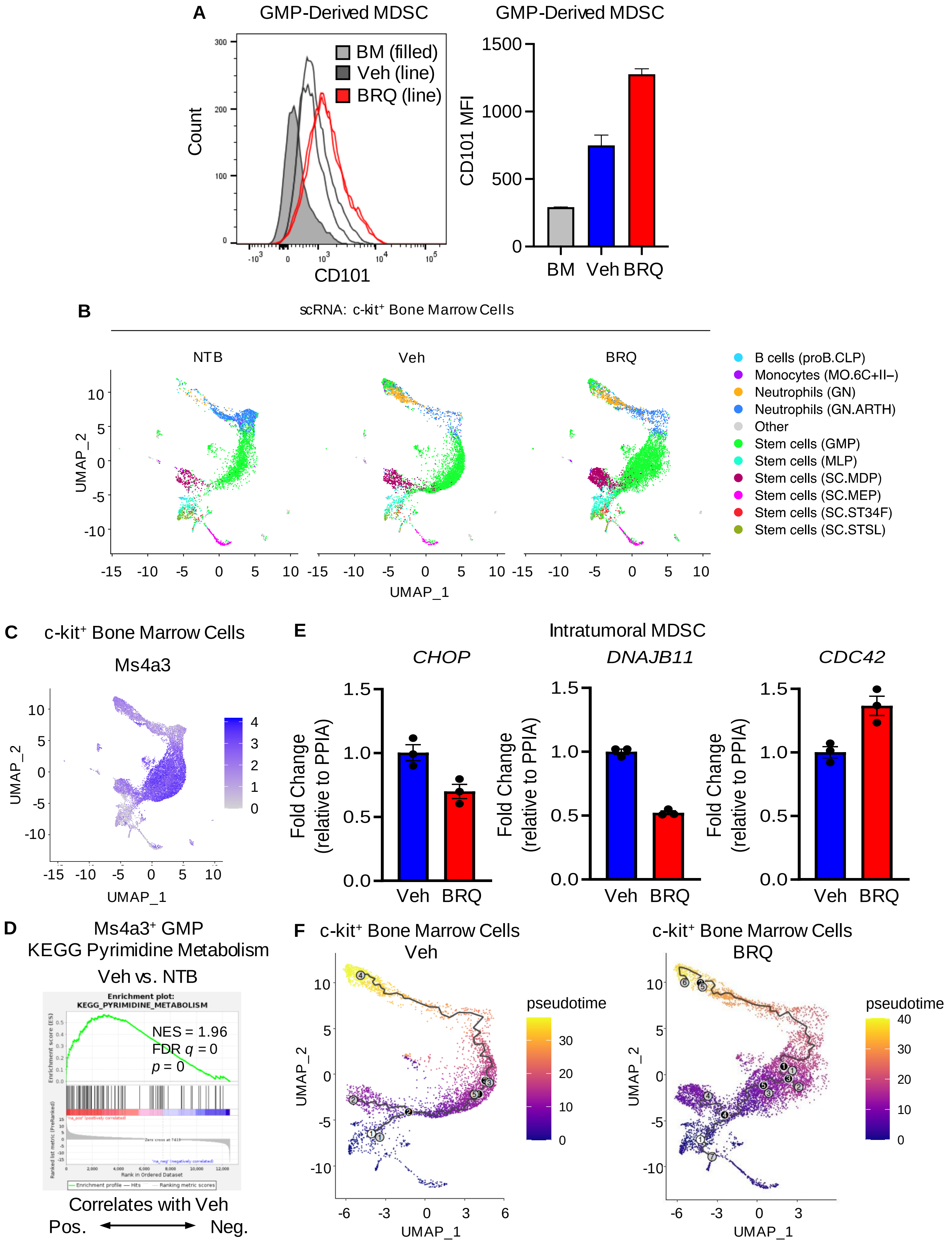

Supplemental Figure 8. Related to Figure 7. (***A***) Flow cytometry analysis of CD101 expression by GMP-derived MDSCs. CD101 expression is shown as histograms (*left*) and as mean fluorescent intensity (n=2 mice/group). (***B***) UMAP visualization of single-cell RNA sequencing of c-kit$^+$ BM cells isolated from non-tumor bearing (NTB) mice (*left*) and 4T1-bearing mice treated with vehicle (*middle*) or BRQ (*right*). For each experimental group, 3 biological replicates were pooled. Cells were annotated based on profiles obtained using the ImmGen datasets. (***C***) Expression pattern of *Ms4a3* in c-kit$^+$ BM cells. (***D***) GSEA of the KEGG pyrimidine metabolism pathway comparing Veh-GMPs *versus* NTB GMPs. (***E***) CD11b$^+$Gr-1$^+$ MDSCs were isolated from tumors of 4T1 tumor bearing mice (STEMCELL EasySep™ Mouse MDSC isolation kit) and analyzed by RT-qPCR for the indicated genes. Data in *E* are recorded as the mean ± SEM of triplicate determinations and is representative of 2-3 separate mice with similar results. All data is shown as mean ± SEM. (***F***) Differentiation trajectories of c-kit$^+$ bone marrow cells in 4T1-bearing mice treated with vehicle (*left*) or BRQ (*right*) as estimated using pseudotime analysis. Cells exhibiting high expression of the *Mecom* locus were defined as the origin point, marked as “1” in a white circle. Numbers in black backgrounds represent groups of clustering as defined by Monocole3 and in grey background represent cell populations.

Supplemental Figure 9

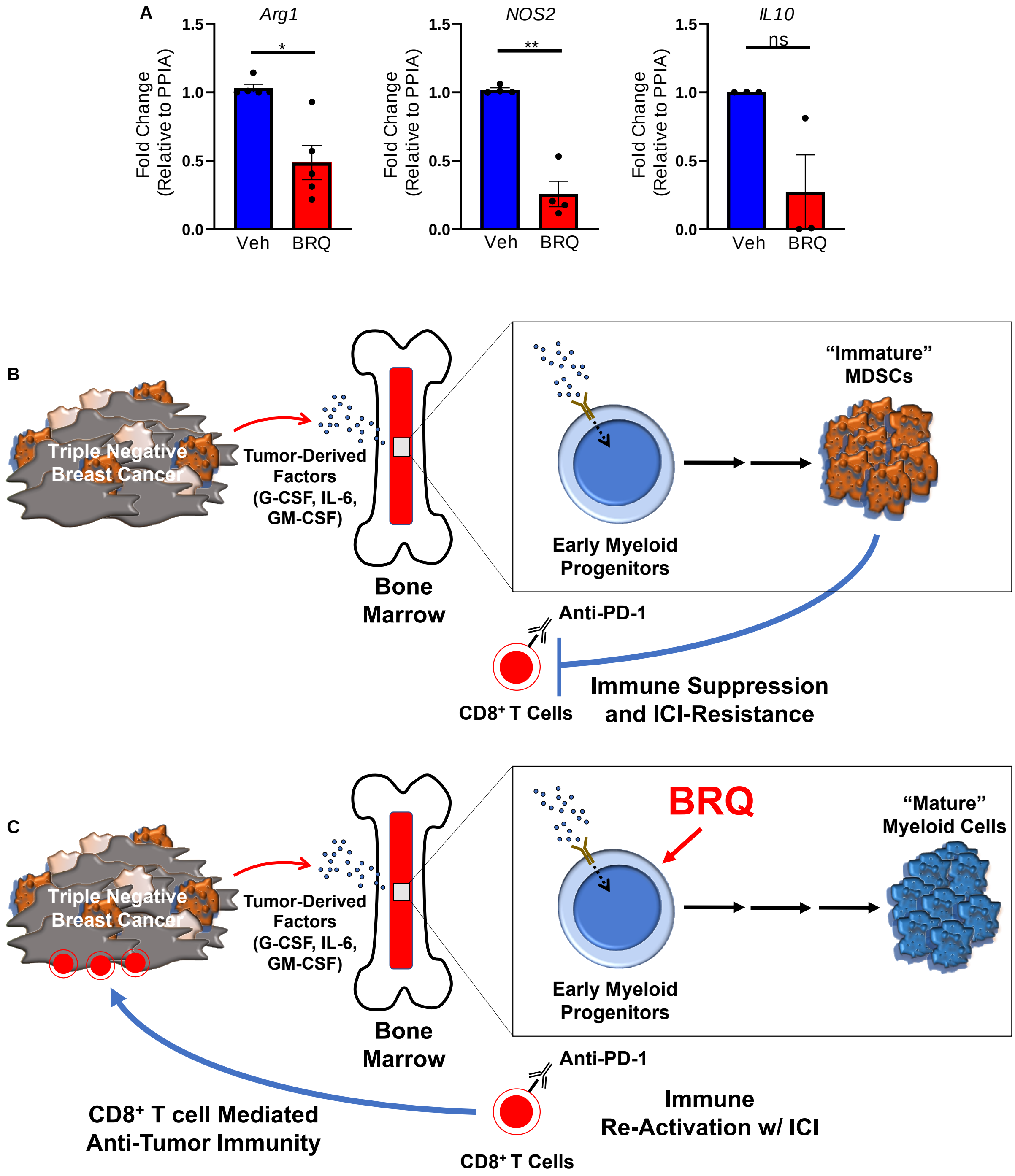

Supplemental Figure 9. Related to Figure 8. Overall model of the effects of BRQ on MDSC biogenesis and subsequent impact on ICI therapy. (***A***) Data in Figure 8D illustrated as the mean ± SEM of the fold-change in expression of *ARG1*, *iNOS*, or *IL10* of the indicated number of donors (denoted as individual data points) in which gene expression values were detectable. Paired *t*-test: ns = not significant; * = $p < 0.05$, ** = $p < 0.01$. (***B***) Tumor-derived factors (*e.g.*, G-CSF, IL-6, GM-CSF) enter the circulation and alter the differentiation of myeloid progenitors in the BM to immature MDSCs, which proceed to suppress antitumor immune responses through several mechanisms, including those mediated by $CD8^+$ T cells, and promote resistance to ICIs. (***C***) Treatment with BRQ rescues the ability of myeloid progenitors to differentiate into mature myeloid cells that have reduced immune suppressive function, thereby, removing a potential mechanism of resistance to ICIs, and enhancing their efficacy.

Supplemental Table 1: UP-REGULATED PATHWAYS: Veh-GMP vs NTB-GMP

Supplemental Table 1	SIZE	ES	NES	NOM p-val	FDR q-val	FWER p-val	RANK AT MAX	LEADING EDGE
REACTOME_MITOCHONDRIAL_PROTEIN_IMPORT	63	0.75633466	2.5076315	0	0	0	1492	tags=62%, list=12%, signal=70%
HALLMARK_MYC_TARGETS_V1	200	0.6617502	2.5050366	0	0	0	1510	tags=52%, list=12%, signal=58%
HALLMARK_MTORC1_SIGNALING	197	0.6441922	2.4409256	0	0	0	1710	tags=47%, list=14%, signal=53%
REACTOME_MITOCHONDRIAL_TRANSLATION	94	0.6977964	2.437391	0	0	0	2060	tags=64%, list=16%, signal=76%
REACTOME_UNFOLDED_PROTEIN_RESPONSE_UPR	84	0.6971273	2.4197595	0	0	0	1152	tags=40%, list=9%, signal=44%
HALLMARK_UNFOLDED_PROTEIN_RESPONSE	109	0.66847456	2.3734436	0	0	0	1806	tags=51%, list=14%, signal=59%
KEGG_PROTEIN_EXPORT	23	0.8609161	2.3597162	0	0	0	1152	tags=78%, list=9%, signal=86%
REACTOME_IRE1ALPHA_ACTIVATES_CHAPERONES	48	0.7330747	2.3357465	0	0	0	1152	tags=42%, list=9%, signal=46%
HALLMARK_MYC_TARGETS_V2	57	0.7140725	2.3294084	0	0	0	1866	tags=60%, list=15%, signal=70%
HALLMARK_OXIDATIVE_PHOSPHORYLATION	198	0.60727996	2.328873	0	0	0	2519	tags=55%, list=20%, signal=68%
WP_PHOTODYNAMIC_THERAPYINDUCED_UNFOLDED_PROTEIN_RESPONSE	25	0.82129556	2.3008738	0	0	0	997	tags=52%, list=8%, signal=56%
WP_PARKINUBIQUITIN_PROTEASOMAL_SYSTEM_PATHWAY	60	0.68089926	2.2362318	0	0	0	1839	tags=60%, list=15%, signal=70%
PID_MYC_ACTIV_PATHWAY	74	0.6619346	2.2184424	0	0	0	926	tags=36%, list=7%, signal=39%
REACTOME_CHOLESTEROL_BIOSYNTHESIS	24	0.7773575	2.1583455	0	4.71E-05	0.001	1630	tags=63%, list=13%, signal=72%
REACTOME_PROTEIN_LOCALIZATION	153	0.5819968	2.155983	0	4.40E-05	0.001	1104	tags=30%, list=9%, signal=33%
REACTOME_THE_ROLE_OF_GTSE1_IN_G2_M_PROGRESSION_AFTER_G2_CHECKPOINT	68	0.63932914	2.1553535	0	4.12E-05	0.001	1866	tags=54%, list=15%, signal=64%
REACTOME_SNRNP_ASSEMBLY	52	0.6668957	2.1353421	0	3.88E-05	0.001	1699	tags=52%, list=14%, signal=60%
REACTOME_RRNA_MODIFICATION_IN_THE_NUCLEUS_AND_CYTOSOL	59	0.6480189	2.1066806	0	1.11E-04	0.003	1979	tags=68%, list=16%, signal=80%
REACTOME_FORMATION_OF_TUBULIN_FOLDING_INTERMEDIATES_BY_CCT_TRIC	18	0.80651355	2.1039119	0	1.05E-04	0.003	1124	tags=61%, list=9%, signal=67%
REACTOME_TRNA_PROCESSING	103	0.5969597	2.0988505	0	1.32E-04	0.004	3348	tags=60%, list=27%, signal=81%
REACTOME_HSP90_CHAPERONE_CYCLE_FOR_STEROID_HORMONE_RECEPTORS_SHR	44	0.6648442	2.09539	0	1.25E-04	0.004	1223	tags=39%, list=10%, signal=43%
REACTOME_CYTOSOLIC_TRNA_AMINOACYLATION	24	0.7437306	2.091962	0	1.20E-04	0.004	1744	tags=79%, list=14%, signal=92%
KEGG_SPLICEOSOME	122	0.5759891	2.0844872	0	1.14E-04	0.004	2927	tags=58%, list=23%, signal=75%
REACTOME_SCF_SKP2_MEDIATED_DEGRADATION_OF_P27_P21	58	0.6362173	2.0791385	0	1.10E-04	0.004	1839	tags=57%, list=15%, signal=66%
REACTOME_PROCESSING_OF_CAPPED_INTRON_CONTAINING_PRE_MRNA	236	0.54047656	2.0765417	0	1.05E-04	0.004	2927	tags=51%, list=23%, signal=65%
REACTOME_BINDING_AND_UPTAKE_OF_LIGANDS_BY_SCAVENGER_RECEPTORS	27	0.7429928	2.0744722	0	1.26E-04	0.005	790	tags=33%, list=6%, signal=35%
REACTOME_TRNA_PROCESSING_IN_THE_NUCLEUS	56	0.64276433	2.0638926	0	1.71E-04	0.007	3311	tags=70%, list=26%, signal=94%
REACTOME_INTERACTIONS_OF_REV_WITH_HOST_CELLULAR_PROTEINS	36	0.68275034	2.0553727	0	1.65E-04	0.007	2862	tags=69%, list=23%, signal=90%
REACTOME_PERK_REGULATES_GENE_EXPRESSION	27	0.72086835	2.0458345	0	2.51E-04	0.01	2133	tags=67%, list=17%, signal=80%
REACTOME_N_GLYCAN_TRIMMING_IN_THE_ER_AND_CALNEXIN_CALRETICULIN_CYCLE	35	0.6763585	2.0431304	0	2.64E-04	0.011	1407	tags=37%, list=11%, signal=42%
REACTOME_MRNA_SPLICING_MINOR_PATHWAY	52	0.6259526	2.0370023	0	2.77E-04	0.012	1935	tags=52%, list=15%, signal=61%
REACTOME_MRNA_SPLICING	185	0.5425178	2.0368354	0	2.69E-04	0.012	2772	tags=49%, list=22%, signal=62%
REACTOME_HEDGEHOG_LIGAND_BIOGENESIS	58	0.6352474	2.035358	0	2.61E-04	0.012	1839	tags=55%, list=15%, signal=64%
REACTOME_METABOLISM_OF_POLYAMINES	57	0.61690074	2.0341797	0	2.53E-04	0.012	1839	tags=56%, list=15%, signal=65%
REACTOME_DECTIN_1_MEDIATED_NONCANONICAL_NF_KB_SIGNALING	60	0.6151846	2.0289862	0	2.46E-04	0.012	1839	tags=52%, list=15%, signal=60%
REACTOME_DEGRADATION_OF_DVL	55	0.6166691	2.0164287	0	3.49E-04	0.018	1839	tags=53%, list=15%, signal=61%
REACTOME_ATTENUATION_PHASE	20	0.74097884	2.0100257	0	4.36E-04	0.024	1223	tags=50%, list=10%, signal=55%
REACTOME_DEGRADATION_OF_GLI1_BY_THE_PROTEASOME	57	0.61503464	2.0035737	0	4.24E-04	0.024	1839	tags=54%, list=15%, signal=63%
REACTOME_AUF1_HNRNP_D0_BINDS_AND_DESTABILIZES_MRNA	53	0.6270777	1.9944234	0	5.46E-04	0.031	1839	tags=57%, list=15%, signal=66%
WP_CHOLESTEROL_BIOSYNTHESIS_PATHWAY	15	0.8028484	1.9941008	0	5.48E-04	0.032	1507	tags=73%, list=12%, signal=83%
REACTOME_NEGATIVE_REGULATION_OF_NOTCH4_SIGNALING	54	0.61807865	1.9876544	0	6.15E-04	0.037	1839	tags=56%, list=15%, signal=65%
REACTOME_CELLULAR_RESPONSE_TO_HYPOXIA	72	0.5822219	1.9856781	0	6.00E-04	0.037	1891	tags=49%, list=15%, signal=57%
WP_TRANSLATION_FACTORS	49	0.6276136	1.9855944	0	5.87E-04	0.037	911	tags=45%, list=7%, signal=48%
REACTOME_ABC_TRANSPORTER_DISORDERS	68	0.5896624	1.9814249	0	6.18E-04	0.04	1839	tags=49%, list=15%, signal=57%
REACTOME_DEFECTIVE_CFTR_CAUSES_CYSTIC_FIBROSIS	60	0.6048607	1.9740257	0	8.19E-04	0.054	1839	tags=53%, list=15%, signal=62%
REACTOME_HOST_INTERACTIONS_OF_HIV_FACTORS	127	0.5426169	1.9704609	0	8.72E-04	0.059	1839	tags=44%, list=15%, signal=51%
KEGG_PYRIMIDINE_METABOLISM	87	0.5678796	1.9665905	0	9.77E-04	0.068	2896	tags=51%, list=23%, signal=65%
REACTOME_REGULATION_OF_MRNA_STABILITY_BY_PROTEINS_THAT_BIND_AU_RICH_ELEMENTS	84	0.57296264	1.9640201	0	9.84E-04	0.07	1839	tags=49%, list=15%, signal=57%
REACTOME_REGULATION_OF_RUNX3_EXPRESSION_AND_ACTIVITY	54	0.61396873	1.9622132	0	9.77E-04	0.071	1981	tags=56%, list=16%, signal=66%
REACTOME_DEGRADATION_OF_AXIN	54	0.60313004	1.9610182	0	0.001010472	0.075	1839	tags=52%, list=15%, signal=60%
KEGG_PROTEASOME	42	0.63383806	1.9605844	0	9.91E-04	0.075	1839	tags=60%, list=15%, signal=69%
REACTOME_POSTMITOTIC_NUCLEAR_PORE_COMPLEX_NPC_REFORMATION	27	0.68738467	1.9586958	0	0.001059482	0.081	2862	tags=70%, list=23%, signal=91%
REACTOME_TRANSPORT_OF_THE_SLBP_DEPENDANT_MATURE_MRNA	35	0.6575658	1.9573525	0	0.001057012	0.084	3311	tags=71%, list=26%, signal=97%
REACTOME_CROSS_PRESENTATION_OF_SOLUBLE_EXOGENOUS_ANTIGENS_ENDOSOMES	46	0.6283065	1.9558874	0	0.001038137	0.084	1839	tags=59%, list=15%, signal=69%
REACTOME_COOPERATION_OF_PREFOLDIN_AND_TRIC_CCT_IN_ACTIN_AND_TUBULIN_FOLDING	25	0.67711616	1.9490722	0	0.001124532	0.093	1706	tags=56%, list=14%, signal=65%
REACTOME_HSF1_DEPENDENT_TRANSACTIVATION	29	0.6827732	1.9459245	0	0.001105144	0.093	1223	tags=34%, list=10%, signal=38%
REACTOME_THE_CITRIC_ACID_TCA_CYCLE_AND_RESPIRATORY_ELECTRON_TRANSPORT	168	0.5171471	1.9388535	0	0.001254493	0.107	1997	tags=41%, list=16%, signal=48%
REACTOME_REGULATION_OF_HMOX1_EXPRESSION_AND_ACTIVITY	64	0.58295184	1.9349111	0	0.001354531	0.117	2311	tags=55%, list=18%, signal=67%
REACTOME_ASSEMBLY_OF_THE_PRE_REPLICATIVE_COMPLEX	67	0.5789358	1.934509	0	0.001342957	0.118	2311	tags=54%, list=18%, signal=65%
REACTOME_ASYMMETRIC_LOCALIZATION_OF_PCP_PROTEINS	60	0.5881409	1.9318899	0	0.001342698	0.12	1839	tags=48%, list=15%, signal=56%
REACTOME_UCH_PROTEINASES	88	0.5488004	1.9316857	0	0.001321385	0.12	2051	tags=49%, list=16%, signal=58%
REACTOME_INTERACTIONS_OF_VPR_WITH_HOST_CELLULAR_PROTEINS	35	0.64170414	1.9269229	0	0.001403656	0.129	3311	tags=71%, list=26%, signal=97%
REACTOME_PIWI_INTERACTING_RNA_PIRNA_BIOGENESIS	20	0.72449607	1.9258968	0	0.001382061	0.129	1935	tags=55%, list=15%, signal=65%
REACTOME_ORC1_REMOVAL_FROM_CHROMATIN	69	0.5707456	1.9254524	0	0.001381375	0.131	2311	tags=52%, list=18%, signal=64%
REACTOME_G1_S_DNA_DAMAGE_CHECKPOINTS	65	0.57766867	1.9232252	0	0.001399958	0.134	1839	tags=46%, list=15%, signal=54%
REACTOME_EXPORT_OF_VIRAL_RIBONUCLEOPROTEINS_FROM_NUCLEUS	32	0.65806085	1.9213716	0	0.001418357	0.136	3311	tags=72%, list=26%, signal=97%
REACTOME_STABILIZATION_OF_P53	55	0.5967427	1.9202098	0	0.001426555	0.139	2331	tags=56%, list=19%, signal=69%
REACTOME_CELLULAR_RESPONSE_TO_HEAT_STRESS	91	0.55202895	1.9179188	0	0.001491508	0.146	1669	tags=40%, list=13%, signal=45%
REACTOME_SELECTIVE_AUTOPHAGY	68	0.5683598	1.9176773	0	0.001470501	0.146	2386	tags=44%, list=19%, signal=54%
REACTOME_REGULATION_OF_HSF1_MEDIATED_HEAT_SHOCK_RESPONSE	73	0.5685432	1.9134498	0	0.001597199	0.161	2292	tags=51%, list=18%, signal=62%
REACTOME_VIRAL_MESSENGER_RNA_SYNTHESIS	43	0.6189895	1.9123586	0	0.001611459	0.165	2862	tags=65%, list=23%, signal=84%
REACTOME_REGULATION_OF_PTEN_STABILITY_AND_ACTIVITY	66	0.574757	1.9085933	0	0.001669551	0.173	2311	tags=53%, list=18%, signal=65%
WP_ELECTRON_TRANSPORT_CHAIN_OXPHOS_SYSTEM_IN_MITOCHONDRIA	98	0.54562885	1.9080461	0	0.001673905	0.175	1997	tags=49%, list=16%, signal=58%
REACTOME_RESPIRATORY_ELECTRON_TRANSPORT_ATP_SYNTHESIS_BY_CHEMIOSMOTIC_COUPLING_AND_HEAT_PRODUCTION_BY_UNCOUPLING_PROTEINS	122	0.5265745	1.9056871	0	0.001739236	0.184	1997	tags=44%, list=16%, signal=52%
REACTOME_REGULATION_OF_RAS_BY_GAPS	66	0.5775617	1.9034631	0	0.001793866	0.193	1839	tags=45%, list=15%, signal=53%
REACTOME_SUMOYLATION_OF_UBIQUITINYLATION_PROTEINS	38	0.6285457	1.9019636	0	0.001813394	0.198	3311	tags=63%, list=26%, signal=85%
REACTOME_TRANSLATION	285	0.4879495	1.9002172	0	0.001832155	0.203	1615	tags=42%, list=13%, signal=47%
KEGG_HUNTINGTONS_DISEASE	157	0.5116294	1.8985952	0	0.001858941	0.209	2510	tags=45%, list=20%, signal=56%
WP_MITOCHONDRIAL_CIV_ASSEMBLY	33	0.6551229	1.8963859	0	0.001942206	0.219	2050	tags=64%, list=16%, signal=76%
REACTOME_REGULATION_OF_RUNX2_EXPRESSION_AND_ACTIVITY	66	0.57017493	1.895329	0	0.001942941	0.222	1839	tags=48%, list=15%, signal=56%
REACTOME_NS1_MEDIATED_EFFECTS_ON_HOST_PATHWAYS	38	0.6235859	1.8937917	0	0.001959391	0.226	3311	tags=68%, list=26%, signal=93%
REACTOME_SWITCHING_OF_ORIGINS_TO_A_POST_REPLICATIVE_STATE	89	0.54326445	1.8907788	0	0.002116829	0.246	3096	tags=55%, list=25%, signal=73%
REACTOME_SUMOYLATION_OF_SUMOYLATION_PROTEINS	34	0.64401835	1.8879756	0	0.00223924	0.26	3311	tags=71%, list=26%, signal=96%
WP_CHOLESTEROL_METABOLISM_INCLUDES_BOTH_BLOCH_AND_KANDUTSCHRUSSELL_PATHWAYS	42	0.6061334	1.8862392	0	0.002282581	0.267	1915	tags=45%, list=15%, signal=53%
KEGG_OXIDATIVE_PHOSPHORYLATION	115	0.52042466	1.8848464	0	0.002324811	0.274	1249	tags=38%, list=10%, signal=42%
REACTOME_RESPIRATORY_ELECTRON_TRANSPORT	100	0.5327597	1.8839842	0	0.002313184	0.276	1898	tags=44%, list=15%, signal=51%
REACTOME_ABC_FAMILY_PROTEINS_MEDIATED_TRANSPORT	90	0.5412548	1.8789381	0	0.002443705	0.289	2333	tags=49%, list=19%, signal=60%
REACTOME_DNA_REPLICATION_PRE_INITIATION	83	0.5418617	1.8785741	0	0.002446013	0.291	2417	tags=49%, list=19%, signal=61%
REACTOME_APC_C_MEDIATED_DEGRADATION_OF_CELL_CYCLE_PROTEINS	86	0.53881735	1.8706335	0	0.002716964	0.321	2051	tags=44%, list=16%, signal=52%
WP_PYRIMIDINE_METABOLISM	77	0.542451	1.8586818	0	0.00317074	0.368	2896	tags=49%, list=23%, signal=64%
WP_TRANSSULFURATION_AND_ONE_CARBON_METABOLISM	26	0.65929216	1.8572206	0	0.003180398	0.377	2062	tags=42%, list=16%, signal=51%
REACTOME_RHOBTB2_GTPASE_CYCLE	23	0.6752529	1.8544476	0	0.00325623	0.384	502	tags=30%, list=4%, signal=32%
REACTOME_CELLULAR_RESPONSE_TO_CHEMICAL_STRESS	143	0.50905246	1.852208	0	0.003350979	0.395	2074	tags=43%, list=17%, signal=51%
REACTOME_REGULATION_OF_GLUCOKINASE_BY_GLUCOKINASE_REGULATORY_PROTEIN	29	0.6494919	1.8500642	0	0.003417499	0.407	3311	tags=72%, list=26%, signal=98%
REACTOME_DISORDERS_OF_TRANSMEMBRANE_TRANSPORTERS	139	0.50529623	1.840954	0	0.00390683	0.446	1700	tags=36%, list=14%, signal=41%
REACTOME_APC_C_CDH1_MEDIATED_DEGRADATION_OF_CDC20_AND_OTHER_APC_C_CDH1_TARGETED_PROTEINS_IN_LATE_MITOSIS_EARLY_G1	73	0.55132675	1.8409238	0	0.003868148	0.446	2051	tags=47%, list=16%, signal=55%
KEGG_PARKINSONS_DISEASE	114	0.5139305	1.8387903	0	0.003915331	0.456	949	tags=34%, list=8%, signal=37%
REACTOME_CYTOPROTECTION_BY_HMOX1	116	0.5130181	1.8371662	0	0.004004723	0.464	2074	tags=43%, list=17%, signal=51%
REACTOME_SIGNALING_BY_NOTCH4	77	0.5321425	1.8304968	0	0.004397551	0.5	1902	tags=42%, list=15%, signal=49%
REACTOME_ASPARAGINE_N_LINKED_GLYCOSYLATION	261	0.47544378	1.8295697	0	0.004424793	0.504	2708	tags=38%, list=22%, signal=47%
REACTOME_SUMOYLATION_OF_RNA_BINDING_PROTEINS	45	0.5913317	1.8258063	0	0.004562262	0.518	3917	tags=71%, list=31%, signal=103%
REACTOME_COPI_MEDIATED_ANTEROGRADE_TRANSPORT	87	0.52186215	1.8227646	0	0.004701611	0.533	2846	tags=43%, list=23%, signal=55%
REACTOME_TRNA_AMINOACYLATION	42	0.5789524	1.8186094	0	0.004920617	0.558	2338	tags=60%, list=19%, signal=73%
REACTOME_DNA_REPLICATION	125	0.5038711	1.8177216	0	0.004929584	0.561	3143	tags=50%, list=25%, signal=67%
REACTOME_NUCLEAR_PORE_COMPLEX_NPC_DISASSEMBLY	35	0.6064137	1.8112493	0	0.005406906	0.599	3311	tags=66%, list=26%, signal=89%
REACTOME_HEDGEHOG_ON_STATE	78	0.5291605	1.8065946	0	0.005674418	0.622	2051	tags=41%, list=16%, signal=49%
WP_STEROL_REGULATORY_ELEMENTBINDING_PROTEINS_SREBP_SIGNALLING	66	0.5411703	1.8013854	0	0.006045511	0.653	1507	tags=36%, list=12%, signal=41%
REACTOME_REGULATION_OF_CHOLESTEROL_BIOSYNTHESIS_BY_SREBP_SREBF	54	0.56103754	1.7997845	0	0.006117068	0.66	1507	tags=35%, list=12%, signal=40%
REACTOME_NUCLEAR_ENVELOPE_NE_REASSEMBLY	63	0.54963917	1.7986697	0	0.006187882	0.666	2271	tags=44%, list=18%, signal=54%
REACTOME_MITOPHAGY	29	0.6263923	1.7854097	0	0.007223351	0.737	2386	tags=55%, list=19%, signal=68%

Supplemental Table 1: UP-REGULATED PATHWAYS: Veh-GMP vs NTB-GMP

REACTOME HIV INFECTION	220	0.47264457	1.784778	0	0.007154678	0.74	2311	tags=39%, list=18%, signal=47%
REACTOME_TNFR2_NON_CANONICAL_NF_KB_PATHWAY	88	0.5068262	1.7814027	0	0.007439601	0.755	2051	tags=38%, list=16%, signal=45%
REACTOME_DEGRADATION_OF_BETA_CATENIN_BY_THE_DESTRUCTION_COMPLEX	83	0.5152622	1.7806194	0	0.007443595	0.758	1839	tags=42%, list=15%, signal=49%
REACTOME INTERLEUKIN 1 FAMILY SIGNALING	123	0.49130273	1.7802838	0	0.007416355	0.761	2311	tags=35%, list=18%, signal=42%
REACTOME_HEDGEHOG_OFF_STATE	99	0.49939418	1.7626015	0	0.009551792	0.848	1700	tags=35%, list=14%, signal=41%
REACTOME_FCERI_MEDIATED_NF_KB_ACTIVATION	79	0.5142169	1.7583783	0	0.010054316	0.861	1839	tags=39%, list=15%, signal=46%
HALLMARK_PROTEIN_SECRETION	91	0.4953044	1.73945	0	0.012334627	0.923	3006	tags=47%, list=24%, signal=62%
REACTOME CYCLIN A CDK2 ASSOCIATED EVENTS AT S PHASE ENTRY	83	0.5050401	1.7388283	0	0.012359559	0.926	2552	tags=49%, list=20%, signal=62%
REACTOME_HIV_LIFE_CYCLE	141	0.4660513	1.7358283	0	0.012810368	0.931	4370	tags=60%, list=35%, signal=90%
REACTOME_PROTEIN_FOLDING	82	0.4940577	1.7276487	0	0.01419995	0.952	3269	tags=46%, list=26%, signal=62%
REACTOME_CLEC7A_DECTIN_1_SIGNALING	95	0.49884167	1.7226365	0	0.014918426	0.963	1700	tags=35%, list=14%, signal=40%
REACTOME TRANSCRIPTIONAL REGULATION BY RUNX2	96	0.49675816	1.7182623	0	0.01557062	0.972	1700	tags=36%, list=14%, signal=42%
HALLMARK_UV_RESPONSE_UP	133	0.4695518	1.7159204	0	0.01578286	0.974	2280	tags=34%, list=18%, signal=41%
REACTOME_PCP_CE_PATHWAY	83	0.4993352	1.7158546	0	0.015697997	0.974	2068	tags=43%, list=16%, signal=52%
REACTOME ANTIGEN PROCESSING CROSS PRESENTATION	96	0.48595154	1.7142498	0	0.01563955	0.978	1700	tags=40%, list=14%, signal=45%
REACTOME_MITOTIC_METAPHASE_AND_ANAPHASE	218	0.44884393	1.7087148	0	0.016468864	0.983	3421	tags=46%, list=27%, signal=63%
REACTOME_ER_TO_GOLGI_ANTEROGRADE_TRANSPORT	133	0.46700668	1.7016302	0	0.017507322	0.989	2900	tags=41%, list=23%, signal=53%
WP_MRNA_PROCESSING	125	0.46727526	1.6899337	0	0.019957125	0.996	2448	tags=38%, list=19%, signal=47%
REACTOME TRANSCRIPTIONAL REGULATION BY RUNX3	86	0.48991132	1.6895659	0	0.019919429	0.996	1981	tags=36%, list=16%, signal=43%
REACTOME_MITOTIC_G1_PHASE_AND_G1_S_TRANSITION	143	0.4597274	1.6833187	0	0.02139442	0.997	2436	tags=41%, list=19%, signal=51%
REACTOME_SEPARATION_OF_SISTER_CHROMATIDS	176	0.44122225	1.6623284	0	0.026283136	0.998	2966	tags=41%, list=24%, signal=54%
REACTOME FORMATION OF THE EARLY ELONGATION COMPLEX	32	0.5622357	1.6550459	0	0.027877312	0.999	2063	tags=50%, list=16%, signal=60%
HALLMARK_DNA_REPAIR	145	0.45375726	1.6453923	0	0.030854814	0.999	3231	tags=41%, list=26%, signal=55%
REACTOME_NEDDYLATION	216	0.42685068	1.6396842	0	0.03201649	1	2311	tags=31%, list=18%, signal=37%
HALLMARK_ANDROGEN_RESPONSE	89	0.4704051	1.6341927	0	0.03364496	1	850	tags=24%, list=7%, signal=25%
REACTOME MITOTIC G2 G2 M PHASES	184	0.43567508	1.6309934	0	0.03402652	1	2552	tags=36%, list=20%, signal=45%
REACTOME_METABOLISM_OF_STEROIDS	109	0.4599352	1.6288463	0	0.034613088	1	1684	tags=28%, list=13%, signal=32%
KEGG_ALZHEIMERS_DISEASE	142	0.44413564	1.625007	0	0.035875358	1	949	tags=25%, list=8%, signal=26%
REACTOME CLASS I MHC MEDIATED ANTIGEN PROCESSING PRESENTATION	337	0.39297774	1.5442529	0	0.066766046	1	1878	tags=25%, list=15%, signal=29%
REACTOME M PHASE	352	0.3795135	1.4712917	0	0.111652635	1	3041	tags=37%, list=24%, signal=48%
REACTOME_CELL_CYCLE_MITOTIC	488	0.35594422	1.4280461	0	0.14370182	1	3041	tags=36%, list=24%, signal=45%
REACTOME_CELL_CYCLE_CHECKPOINTS	258	0.38074422	1.4672042	0.001042753	0.114604786	1	3172	tags=39%, list=25%, signal=51%
REACTOME UB SPECIFIC PROCESSING PROTEASES	167	0.4253997	1.5855602	0.001090513	0.050118733	1	3404	tags=44%, list=27%, signal=59%
REACTOME_TRANSPORT_TO_THE_GOLGI_AND_SUBSEQUENT_MODIFICATION	155	0.42078575	1.562619	0.001094092	0.05777438	1	2900	tags=38%, list=23%, signal=49%
WP_NONALCOHOLIC_FATTY_LIVER_DISEASE	136	0.4722839	1.7138021	0.00110011	0.015645495	0.979	2070	tags=35%, list=16%, signal=41%
REACTOME_G2_M_CHECKPOINTS	140	0.4159355	1.54767	0.001102536	0.06485791	1	2351	tags=36%, list=19%, signal=44%
KEGG PURINE METABOLISM	129	0.45132685	1.6401159	0.001128668	0.032033388	1	2838	tags=40%, list=23%, signal=51%
REACTOME_C_TYPE_LECTIN_RECEPTORS_CLRS	120	0.4537874	1.6379962	0.001129944	0.032450285	1	1700	tags=31%, list=14%, signal=35%
REACTOME_AUTOPHAGY	130	0.4458778	1.6139069	0.001133787	0.039760552	1	2386	tags=32%, list=19%, signal=39%
REACTOME_SIGNALING_BY_HEDGEHOG	128	0.4730427	1.7166644	0.001136364	0.015713686	0.974	1700	tags=31%, list=14%, signal=36%
REACTOME INTERLEUKIN 1 SIGNALING	95	0.5395375	1.866642	0.001156069	0.002844918	0.333	2051	tags=39%, list=16%, signal=46%
REACTOME_TRANSPORT_OF_MATURE_TRANSCRIPT_TO_CYTOPLASM	81	0.49284795	1.6754023	0.001162791	0.023349684	0.997	3414	tags=56%, list=27%, signal=76%
REACTOME_DOWNSTREAM_SIGNALING_EVENTS_OF_B_CELL_RECEPTOR_BCR	79	0.51711845	1.7729496	0.001177856	0.0083178	0.802	1839	tags=42%, list=15%, signal=49%
REACTOME ANTIVIRAL MECHANISM BY IFN STIMULATED GENES	74	0.49231914	1.6753157	0.00120048	0.02322915	0.997	2966	tags=50%, list=24%, signal=65%
WP_AMINO_ACID_METABOLISM	74	0.470371	1.5926766	0.001201923	0.04732306	1	2077	tags=36%, list=17%, signal=43%
WP_PROTEASOME_DEGRADATION	57	0.56100214	1.8404801	0.001218027	0.003882173	0.45	1839	tags=53%, list=15%, signal=61%
WP_OXIDATIVE_PHOSPHORYLATION	58	0.50748575	1.6788697	0.001218027	0.022549039	0.997	2510	tags=52%, list=20%, signal=64%
REACTOME_TRANSPORT_OF_MATURE_MRNAS_DERIVED_FROM_INTRONLESS_TRANSCRIPTS	42	0.574639	1.7581222	0.00125	0.010033252	0.861	2862	tags=57%, list=23%, signal=74%
REACTOME PEPTIDE HORMONE METABOLISM	49	0.52209765	1.6425855	0.001278772	0.03168176	1	1632	tags=24%, list=13%, signal=28%
REACTOME CRISTAE FORMATION	31	0.5935612	1.7451835	0.001303781	0.011580212	0.904	2467	tags=58%, list=20%, signal=72%
REACTOME_SUMOYLATION_OF_DNA_REPLICATION_PROTEINS	45	0.6250848	1.957468	0.001305483	0.001076586	0.084	3321	tags=67%, list=26%, signal=90%
REACTOME_NUCLEAR_ENVELOPE_BREAKDOWN	51	0.53188276	1.6952258	0.001310616	0.018882813	0.994	3311	tags=61%, list=26%, signal=82%
REACTOME NUCLEAR IMPORT OF REV PROTEIN	33	0.67737603	2.0123038	0.001319261	3.75E-04	0.02	2862	tags=70%, list=23%, signal=90%
REACTOME_ASSOCIATION_OF_TRIC_CCT_WITH_TARGET_PROTEINS_DURING_BIOSYNTHESIS	39	0.5769091	1.7499135	0.00135318	0.011040126	0.888	3248	tags=46%, list=26%, signal=62%
REACTOME_MICRORNA_MIRNA_BIOGENESIS	24	0.6547149	1.8015319	0.001369863	0.006091916	0.653	1935	tags=50%, list=15%, signal=59%
REACTOME_ATF4_ACTIVATES_GENES_IN_RESPONSE_TO_ENDOPLASMIC_RETICULUM_STRESS	22	0.6735496	1.8291607	0.001390821	0.004396058	0.505	2630	tags=68%, list=21%, signal=86%
REACTOME_PINK1_PRKN_MEDIATED_MITOPHAGY	22	0.6927023	1.8576069	0.001396648	0.003213876	0.377	2386	tags=59%, list=19%, signal=73%
BIOCARTA_PROTEASOME_PATHWAY	19	0.71707374	1.8633913	0.001422475	0.002956649	0.345	2552	tags=74%, list=20%, signal=92%
WP_NSP1_FROM_SARSCOV2_INHIBITS_TRANSLATION_INITIATION_IN_THE_HOST_CELL	16	0.7213532	1.7964023	0.001424501	0.006356798	0.686	752	tags=56%, list=6%, signal=60%
REACTOME ANTIGEN PROCESSING UBIQUITINATION PROTEASOME DEGRADATION	278	0.38237548	1.4761068	0.002068252	0.10768061	1	1878	tags=24%, list=15%, signal=28%
HALLMARK_E2F_TARGETS	199	0.40039772	1.514249	0.002125399	0.08154002	1	2904	tags=37%, list=23%, signal=47%
REACTOME_S_PHASE	159	0.4261178	1.5989468	0.002157497	0.045222435	1	3143	tags=43%, list=25%, signal=57%
REACTOME_PROGRAMMED_CELL_DEATH	187	0.3814418	1.4390454	0.002181025	0.13516736	1	2238	tags=31%, list=18%, signal=37%
REACTOME NUCLEOTIDE EXCISION REPAIR	108	0.42163974	1.516466	0.002277904	0.08062409	1	2128	tags=34%, list=17%, signal=41%
REACTOME_TCR_SIGNALING	103	0.44193196	1.565138	0.002293578	0.05704033	1	1916	tags=33%, list=15%, signal=39%
REACTOME_METABOLISM_OF_NUCLEOTIDES	81	0.4858876	1.6614755	0.002339181	0.026381228	0.998	2753	tags=40%, list=22%, signal=50%
REACTOME_BIOSYNTHESIS_OF_THE_N_GLYCAN_PRECURSOR_DOLICHOL_LIPID_LINKED_OLIGOSACCHARIDE_LLO_AND_T RANSFER_TO_A_NASCENT_PROTEIN	66	0.47275996	1.5689884	0.002453988	0.055581283	1	3516	tags=47%, list=28%, signal=65%
REACTOME_SUMOYLATION_OF_CHROMATIN_ORGANIZATION_PROTEINS	55	0.51925	1.7059186	0.002518892	0.016773175	0.987	3808	tags=62%, list=30%, signal=88%
KEGG_RNA_DEGRADATION	54	0.5163005	1.6589723	0.00255102	0.026820438	0.999	2273	tags=43%, list=18%, signal=52%
WP METABOLIC REPROGRAMMING IN COLON CANCER	41	0.5796807	1.7850642	0.002567394	0.00718079	0.738	1545	tags=44%, list=12%, signal=50%
REACTOME_CALNEXIN_CALRETICULIN_CYCLE	26	0.65134203	1.8335439	0.002642008	0.004231401	0.485	1407	tags=31%, list=11%, signal=35%
REACTOME_AGGREPHAGY	31	0.58656687	1.7032477	0.002663116	0.017310303	0.989	1124	tags=32%, list=9%, signal=35%
KEGG RNA POLYMERASE	27	0.5953314	1.6711054	0.002743484	0.024199165	0.998	2838	tags=63%, list=23%, signal=81%
REACTOME_FGFR2_ALTERNATIVE_SPLICING	24	0.63474977	1.7829103	0.002754821	0.007345112	0.749	2172	tags=58%, list=17%, signal=70%
REACTOME_FGFR2_MUTANT_RECEPTOR_ACTIVATION	20	0.6462881	1.7421129	0.002808989	0.011964343	0.913	1935	tags=55%, list=15%, signal=65%
WP_MITOCHONDRIAL_CIII_ASSEMBLY	16	0.6670167	1.7077502	0.002915452	0.016546544	0.984	989	tags=38%, list=8%, signal=41%
HALLMARK ADIPOGENESIS	187	0.38005754	1.4356384	0.003236246	0.13755219	1	2486	tags=35%, list=20%, signal=43%
REACTOME_APOPTOSIS	162	0.40715602	1.5026617	0.003253796	0.088929124	1	2238	tags=33%, list=18%, signal=40%
REACTOME_METABOLISM_OF_WATER_SOLUBLE_VITAMINS_AND_COFACTORS	101	0.42615953	1.4951812	0.003409091	0.09455568	1	3971	tags=42%, list=32%, signal=60%
REACTOME_GLUCOSE_METABOLISM	79	0.46783787	1.6138917	0.003525264	0.039562352	1	1635	tags=37%, list=13%, signal=42%
REACTOME MAPK6 MAPK4 SIGNALING	83	0.49103725	1.696092	0.003541913	0.018792914	0.994	1839	tags=39%, list=15%, signal=45%
REACTOME_TP53_REGULATES_METABOLIC_GENES	83	0.45527777	1.5690778	0.003645201	0.055789925	1	1446	tags=28%, list=12%, signal=31%
REACTOME_GLYCOLYSIS	63	0.46875396	1.5549659	0.003745318	0.06177459	1	1635	tags=37%, list=13%, signal=42%
REACTOME HSF1 ACTIVATION	23	0.6241021	1.71434	0.004092769	0.015722366	0.978	1223	tags=43%, list=10%, signal=48%
REACTOME_RNA_POLYMERASE_III_TRANSCRIPTION_INITIATION_FROM_TYPE_1_PROMOTER	28	0.577157	1.6591259	0.004115226	0.026922446	0.998	4633	tags=79%, list=37%, signal=124%
WP_UNFOLDED_PROTEIN_RESPONSE	23	0.637676	1.7486972	0.004132231	0.011163838	0.895	663	tags=22%, list=5%, signal=23%
REACTOME_DEUBIQUITINATION	241	0.36369365	1.3870909	0.004188482	0.18161857	1	2220	tags=27%, list=18%, signal=33%
WP METHIONINE DE NOVO AND SALVAGE PATHWAY	19	0.619485	1.6413921	0.004273505	0.031974137	1	1341	tags=32%, list=11%, signal=35%
KEGG_STEROID_BIOSYNTHESIS	15	0.71497184	1.7528673	0.004285714	0.010659319	0.873	1915	tags=60%, list=15%, signal=71%
REACTOME_GOLGI_TO_ER_RETROGRADE_TRANSPORT	114	0.43065467	1.5432135	0.004535148	0.06716419	1	2954	tags=34%, list=24%, signal=44%
REACTOME_HCMV_EARLY_EVENTS	78	0.4487983	1.5227745	0.004744959	0.077081	1	3311	tags=45%, list=26%, signal=61%
KEGG CARDIAC MUSCLE CONTRACTION	51	0.5383666	1.7096443	0.004884005	0.016360171	0.982	949	tags=29%, list=8%, signal=32%
REACTOME_ACTIVATION_OF_AMPK_DOWNSTREAM_OF_NMDARS	18	0.63921636	1.6671838	0.005657709	0.02504948	0.998	1715	tags=39%, list=14%, signal=45%
REACTOME_ABORTIVE_ELONGATION_OF_HIV_1_TRANSCRIPT_IN_THE_ABSENCE_OF_TAT	23	0.64154357	1.8080901	0.005722461	0.005614325	0.615	1952	tags=57%, list=16%, signal=67%
REACTOME SIGNALING BY FGFR2 IIIA TM	17	0.6961323	1.7942082	0.005865103	0.006467779	0.699	1935	tags=65%, list=15%, signal=76%
KEGG_ANTIGEN_PROCESSING_AND_PRESENTATION	44	0.5183292	1.6102449	0.00617284	0.040535554	1	615	tags=20%, list=5%, signal=21%
REACTOME_TRANSLATION_OF_SARS_COV_2_STRUCTURAL_PROTEINS	43	0.5247951	1.6243082	0.006570302	0.035943206	1	1325	tags=28%, list=11%, signal=31%
REACTOME_CARGO_CONCENTRATION_IN_THE_ER	27	0.5836051	1.6836497	0.006738544	0.021436786	0.997	579	tags=33%, list=5%, signal=35%
REACTOME MRNA DECAY BY 3 TO 5 EXORIBONUCLEASE	15	0.70878077	1.7694252	0.007032349	0.008660119	0.818	2614	tags=80%, list=21%, signal=101%
REACTOME_TRANSCRIPTION_OF_THE_HIV_GENOME	67	0.4637074	1.5395869	0.007194245	0.069073275	1	4215	tags=60%, list=34%, signal=89%
HALLMARK_FATTY_ACID_METABOLISM	135	0.39813825	1.4647282	0.0078125	0.11602194	1	2284	tags=30%, list=18%, signal=36%
WP UREA CYCLE AND METABOLISM OF AMINO GROUPS	17	0.66723025	1.7152798	0.008510638	0.015681388	0.974	2076	tags=47%, list=17%, signal=56%
REACTOME_RNA_POLYMERASE_II_TRANSCRIPTION_TERMINATION	65	0.47042662	1.5645106	0.008536586	0.057172205	1	1285	tags=28%, list=10%, signal=31%
REACTOME_BETA_CATENIN_INDEPENDENT_WNT_SIGNALING	131	0.39719635	1.4487723	0.009080591	0.1281964	1	1441	tags=27%, list=11%, signal=31%
KEGG_AMINOACYL_TRNA_BIOSYNTHESIS	41	0.51690483	1.6105089	0.009114583	0.04058097	1	2945	tags=59%, list=23%, signal=76%
REACTOME HIV TRANSCRIPTION ELONGATION	41	0.50400937	1.5517597	0.009150327	0.06332636	1	2063	tags=44%, list=16%, signal=52%
REACTOME_PROCESSING_OF_CAPPED_INTRONLESS_PRE_MRNA	28	0.56702554	1.6320015	0.009383378	0.033873074	1	1005	tags=29%, list=8%, signal=31%
REACTOME_COOPERATION_OF_PDCL_PHLP1_AND_TRIC_CCT_IN_G_PROTEIN_BETA_FOLDING	30	0.54262674	1.5724362	0.009395973	0.054983113	1	2362	tags=47%, list=19%, signal=57%
WP 7Q1123 COPY NUMBER VARIATION SYNDROME	77	0.45555872	1.5698205	0.009478673	0.05586843	1	2981	tags=38%, list=24%, signal=49%
REACTOME MATURATION OF SARS COV 2 SPIKE PROTEIN	28	0.57757026	1.6609062	0.009708738	0.026412157	0.998	1325	tags=32%, list=11%, signal=36%

Supplemental Table 2: DOWN-REGULATED PATHWAYS: Veh-GMP vs NTB-GMP

Supplemental Table 2	SIZE	ES	NES	NOM p-val	FDR q-val	FWER p-val	RANK AT MAX	LEADING EDGE
WP_CYTOPLASMIC_RIBOSOMAL_PROTEINS	86	-0.6356747	-2.6628711	0	0	0	953	tags=47%, list=8%, signal=50%
REACTOME_EUKARYOTIC_TRANSLATION_ELONGATION	87	-0.5916132	-2.5886714	0	0	0	953	tags=48%, list=8%, signal=52%
KEGG_RIBOSOME	82	-0.6087495	-2.5706313	0	0	0	953	tags=48%, list=8%, signal=51%
KEGG_CELL_ADHESION_MOLECULES_CAMS	82	-0.5596961	-2.4477386	0	0	0	1798	tags=37%, list=14%, signal=42%
REACTOME_RHO_GTPASES_ACTIVATE_WASPS_AND_WAVES	33	-0.6639113	-2.4073036	0	0	0	2277	tags=61%, list=18%, signal=74%
WP_MICROGLIA_PATHOGEN_PHAGOCYTOSIS_PATHWAY	38	-0.6381843	-2.3633244	0	3.83E-04	0.001	1393	tags=45%, list=11%, signal=50%
REACTOME_SELENOAMINO_ACID_METABOLISM	108	-0.5225099	-2.28367	0	0.001228388	0.005	597	tags=35%, list=5%, signal=37%
REACTOME_NONSENSE_MEDIATED_DECAY_NMD	110	-0.5113985	-2.2953446	0	0.001264134	0.004	987	tags=37%, list=8%, signal=40%
REACTOME_RESPONSE_OF_EIF2AK4_GCN2_TO_AMINO_ACID_DEFICIENCY	95	-0.5286734	-2.2837327	0	0.001381936	0.005	597	tags=38%, list=5%, signal=39%
WP_TYROBP_CAUSAL_NETWORK	56	-0.5744286	-2.2560017	0	0.001759515	0.008	1540	tags=32%, list=12%, signal=36%
KEGG_LEUKOCYTE_TRANSENDOTHELIAL_MIGRATION	87	-0.5339407	-2.247743	0	0.001802713	0.009	1896	tags=38%, list=15%, signal=44%
WP_HYPOTHESIZED_PATHWAYS_IN_PATHOGENESIS_OF_CARDIOVASCULAR_DISEASE	18	-0.7167929	-2.2099051	0	0.003122322	0.019	1716	tags=50%, list=14%, signal=58%
REACTOME_PARASITE_INFECTION	55	-0.5679736	-2.211085	0	0.003204321	0.018	2277	tags=55%, list=18%, signal=66%
REACTOME_IMMUNOREGULATORY_INTERACTIONS_BETWEEN_A_LYMPHOID_AND_A_NON_LYMPHOID_CEL L	74	-0.5284655	-2.2132306	0	0.003471348	0.018	1843	tags=30%, list=15%, signal=35%
PID_INTEGRIN2_PATHWAY	20	-0.7088893	-2.170396	0	0.004788048	0.032	933	tags=30%, list=7%, signal=32%
REACTOME_RHO_GTPASES_ACTIVATE_CIT	16	-0.6908966	-2.0858572	0	0.011517677	0.078	2621	tags=63%, list=21%, signal=79%
REACTOME_EUKARYOTIC_TRANSLATION_INITIATION	113	-0.4607919	-2.0776503	0	0.011996503	0.087	953	tags=39%, list=8%, signal=42%
PID_ANGIOPOIETIN_RECEPTOR_PATHWAY	44	-0.5551338	-2.0718975	0	0.012403259	0.094	1822	tags=41%, list=15%, signal=48%
KEGG_NATURAL_KILLER_CELL_MEDIATED_CYTOTOXICITY	83	-0.4709666	-2.0562098	0	0.014060239	0.11	1877	tags=33%, list=15%, signal=38%
REACTOME_EPHA_MEDIATED_GROWTH_CONE_COLLAPSE	17	-0.6926135	-2.0361745	0	0.016821114	0.141	1686	tags=47%, list=13%, signal=54%
KEGG_LEISHMANIA_INFECTION	54	-0.5129687	-2.0286067	0	0.017246617	0.152	1502	tags=31%, list=12%, signal=36%
HALLMARK_KRAS_SIGNALING_UP	141	-0.4349033	-2.010423	0	0.021132294	0.19	2380	tags=35%, list=19%, signal=42%
REACTOME_CELLULAR_RESPONSE_TO_STARVATION	144	-0.4403055	-2.0011292	0	0.0225868	0.207	597	tags=28%, list=5%, signal=29%
REACTOME_RAC1_GTPASE_CYCLE	166	-0.4232104	-1.9587288	0	0.02551256	0.292	1925	tags=32%, list=15%, signal=37%
REACTOME_CELL_JUNCTION_ORGANIZATION	51	-0.5012863	-1.9613314	0	0.026684979	0.289	1798	tags=37%, list=14%, signal=43%
REACTOME_RHO_GTPASES_ACTIVATE_NADPH_OXIDASES	22	-0.6130453	-1.9709477	0	0.026870212	0.264	891	tags=45%, list=7%, signal=49%
REACTOME_CELL_CELL_COMMUNICATION	81	-0.483761	-1.9626946	0	0.027096467	0.284	1798	tags=36%, list=14%, signal=42%
PID_AVB3_OPN_PATHWAY	30	-0.5602773	-1.9646288	0	0.027150279	0.277	1896	tags=50%, list=15%, signal=59%
REACTOME_RHO_GTPASES_ACTIVATE_PKNS	46	-0.5228721	-1.9742213	0	0.027238676	0.26	832	tags=28%, list=7%, signal=30%
KEGG_REGULATION_OF_ACTIN_CYTOSKELETON	157	-0.4150401	-1.9400321	0	0.02851418	0.344	1951	tags=32%, list=16%, signal=38%
REACTOME_ACTIVATION_OF_THE_MRNA_UPON_BINDING_OF_THE_CAP_BINDING_COMPLEX_AND_EIFS_A ND_SUBSEQUENT_BINDING_TO_43S	57	-0.4885347	-1.9402846	0	0.02933799	0.343	777	tags=40%, list=6%, signal=43%
WP_B_CELL_RECEPTOR_SIGNALING_PATHWAY	96	-0.434862	-1.9308797	0	0.030764207	0.375	1914	tags=36%, list=15%, signal=43%
REACTOME_LEISHMANIA_INFECTION	168	-0.3963751	-1.919275	0	0.031855028	0.404	2309	tags=36%, list=18%, signal=44%
WP_OVERVIEW_OF_LEUKOCYTEINTRINSIC_HIPPO_PATHWAY_FUNCTIONS	29	-0.5587735	-1.9008927	0	0.032937802	0.463	1828	tags=48%, list=15%, signal=56%
REACTOME_GPVI_MEDIATED_ACTIVATION_CASCADE	31	-0.5593969	-1.8976519	0	0.033029124	0.473	1896	tags=45%, list=15%, signal=53%
WP_CARDIAC_PROGENITOR_DIFFERENTIATION	20	-0.6195544	-1.9119093	0	0.03326732	0.43	2012	tags=45%, list=16%, signal=53%
PID_INTEGRIN_A9B1_PATHWAY	18	-0.6201932	-1.865211	0	0.033452537	0.584	1822	tags=50%, list=15%, signal=58%
REACTOME_G_ALPHA_S_SIGNALLING_EVENTS	69	-0.4451929	-1.8723654	0	0.033462543	0.548	1983	tags=33%, list=16%, signal=39%
PID_S1P_S1P1_PATHWAY	19	-0.6252094	-1.9033879	0	0.033625584	0.456	1822	tags=37%, list=15%, signal=43%
PID_RHOA_REG_PATHWAY	43	-0.5135266	-1.9074557	0	0.033695567	0.44	1435	tags=28%, list=11%, signal=31%
PID_CXCR4_PATHWAY	89	-0.446214	-1.904812	0	0.033775054	0.448	2276	tags=35%, list=18%, signal=42%
REACTOME_RUNX2_REGULATES_BONE_DEVELOPMENT	19	-0.599566	-1.8738056	0	0.033897977	0.546	1972	tags=42%, list=16%, signal=50%
PID_IL12_2PATHWAY	49	-0.4998959	-1.8779509	0	0.034651242	0.535	2118	tags=29%, list=17%, signal=34%
WP_PATHWAYS_AFFECTED_IN_ADENOID_CYSTIC_CARCINOMA	56	-0.4620461	-1.8806425	0	0.034657482	0.531	2514	tags=50%, list=20%, signal=62%
REACTOME_INTERFERON_GAMMA_SIGNALING	67	-0.4638548	-1.8838177	0	0.035134993	0.522	2208	tags=33%, list=18%, signal=40%
WP_AMPLIFICATION_AND_EXPANSION_OF_ONCOGENIC_PATHWAYS_AS_METASTATIC_TRAITS	15	-0.6569085	-1.8808645	0	0.035394873	0.531	1040	tags=33%, list=8%, signal=36%
WP_NEOVASCULARISATION_PROCESSES	32	-0.5357694	-1.8839741	0	0.03586806	0.521	1848	tags=31%, list=15%, signal=37%
REACTOME_AMYLOID_FIBER_FORMATION	54	-0.4775789	-1.8550936	0	0.036354486	0.629	632	tags=19%, list=5%, signal=19%
PID_FAK_PATHWAY	56	-0.4808391	-1.8488925	0	0.036556438	0.64	1822	tags=34%, list=15%, signal=40%
PID_IL8_CXCR2_PATHWAY	30	-0.5218402	-1.850761	0	0.03708617	0.635	1602	tags=47%, list=13%, signal=53%
WP_FOCAL_ADHESION	147	-0.3897624	-1.8339485	0	0.039956458	0.694	1943	tags=31%, list=15%, signal=37%
KEGG_FOCAL_ADHESION	144	-0.3939548	-1.8279878	0	0.040520586	0.709	1943	tags=32%, list=15%, signal=37%
KEGG_CHEMOKINE_SIGNALING_PATHWAY	138	-0.3877935	-1.8166724	0	0.040973607	0.753	2150	tags=36%, list=17%, signal=42%
REACTOME_REGULATION_OF_RUNX1_EXPRESSION_AND_ACTIVITY	17	-0.620077	-1.8281024	0	0.041110538	0.709	2176	tags=41%, list=17%, signal=50%
REACTOME_EPHB_MEDIATED_FORWARD_SIGNALING	37	-0.5021916	-1.8185775	0	0.041216265	0.742	2784	tags=57%, list=22%, signal=73%
PID_THROMBIN_PAR1_PATHWAY	38	-0.5024061	-1.8076899	0	0.04249089	0.773	2094	tags=47%, list=17%, signal=57%
WP_GASTRIN_SIGNALING_PATHWAY	96	-0.4117529	-1.8206095	0	0.042899895	0.735	2259	tags=36%, list=18%, signal=44%
REACTOME_OXIDATIVE_STRESS_INDUCED_SENESCENCE	77	-0.421521	-1.8026218	0	0.043094993	0.787	1592	tags=27%, list=13%, signal=31%
WP_CHEMOKINE_SIGNALING_PATHWAY	129	-0.3960187	-1.8035889	0	0.04326609	0.786	2150	tags=36%, list=17%, signal=43%
REACTOME_NEUTROPHIL_DEGRANULATION	413	-0.3474358	-1.7977573	0	0.04418112	0.803	926	tags=21%, list=7%, signal=22%
WP_PATHWAYS_REGULATING_HIPPO_SIGNALING	65	-0.439821	-1.7848631	0	0.047317054	0.834	2680	tags=46%, list=21%, signal=58%
PID_FCER1_PATHWAY	59	-0.432083	-1.7823021	0	0.04776456	0.841	1914	tags=29%, list=15%, signal=34%
WP_ALLOGRAFT_REJECTION	48	-0.4676011	-1.766786	0	0.05063309	0.874	2118	tags=33%, list=17%, signal=40%
REACTOME_CDC42_GTPASE_CYCLE	140	-0.3807606	-1.7668298	0	0.05120847	0.874	1925	tags=28%, list=15%, signal=33%
REACTOME_SRP_DEPENDENT_COTRANSLATIONAL_PROTEIN_TARGETING_TO_MEMBRANE	107	-0.3967248	-1.7584409	0	0.05315618	0.9	597	tags=34%, list=5%, signal=35%
REACTOME_TRANSCRIPTIONAL_REGULATION_OF_GRANULOPOIESIS	46	-0.465538	-1.7570721	0	0.0533771	0.903	353	tags=20%, list=3%, signal=20%
REACTOME_SIGNALING_BY_ROBO_RECEPTORS	193	-0.3597848	-1.7510984	0	0.05534948	0.917	1072	tags=28%, list=9%, signal=30%
REACTOME_REGULATION_OF_EXPRESSION_OF_SLITS_AND_ROBOS	158	-0.3647939	-1.7362156	0	0.05983512	0.932	614	tags=25%, list=5%, signal=26%
PID_P38_MK2_PATHWAY	18	-0.5850942	-1.7365613	0	0.060317203	0.931	1808	tags=39%, list=14%, signal=45%
PID_TXA2PATHWAY	50	-0.4465023	-1.732968	0	0.060690243	0.936	1822	tags=36%, list=15%, signal=42%
REACTOME_G_ALPHA_12_13_SIGNALLING_EVENTS	68	-0.4191406	-1.7012852	0	0.0726813	0.97	1877	tags=35%, list=15%, signal=41%
REACTOME_RHO_GTPASE_EFFECTORS	249	-0.3406477	-1.6965557	0	0.074809164	0.974	1896	tags=29%, list=15%, signal=33%
KEGG_VASCULAR_SMOOTH_MUSCLE_CONTRACTION	77	-0.3997588	-1.6831373	0	0.076973855	0.977	2309	tags=38%, list=18%, signal=46%
HALLMARK_COMPLEMENT	162	-0.3519872	-1.6892323	0	0.07883058	0.975	1478	tags=25%, list=12%, signal=28%
PID_TCR_PATHWAY	58	-0.4291067	-1.6660864	0	0.0816607	0.987	2045	tags=34%, list=16%, signal=41%
WP_REGULATION_OF_ACTIN_CYTOSKELETON	110	-0.3638985	-1.6542894	0	0.08659032	0.991	1951	tags=31%, list=16%, signal=36%
REACTOME_RUNX1_REGULATES_GENES_INVOLVED_IN_MEGAKARYOCYTE_DIFFERENTIATION_AND_PLATEL ET_FUNCTION	54	-0.4242021	-1.6525371	0	0.086906664	0.992	1676	tags=30%, list=13%, signal=34%
HALLMARK_APICAL_JUNCTION	145	-0.3576667	-1.6393747	0	0.092976995	0.994	2000	tags=30%, list=16%, signal=36%
HALLMARK_UV_RESPONSE_DN	117	-0.3635063	-1.6398375	0	0.093354926	0.994	1782	tags=28%, list=14%, signal=33%
SIG_BCR_SIGNALING_PATHWAY	46	-0.4256923	-1.6357418	0	0.0936943	0.995	2045	tags=35%, list=16%, signal=41%
NABA_SECRETED_FACTORS	135	-0.3553816	-1.6284075	0	0.09725081	0.996	3534	tags=44%, list=28%, signal=60%
HALLMARK_COAGULATION	100	-0.3693248	-1.6223503	0	0.09938717	0.997	763	tags=15%, list=6%, signal=16%
REACTOME_INFLUENZA_INFECTION	147	-0.3547135	-1.6197087	0	0.09971861	0.997	597	tags=24%, list=5%, signal=25%
REACTOME_RAC3_GTPASE_CYCLE	83	-0.3799808	-1.6120902	0	0.101434864	0.998	1896	tags=33%, list=15%, signal=38%
WP_EBOLA_VIRUS_PATHWAY_ON_HOST	105	-0.3621469	-1.6135107	0	0.101793006	0.998	1593	tags=29%, list=13%, signal=32%
REACTOME_SENSORY_PROCESSING_OF_SOUND	47	-0.4123937	-1.5908673	0	0.11211319	0.999	1726	tags=30%, list=14%, signal=34%
NABA_ECM_AFFILIATED	76	-0.3734776	-1.5883559	0	0.113324605	0.999	1478	tags=21%, list=12%, signal=24%
WP_TCELL_ANTIGEN_RECEPTOR_TCR_SIGNALING_PATHWAY	81	-0.367527	-1.5692992	0	0.11910759	0.999	2246	tags=31%, list=18%, signal=37%
NABA_MATRISOME_ASSOCIATED	329	-0.3070591	-1.5705633	0	0.1195852	0.999	1668	tags=18%, list=13%, signal=20%
REACTOME_NERVOUS_SYSTEM_DEVELOPMENT	432	-0.2896427	-1.5518202	0	0.12900971	0.999	1010	tags=19%, list=8%, signal=19%
REACTOME_MUSCLE_CONTRACTION	103	-0.3555525	-1.5374058	0	0.1335681	0.999	1633	tags=23%, list=13%, signal=27%
HALLMARK_MITOTIC_SPINDLE	194	-0.3116233	-1.5264663	0	0.13797341	0.999	1981	tags=28%, list=16%, signal=33%
KEGG_ADHERENS_JUNCTION	63	-0.3737854	-1.5181389	0	0.14257477	1	1896	tags=29%, list=15%, signal=33%
REACTOME_RHOA_GTPASE_CYCLE	135	-0.32283	-1.479176	0	0.16207375	1	1877	tags=27%, list=15%, signal=32%
REACTOME_SIGNALING_BY_GPCR	362	-0.2943064	-1.4811884	0	0.16232137	1	1983	tags=23%, list=16%, signal=26%
REACTOME_PLATELET_ACTIVATION_SIGNALING_AND_AGGREGATION	209	-0.2969655	-1.4742596	0	0.16495562	1	2095	tags=31%, list=17%, signal=36%
REACTOME_RHO_GTPASE_CYCLE	401	-0.276032	-1.4704717	0	0.16790332	1	1931	tags=27%, list=15%, signal=31%
REACTOME_HEMOSTASIS	464	-0.2724687	-1.3981988	0	0.20996867	1	1505	tags=21%, list=12%, signal=23%
HALLMARK_INTERFERON_GAMMA_RESPONSE	175	-0.2886368	-1.3882804	0	0.21689731	1	1539	tags=20%, list=12%, signal=22%
HALLMARK_ALLOGRAFT_REJECTION	158	-0.2953538	-1.3700353	0	0.22544642	1	2287	tags=34%, list=18%, signal=40%
KEGG_ALLOGRAFT_REJECTION	18	-0.6152093	-1.820371	0.003378379	0.042394813	0.735	3627	tags=72%, list=29%, signal=101%
PID_LYMPH_ANGIOGENESIS_PATHWAY	22	-0.571871	-1.8186715	0.003448276	0.042344637	0.742	2814	tags=50%, list=22%, signal=64%

BIOCARTA_NKCELLS_PATHWAY	16	-0.5981216	-1.7918458	0.003448276	0.045220923	0.816	1877	tags=50%, list=15%, signal=59%
REACTOME_RECOGNITION_AND_ASSOCIATION_OF_DNA_GLYCOSYLASE_WITH_SITE_CONTAINING_AN_AFFECTED_PURINE	23	-0.5923575	-1.8777517	0.003533569	0.034001477	0.535	325	tags=26%, list=3%, signal=27%
BIOCARTA_RHO_PATHWAY	20	-0.6060622	-1.8673548	0.003558719	0.033888534	0.573	1686	tags=40%, list=13%, signal=46%
PID_IL23_PATHWAY	27	-0.5759027	-1.927981	0.003636364	0.030564092	0.381	2118	tags=30%, list=17%, signal=36%
KEGG_ALDOSTERONE_REGULATED_SODIUM_REABSORPTION	32	-0.5000675	-1.7484124	0.003676471	0.05597382	0.918	1307	tags=34%, list=10%, signal=38%
KEGG_GRAFT_VERSUS_HOST_DISEASE	20	-0.616894	-1.9022758	0.003773585	0.033113487	0.459	3627	tags=75%, list=29%, signal=105%
KEGG_COMPLEMENT_AND_COAGULATION_CASCADES	32	-0.4998252	-1.7013073	0.003984064	0.07336698	0.97	1742	tags=28%, list=14%, signal=33%
WP_G13_SIGNALING_PATHWAY	35	-0.5439506	-1.961327	0.004048583	0.02579548	0.289	1896	tags=49%, list=15%, signal=57%
REACTOME_MEIOTIC_SYNAPSIS	41	-0.4978785	-1.8179376	0.004048583	0.04088271	0.744	2052	tags=39%, list=16%, signal=46%
PID_S1P_S1P3_PATHWAY	27	-0.5548864	-1.8665203	0.004065041	0.033634078	0.578	2096	tags=44%, list=17%, signal=53%
REACTOME_METABOLISM_OF_FAT_SOLUBLE_VITAMINS	32	-0.4813488	-1.6753806	0.00409836	0.07947694	0.983	909	tags=19%, list=7%, signal=20%
REACTOME_DAP12_SIGNALING	26	-0.5326906	-1.8345709	0.004115226	0.040210582	0.689	1154	tags=27%, list=9%, signal=30%
REACTOME_PRC2_METHYLATES_HISTONES_AND_DNA	31	-0.4888054	-1.7222244	0.004504505	0.063128814	0.95	1066	tags=26%, list=8%, signal=28%
PID_ENDOTHELIN_PATHWAY	50	-0.4460231	-1.7434663	0.004524887	0.057664182	0.922	2096	tags=42%, list=17%, signal=50%
PID_LYSOPHOSPHOLIPID_PATHWAY	54	-0.466568	-1.8501788	0.004587156	0.036686562	0.637	1822	tags=39%, list=15%, signal=45%
REACTOME_NRAGE_SIGNALS_DEATH_THROUGH_JNK	51	-0.4089876	-1.591217	0.004651163	0.112605415	0.999	1877	tags=33%, list=15%, signal=39%
WP_ASSOCIATION_BETWEEN_PHYSICOCHEMICAL_FEATURES_AND_TOXICITY_ASSOCIATED_PATHWAYS	49	-0.4408498	-1.6866995	0.004926108	0.07641382	0.976	1716	tags=29%, list=14%, signal=33%
PID_CDC42_PATHWAY	65	-0.3688195	-1.4836669	0.005181347	0.1613108	1	1896	tags=32%, list=15%, signal=38%
WP_RANKLRANK_RECEPTOR_ACTIVATOR_OF_NFKB_LIGAND_SIGNALING_PATHWAY	51	-0.4001474	-1.5128132	0.005208334	0.14515059	1	1896	tags=29%, list=15%, signal=34%
WP_HIPPOMERLIN_SIGNALING_DYSREGULATION	78	-0.3845018	-1.5809425	0.005649718	0.11602915	0.999	1874	tags=31%, list=15%, signal=36%
KEGG_FC_GAMMA_R_MEDIATED_PHAGOCYTOSIS	85	-0.3903973	-1.6875132	0.00621118	0.07749057	0.976	1896	tags=32%, list=15%, signal=37%
REACTOME_NOTCH3_INTRACELLULAR_DOMAIN_REGULATES_TRANSCRIPTION	17	-0.6233604	-1.8698134	0.006410257	0.033556845	0.557	2408	tags=53%, list=19%, signal=65%
BIOCARTA_CXCR4_PATHWAY	17	-0.607217	-1.8347864	0.006410257	0.040789012	0.688	852	tags=41%, list=7%, signal=44%
REACTOME_RAC2_GTPASE_CYCLE	81	-0.3511741	-1.5108578	0.006451613	0.1462009	1	1931	tags=35%, list=15%, signal=41%
BIOCARTA_HCMV_PATHWAY	17	-0.5759685	-1.7289473	0.006514658	0.060997706	0.941	1567	tags=41%, list=12%, signal=47%
BIOCARTA_EDG1_PATHWAY	19	-0.6011177	-1.8129612	0.006644518	0.041502036	0.761	852	tags=37%, list=7%, signal=39%
REACTOME_REPRODUCTION	80	-0.3583444	-1.5229753	0.006666667	0.13969018	0.999	874	tags=18%, list=7%, signal=19%
BIOCARTA_ECM_PATHWAY	18	-0.6574694	-1.9801838	0.006872852	0.026819956	0.246	1716	tags=61%, list=14%, signal=71%
REACTOME_DNA_METHYLATION	23	-0.5489921	-1.7812816	0.007142857	0.04742001	0.842	325	tags=22%, list=3%, signal=22%
HALLMARK_INTERFERON_ALPHA_RESPONSE	90	-0.3380743	-1.4751627	0.007194245	0.16473438	1	2444	tags=33%, list=19%, signal=41%
BIOCARTA_ALK_PATHWAY	21	-0.5986711	-1.8762118	0.007246377	0.033712137	0.539	2587	tags=57%, list=21%, signal=72%
PID_EPHA_FWDPATHWAY	26	-0.5298561	-1.7266566	0.007272727	0.061446946	0.946	1686	tags=38%, list=13%, signal=44%
REACTOME_ANTI_INFLAMMATORY_RESPONSE_FAVOURING_LEISHMANIA_PARASITE_INFECTION	89	-0.3235664	-1.3931392	0.007407407	0.21263762	1	1736	tags=24%, list=14%, signal=27%
REACTOME_DAP12_INTERACTIONS	32	-0.4874324	-1.7326633	0.007604563	0.060296215	0.936	1154	tags=25%, list=9%, signal=27%
PID_NECTIN_PATHWAY	28	-0.5307744	-1.7723709	0.007782101	0.04929985	0.86	1896	tags=50%, list=15%, signal=59%
BIOCARTA_FCER1_PATHWAY	38	-0.4625698	-1.6716373	0.007905139	0.08081885	0.983	1047	tags=24%, list=8%, signal=26%
PID_RHOA_PATHWAY	39	-0.4500875	-1.6497809	0.008097166	0.08783223	0.994	1896	tags=36%, list=15%, signal=42%
REACTOME_NR1H3_NR1H2_REGULATE_GENE_EXPRESSION_LINKED_TO_CHOLESTEROL_TRANSPORT_AND_EFFLUX	33	-0.4864161	-1.7309166	0.008130081	0.060486034	0.937	2971	tags=48%, list=24%, signal=63%
WP_MIRNAS_INVOLVEMENT_IN_THE_IMMUNE_RESPONSE_IN_SEPSIS	34	-0.471732	-1.6867169	0.008230452	0.07709005	0.976	1970	tags=26%, list=16%, signal=31%
KEGG_LYSOSOME	113	-0.3216741	-1.434063	0.008264462	0.1891531	1	1672	tags=25%, list=13%, signal=28%
REACTOME_BASE_EXCISION_REPAIR_AP_SITE_FORMATION	29	-0.5158156	-1.7743063	0.008403362	0.04931947	0.858	325	tags=21%, list=3%, signal=21%
REACTOME_COMPLEMENT_CASCADE	28	-0.5335121	-1.7658477	0.008583691	0.05040469	0.875	1184	tags=25%, list=9%, signal=28%
REACTOME_INTEGRIN_CELL_SURFACE_INTERACTIONS	47	-0.4000577	-1.5330861	0.009009009	0.13580261	0.999	2744	tags=36%, list=22%, signal=46%
PID_CD8_TCR_PATHWAY	47	-0.453152	-1.7569346	0.009174312	0.05287171	0.905	1877	tags=32%, list=15%, signal=37%
PID_RAC1_REG_PATHWAY	37	-0.4548821	-1.6687208	0.009259259	0.08211297	0.984	2495	tags=49%, list=20%, signal=61%
PID_RAC1_PATHWAY	50	-0.4356112	-1.6599369	0.009259259	0.08423297	0.99	1161	tags=26%, list=9%, signal=29%
KEGG_SYSTEMIC_LUPUS_ERYTHEMATOSUS	55	-0.4041131	-1.5813612	0.009259259	0.11646582	0.999	325	tags=15%, list=3%, signal=15%
WP_MYOMETRIAL_RELAXATION_AND_CONTRACTION_PATHWAYS	118	-0.3139822	-1.4128925	0.009259259	0.20059311	1	1395	tags=22%, list=11%, signal=25%
WP_FBXL10_ENHANCEMENT_OF_MAPERK_SIGNALING_IN_DIFFUSE_LARGE_BCELL_LYMPHOMA	22	-0.5747356	-1.8078697	0.009677419	0.042998686	0.773	997	tags=36%, list=8%, signal=39%
BIOCARTA_BAD_PATHWAY	21	-0.5442106	-1.66848	0.009933775	0.08160095	0.984	852	tags=38%, list=7%, signal=41%

Supplemental Table 3: UP-REGULATED PATHWAYS: BRQ-GMP vs Veh-GMP

Supplemental Table 3	SIZE	ES	NES	NOM p-val	FDR q-val	FWER p-val	RANK AT MAX	LEADING EDGE
WP_COMMON_PATHWAYS_UNDERLYING_DRUG_ADDICTION	26	0.81065077	1.9727614	0	0	0	851	tags=58%, list=7%, signal=62%
KEGG_REGULATION_OF_ACTIN_CYTOSKELETON	168	0.63670367	1.9328203	0	5.19E-04	0.002	2163	tags=40%, list=17%, signal=48%
PID_P38_MK2_PATHWAY	19	0.8245085	1.8965377	0	5.20E-04	0.005	1524	tags=63%, list=12%, signal=71%
REACTOME_PARASITE_INFECTION	55	0.72635055	1.9609019	0	5.22E-04	0.001	2069	tags=56%, list=16%, signal=67%
REACTOME_RHO_GTPASES_ACTIVATE_WASPS_AND_WAVES	33	0.7503619	1.8878669	0	5.59E-04	0.007	2069	tags=67%, list=16%, signal=79%
WP_PROSTAGLANDIN_SYNTHESIS_AND_REGULATION	33	0.750492	1.8997966	0	5.78E-04	0.005	1068	tags=36%, list=8%, signal=40%
KEGG_FC_GAMMA_R_MEDIATED_PHAGOCYTOSIS	87	0.661198	1.889252	0	6.05E-04	0.007	2534	tags=51%, list=19%, signal=62%
WP_REGULATION_OF_ACTIN_CYTOSKELETON	117	0.6444283	1.9206372	0	6.26E-04	0.003	2163	tags=41%, list=17%, signal=49%
PID_CDC42_PATHWAY	67	0.6802077	1.9073204	0	6.50E-04	0.005	2269	tags=51%, list=17%, signal=61%
REACTOME_REGULATION_OF_RUNX1_EXPRESSION_AND_ACTIVITY	17	0.83215296	1.8899868	0	6.61E-04	0.007	1172	tags=53%, list=9%, signal=58%
WP_G13_SIGNALING_PATHWAY	36	0.7596152	1.9507217	0	6.91E-04	0.002	1816	tags=61%, list=14%, signal=71%
REACTOME_G_BETA_GAMMA_SIGNALLING_THROUGH_CDC42	17	0.83668107	1.880496	0	7.40E-04	0.01	889	tags=53%, list=7%, signal=57%
REACTOME_EPHB_MEDIATED_FORWARD_SIGNALING	36	0.7398396	1.9088732	0	7.43E-04	0.005	1415	tags=50%, list=11%, signal=56%
REACTOME_RHO_GTPASE_EFFECTORS	255	0.6038579	1.8744537	0	7.76E-04	0.012	2134	tags=39%, list=16%, signal=46%
PID_CXCR4_PATHWAY	90	0.6481325	1.8758818	0	8.28E-04	0.012	2565	tags=48%, list=20%, signal=59%
REACTOME_SIGNAL_AMPLIFICATION	28	0.77738315	1.91918	0	8.67E-04	0.005	1669	tags=46%, list=13%, signal=53%
KEGG_CHEMOKINE_SIGNALING_PATHWAY	144	0.61991847	1.8633664	0	0.00121185	0.021	2474	tags=43%, list=19%, signal=53%
PID_RAC1_PATHWAY	51	0.69863033	1.8635832	0	0.00128313	0.021	2232	tags=57%, list=17%, signal=68%
KEGG_LEUKOCYTE_TRANSENDOTHELIAL_MIGRATION	92	0.64766276	1.8619411	0	0.00131369	0.024	2037	tags=42%, list=16%, signal=50%
WP_TYROBP_CAUSAL_NETWORK	55	0.6866769	1.8564713	0	0.00138605	0.028	2022	tags=42%, list=16%, signal=49%
HALLMARK_MITOTIC_SPINDLE	195	0.6039782	1.8569818	0	0.00140331	0.027	3284	tags=51%, list=25%, signal=67%
REACTOME_ADP_SIGNALLING_THROUGH_P2Y_PURINOCEPTOR_12	17	0.81730044	1.8539032	0	0.00141697	0.03	1669	tags=53%, list=13%, signal=61%
WP_PATHWAYS_AFFECTED_IN_ADENOID_CYSTIC_CARCINOMA	57	0.67557037	1.8444862	0	0.00176127	0.038	2612	tags=63%, list=20%, signal=79%
WP_CHEMOKINE_SIGNALING_PATHWAY	135	0.60914785	1.8348538	0	0.00181588	0.046	2722	tags=44%, list=21%, signal=56%
REACTOME_G_PROTEIN_BETA_GAMMA_SIGNALLING	29	0.74534976	1.8350738	0	0.00188313	0.046	1669	tags=48%, list=13%, signal=55%
WP_ASSOCIATION_BETWEEN_PHYSICOCHEMICAL_FEATURES_AND_TOXICITY_ASSOCIATED_PATHWAYS	53	0.68115884	1.8389337	0	0.00190924	0.043	855	tags=30%, list=7%, signal=32%
REACTOME_ADP_SIGNALLING_THROUGH_P2Y_PURINOCEPTOR_1	22	0.77569973	1.8363178	0	0.00195556	0.046	889	tags=41%, list=7%, signal=44%
SIG_INSULIN_RECEPTOR_PATHWAY_IN_CARDIAC_MYOCYTES	45	0.7037069	1.8393089	0	0.00198879	0.043	1954	tags=60%, list=15%, signal=70%
REACTOME_ADRENALINE_NORADRENALINE_INHIBITS_INSULIN_SECRETION	22	0.7757345	1.8298024	0	0.0020403	0.056	889	tags=32%, list=7%, signal=34%
BIOCARTA_BAD_PATHWAY	22	0.7665096	1.8240392	0	0.00207512	0.059	2345	tags=73%, list=18%, signal=89%
WP_EBOLA_VIRUS_PATHWAY_ON_HOST	107	0.6228917	1.8307049	0	0.00207555	0.055	2625	tags=44%, list=20%, signal=55%
BIOCARTA_NKCELLS_PATHWAY	16	0.80947644	1.8190242	0	0.00236884	0.07	2037	tags=75%, list=16%, signal=89%
PID_AVB3_OPN_PATHWAY	30	0.72846943	1.8127996	0	0.00252265	0.08	2372	tags=63%, list=18%, signal=77%
KEGG_NATURAL_KILLER_CELL_MEDIATED_CYTOTOXICITY	86	0.6323783	1.8131169	0	0.00256635	0.08	2290	tags=40%, list=18%, signal=48%
REACTOME_REGULATION_OF_INSULIN_SECRETION	60	0.66001624	1.8139583	0	0.0026128	0.079	1669	tags=33%, list=13%, signal=38%
WP_HAIR_FOLLICLE_DEVELOPMENT_ORGANOGENESIS_PART_2_OF_3	22	0.7668431	1.8075691	0	0.00266619	0.089	574	tags=27%, list=4%, signal=28%
PID_ECADHERIN_NASCENT_AJ_PATHWAY	39	0.70212525	1.8053659	0	0.00267837	0.092	1638	tags=49%, list=13%, signal=56%
WP_AGERAGE_PATHWAY	60	0.64734215	1.8077382	0	0.00274025	0.089	2066	tags=42%, list=16%, signal=49%
BIOCARTA_HCMV_PATHWAY	17	0.80028105	1.8010501	0	0.00282306	0.1	2345	tags=71%, list=18%, signal=86%
REACTOME_LEISHMANIA_INFECTION	170	0.58866566	1.800695	0	0.00283025	0.103	2274	tags=36%, list=17%, signal=43%
REACTOME_GLUCAGON_LIKE_PEPTIDE_1_GLP1_REGULATES_INSULIN_SECRETION	33	0.72561556	1.7995343	0	0.00283794	0.106	2262	tags=52%, list=17%, signal=62%
KEGG_CELL_CYCLE	116	0.6072825	1.7957417	0	0.00284498	0.109	3259	tags=53%, list=25%, signal=71%
REACTOME_THROMBOXANE_SIGNALLING_THROUGH_TP_RECEPTOR	21	0.76044047	1.7891649	0	0.00311915	0.131	889	tags=38%, list=7%, signal=41%
KEGG_FOCAL_ADHESION	157	0.5844163	1.7896686	0	0.00314144	0.129	2372	tags=36%, list=18%, signal=44%
REACTOME_FACTORS_INVOLVED_IN_MEGAKARYOCYTE_DEVELOPMENT_AND_PLATELET_PRODUCTION	121	0.6063729	1.7906796	0	0.00314182	0.127	2033	tags=36%, list=16%, signal=43%
PID_THROMBIN_PAR1_PATHWAY	41	0.70271915	1.791266	0	0.00314185	0.124	2796	tags=61%, list=21%, signal=77%
PID_NFAT_3PATHWAY	52	0.66176486	1.7917079	0	0.00321492	0.124	2534	tags=50%, list=19%, signal=62%
REACTOME_RHO_GTPASES_ACTIVATE_PKNS	46	0.6727107	1.7859771	0	0.0032491	0.138	1195	tags=39%, list=9%, signal=43%
REACTOME_VASOPRESSIN_REGULATES_RENAL_WATER_HOMEOSTASIS_VIA_AQUAPORINS	32	0.710793	1.7847044	0	0.00328966	0.141	2262	tags=44%, list=17%, signal=53%
WP_MICROGLIA_PATHOGEN_PHAGOCYTOSIS_PATHWAY	39	0.6844593	1.7817589	0	0.00343123	0.149	2274	tags=49%, list=17%, signal=59%
WP_HEMATOPOIETIC_STEM_CELL_DIFFERENTIATION	44	0.6750372	1.7777528	0	0.00371063	0.166	2341	tags=43%, list=18%, signal=52%
WP_CELL_CYCLE	112	0.5997614	1.7749974	0	0.00384007	0.175	3259	tags=52%, list=25%, signal=68%
REACTOME_RAC2_GTPASE_CYCLE	82	0.62210375	1.7732887	0	0.00385247	0.181	2134	tags=46%, list=16%, signal=55%
WP_REGULATION_OF_MICROTUBULE_CYTOSKELETON	42	0.672087	1.7697937	0	0.00414066	0.198	1247	tags=43%, list=10%, signal=47%
WP_IL3_SIGNALING_PATHWAY	46	0.6615391	1.7686287	0	0.00419694	0.204	2345	tags=54%, list=18%, signal=66%
BIOCARTA_MET_PATHWAY	32	0.69624364	1.7630765	0	0.00453033	0.23	2279	tags=59%, list=18%, signal=72%
REACTOME_GPVI_MEDIATED_ACTIVATION_CASCADE	32	0.70496184	1.7611839	0	0.00455185	0.237	2037	tags=47%, list=16%, signal=55%
REACTOME_G_ALPHA_12_13_SIGNALLING_EVENTS	70	0.6358896	1.7636583	0	0.00455433	0.227	2131	tags=39%, list=16%, signal=46%
WP_FOCAL_ADHESION	160	0.57568216	1.7596508	0	0.00456239	0.241	2372	tags=36%, list=18%, signal=43%
REACTOME_FCGAMMA_RECEPTOR_FCGR_DEPENDENT_PHAGOCYTOSIS	81	0.6188877	1.7586341	0	0.00457308	0.245	2274	tags=53%, list=17%, signal=64%
KEGG_OOCYTE_MEIOSIS	94	0.60940135	1.761665	0	0.00459334	0.236	2684	tags=45%, list=21%, signal=56%
WP_B_CELL_RECEPTOR_SIGNALING_PATHWAY	96	0.60709965	1.7530646	0	0.00460674	0.269	2345	tags=45%, list=18%, signal=54%
REACTOME_TRANSCRIPTIONAL_REGULATION_OF_GRANULOPOIESIS	48	0.6596083	1.7638613	0	0.00461617	0.226	2036	tags=48%, list=16%, signal=57%
KEGG_VIRAL_MYOCARDITIS	42	0.6648798	1.7532531	0	0.00465842	0.268	1331	tags=26%, list=10%, signal=29%
REACTOME_THROMBIN_SIGNALLING_THROUGH_PROTEINASE_ACTIVATED_RECEPTORS_PARS	29	0.6976423	1.7545855	0	0.00467303	0.261	889	tags=31%, list=7%, signal=33%
KEGG_LEISHMANIA_INFECTION	57	0.64386415	1.7536697	0	0.0047115	0.267	1954	tags=39%, list=15%, signal=45%
BIOCARTA_ECM_PATHWAY	19	0.7557613	1.7557126	0	0.00472104	0.256	773	tags=53%, list=6%, signal=56%
PID_ANGIOPOIETIN_RECEPTOR_PATHWAY	46	0.6596978	1.7503483	0	0.00473836	0.279	2458	tags=52%, list=19%, signal=64%
BIOCARTA_MCALPAIN_PATHWAY	16	0.78360134	1.7470988	0	0.00474124	0.296	1481	tags=50%, list=11%, signal=56%
WP_FBXL10_ENHANCEMENT_OF_MAPERK_SIGNALING_IN_DIFFUSE_LARGE_BCELL_LYMPHOMA	22	0.7536309	1.754643	0	0.00474383	0.261	1154	tags=45%, list=9%, signal=50%
BIOCARTA_CREB_PATHWAY	20	0.75109905	1.750835	0	0.00476109	0.277	2345	tags=75%, list=18%, signal=91%
REACTOME_EPIGENETIC_REGULATION_OF_GENE_EXPRESSION	105	0.5948596	1.7473212	0	0.00478983	0.296	2989	tags=50%, list=23%, signal=64%
PID_MET_PATHWAY	76	0.61925846	1.7475679	0	0.00482604	0.294	2458	tags=43%, list=19%, signal=53%
REACTOME_EPH_EPHRIN_SIGNALING	76	0.61894315	1.7483633	0	0.00484517	0.288	2598	tags=43%, list=20%, signal=54%
REACTOME_RAC1_GTPASE_CYCLE	166	0.57690907	1.7475736	0	0.00489125	0.294	2569	tags=41%, list=20%, signal=50%
REACTOME_CELL_CELL_COMMUNICATION	85	0.6051496	1.7428404	0	0.00489194	0.316	2458	tags=38%, list=19%, signal=46%
KEGG_NEUROTROPHIN_SIGNALING_PATHWAY	108	0.59231603	1.7404014	0	0.00501401	0.329	2345	tags=43%, list=18%, signal=52%
REACTOME_G_BETA_GAMMA_SIGNALLING_THROUGH_PI3KGAMMA	22	0.74188894	1.7404861	0	0.00506197	0.329	889	tags=36%, list=7%, signal=39%
WP_G_PROTEIN_SIGNALING_PATHWAYS	78	0.609875	1.7407081	0	0.00511125	0.329	2262	tags=36%, list=17%, signal=43%
PID_PDGFRB_PATHWAY	123	0.5863805	1.7377735	0	0.00511484	0.336	2565	tags=46%, list=20%, signal=56%
REACTOME_OPIOID_SIGNALLING	77	0.6167826	1.7368072	0	0.00514017	0.343	2290	tags=40%, list=18%, signal=49%
WP_PANCREATIC_ADENOCARCINOMA_PATHWAY	81	0.6031436	1.7361058	0	0.00517582	0.347	2345	tags=41%, list=18%, signal=49%
WP_MYOMETRIAL_RELAXATION_AND_CONTRACTION_PATHWAYS	120	0.5763357	1.7331269	0	0.0055149	0.366	2262	tags=34%, list=17%, signal=41%
REACTOME_MAP2K_AND_MAPK_ACTIVATION	34	0.6858121	1.7305604	0	0.00555678	0.385	2269	tags=53%, list=17%, signal=64%
BIOCARTA_IGF1R_PATHWAY	20	0.7447652	1.7310677	0	0.00556084	0.38	2345	tags=70%, list=18%, signal=85%

Supplemental Table 3: UP-REGULATED PATHWAYS: BRQ-GMP vs Veh-GMP

REACTOME_TP53_REGULATES_METABOLIC_GENES	84	0.6034366	1.7288115	0	0.00558183	0.392	2345	tags=43%, list=18%, signal=52%
PID_MTOR_4PATHWAY	66	0.6246586	1.7281826	0	0.00558871	0.396	3139	tags=56%, list=24%, signal=74%
REACTOME_PRESYNAPTIC_FUNCTION_OF_KAINATE_RECEPTORS	17	0.7653318	1.7315032	0	0.00559913	0.379	1669	tags=47%, list=13%, signal=54%
REACTOME_AQUAPORIN_MEDIATED_TRANSPORT	35	0.68044347	1.7262831	0	0.0057075	0.409	2262	tags=40%, list=17%, signal=48%
WP_EICOSANOID_SYNTHESIS	19	0.75010526	1.7249207	0	0.00572782	0.418	1025	tags=37%, list=8%, signal=40%
WP_MICRORNAS_IN_CARDIOMYOCYTE_HYPERTROPHY	70	0.613517	1.7250708	0	0.0057754	0.417	2541	tags=44%, list=20%, signal=55%
REACTOME_GLUCAGON_TYPE_LIGAND_RECEPTORS	17	0.765435	1.7228396	0	0.00578276	0.431	889	tags=41%, list=7%, signal=44%
PID_HES_HEY_PATHWAY	37	0.66993403	1.7241415	0	0.00578415	0.424	1155	tags=35%, list=9%, signal=38%
WP_WNT_SIGNALING_PATHWAY_NETPATH	47	0.6469604	1.719643	0	0.0059813	0.448	2519	tags=40%, list=19%, signal=50%
SIG_CHEMOTAXIS	39	0.6601966	1.7176332	0	0.00615939	0.46	2535	tags=56%, list=19%, signal=70%
REACTOME_SIGNALING_BY_HIPPO	20	0.74761075	1.717966	0	0.0061877	0.458	606	tags=25%, list=5%, signal=26%
WP_MRNA_PROCESSING	125	0.56930774	1.712146	0	0.00662914	0.505	2467	tags=46%, list=19%, signal=56%
REACTOME_SENSORY_PROCESSING_OF_SOUND	53	0.63050157	1.7122904	0	0.00665129	0.504	2392	tags=36%, list=18%, signal=44%
REACTOME_REGULATION_OF_PTEN_GENE_TRANSCRIPTION	57	0.6275238	1.7109288	0	0.00665876	0.513	3139	tags=60%, list=24%, signal=78%
BIOCARTA_RAS_PATHWAY	22	0.73274046	1.7111905	0	0.00666213	0.509	2345	tags=59%, list=18%, signal=72%
PID_BMP_PATHWAY	27	0.7012417	1.7127532	0	0.00666837	0.496	1933	tags=52%, list=15%, signal=61%
WP_IL4_SIGNALING_PATHWAY	53	0.6385779	1.7100662	0	0.0066692	0.518	2565	tags=49%, list=20%, signal=61%
HALLMARK_TGF_BETA_SIGNALING	48	0.63869816	1.710108	0	0.0067173	0.518	1935	tags=42%, list=15%, signal=49%
BIOCARTA_CXCR4_PATHWAY	17	0.7536346	1.7134016	0	0.0067335	0.495	1989	tags=65%, list=15%, signal=76%
PID_RB_1PATHWAY	56	0.6300701	1.7088257	0	0.00684139	0.531	3393	tags=59%, list=26%, signal=79%
REACTOME_MRNA_SPLICING	185	0.55428135	1.704494	0	0.00735473	0.561	2377	tags=42%, list=18%, signal=51%
KEGG_HUNTINGTONS_DISEASE	157	0.558902	1.7038329	0	0.00738052	0.564	2865	tags=43%, list=22%, signal=54%
WP_INTEGRATED_BREAST_CANCER_PATHWAY	139	0.56347454	1.6999961	0	0.00752345	0.591	3154	tags=45%, list=24%, signal=58%
WP_INTEGRINMEDIATED_CELL_ADHESION	87	0.58657324	1.7002448	0	0.00755834	0.59	2458	tags=39%, list=19%, signal=48%
REACTOME_ANTI_INFLAMMATORY_RESPONSE_FAVOURING_LEISHMANIA_PARASITE_INFECTION	91	0.5845143	1.6979967	0	0.00782142	0.612	2274	tags=31%, list=17%, signal=37%
REACTOME_PKMTS_METHYLATE_HISTONE_LYSINES	48	0.62545294	1.6971812	0	0.0079059	0.618	2983	tags=52%, list=23%, signal=67%
REACTOME_IMMUNOREGULATORY_INTERACTIONS_BETWEEN_A_LYMPHOID_AND_A_NON_LYMPHOID_CELL	80	0.6032207	1.696604	0	0.00791672	0.621	738	tags=18%, list=6%, signal=18%
PID_RAC1_REG_PATHWAY	37	0.6602252	1.6906914	0	0.00819392	0.665	2566	tags=54%, list=20%, signal=67%
REACTOME_GABA_B_RECEPTOR_ACTIVATION	28	0.69073415	1.6925164	0	0.00820386	0.65	1669	tags=32%, list=13%, signal=37%
SIG_BCR_SIGNALING_PATHWAY	46	0.6399395	1.6919208	0	0.00822012	0.653	2371	tags=57%, list=18%, signal=69%
WP_PDGF_PATHWAY	39	0.64460105	1.6936221	0	0.00822417	0.643	864	tags=36%, list=7%, signal=38%
PID_SYNDECAN_2_PATHWAY	28	0.6752302	1.6907717	0	0.00822964	0.663	2279	tags=50%, list=18%, signal=60%
WP_RETT_SYNDROME_CAUSING_GENES	36	0.6698397	1.688412	0	0.00826109	0.687	3040	tags=53%, list=23%, signal=69%
WP_GASTRIN_SIGNALING_PATHWAY	100	0.5814449	1.6925516	0	0.00826419	0.65	2534	tags=42%, list=19%, signal=52%
KEGG_SPLICEOSOME	122	0.56895703	1.691358	0	0.00829547	0.658	2174	tags=44%, list=17%, signal=53%
KEGG_CHRONIC_MYELOID_LEUKEMIA	67	0.60321176	1.6872389	0	0.00837405	0.691	3262	tags=52%, list=25%, signal=69%
BIOCARTA_VIP_PATHWAY	23	0.7041028	1.6831474	0	0.00893383	0.713	893	tags=39%, list=7%, signal=42%
PID_IL8_CXCR1_PATHWAY	27	0.69032264	1.680803	0	0.00923995	0.728	1138	tags=33%, list=9%, signal=36%
KEGG_SYSTEMIC_LUPUS_ERYTHEMATOSUS	58	0.6118648	1.679512	0	0.00942429	0.739	1374	tags=26%, list=11%, signal=29%
REACTOME_ARACHIDONIC_ACID_METABOLISM	38	0.644885	1.6777209	0	0.00974053	0.755	1025	tags=24%, list=8%, signal=26%
REACTOME_CDC42_GTPASE_CYCLE	143	0.557212	1.6759733	0	0.00990526	0.768	2162	tags=33%, list=17%, signal=39%
WP_SUDDEN_INFANT_DEATH_SYNDROME_SIDS_SUSCEPTIBILITY_PATHWAYS	96	0.5749205	1.6701456	0	0.01061714	0.805	2262	tags=43%, list=17%, signal=51%
PID_KIT_PATHWAY	50	0.6248792	1.667775	0	0.01069453	0.82	2939	tags=50%, list=23%, signal=64%
REACTOME_SIGNALING_BY_VEGF	99	0.5746346	1.6695306	0	0.01071014	0.808	2458	tags=44%, list=19%, signal=54%
KEGG_INSULIN_SIGNALING_PATHWAY	115	0.56580544	1.6686599	0	0.01073115	0.813	1954	tags=35%, list=15%, signal=41%
REACTOME_INTRINSIC_PATHWAY_FOR_APOPTOSIS	51	0.62488836	1.6663513	0	0.01076034	0.831	2345	tags=43%, list=18%, signal=52%
REACTOME_RUNX1_REGULATES_GENES_INVOLVED_IN_MEGAKARYOCYTE_DIFFERENTIATION_AND_PLATELET_FUNCTION	54	0.62091917	1.6680907	0	0.01076632	0.818	1172	tags=30%, list=9%, signal=32%
REACTOME_INTEGRATION_OF_ENERGY_METABOLISM	87	0.58226436	1.6665062	0	0.01079223	0.831	2262	tags=31%, list=17%, signal=37%
PID_HDAC_CLASSII_PATHWAY	33	0.6597422	1.6669824	0	0.01081472	0.826	1571	tags=39%, list=12%, signal=45%
REACTOME_CELL_JUNCTION_ORGANIZATION	55	0.6118481	1.6636631	0	0.01100706	0.847	1040	tags=24%, list=8%, signal=26%
REACTOME_G_ALPHA_I_SIGNALLING_EVENTS	169	0.54696125	1.6637896	0	0.01103365	0.846	2290	tags=26%, list=18%, signal=31%
REACTOME_NEUTROPHIL_DEGRANULATION	416	0.5218454	1.6646535	0	0.0110384	0.843	1757	tags=33%, list=14%, signal=37%
PID_IL8_CXCR2_PATHWAY	33	0.66866374	1.6638888	0	0.01112364	0.845	2362	tags=45%, list=18%, signal=55%
REACTOME_PROCESSING_OF_CAPPED_INTRON_CONTAINING_PRE_MRNA	236	0.53105617	1.6601871	0	0.01146574	0.863	2766	tags=42%, list=21%, signal=52%
PID_LIS1_PATHWAY	24	0.6901004	1.6586806	0	0.01163535	0.865	1142	tags=33%, list=9%, signal=36%
PID_ILK_PATHWAY	43	0.6377932	1.6581496	0	0.01170186	0.867	2458	tags=53%, list=19%, signal=66%
REACTOME_POSITIVE_EPIGENETIC_REGULATION_OF_RRNA_EXPRESSION	64	0.6026291	1.6566133	0	0.01200993	0.871	2989	tags=52%, list=23%, signal=67%
BIOCARTA_INTEGRIN_PATHWAY	31	0.6625712	1.6538285	0	0.0123267	0.885	2163	tags=52%, list=17%, signal=62%
REACTOME_PLATELET_ACTIVATION_SIGNALING_AND_AGGREGATION	218	0.53123885	1.6529593	0	0.01234541	0.89	2163	tags=37%, list=17%, signal=44%
WP_FACTORS_AND_PATHWAYS_AFFECTING_INSULINLIKE_GROWTH_FACTOR_IGF1AKT_SIGNALING	33	0.65075535	1.6531191	0	0.01237228	0.89	2666	tags=48%, list=20%, signal=61%
KEGG_CELL_ADHESION_MOLECULES_CAMS	85	0.57791954	1.6502594	0	0.012657	0.9	1971	tags=26%, list=15%, signal=30%
WP_EGFEGFR_SIGNALING_PATHWAY	152	0.54294145	1.6491177	0	0.01277038	0.906	2598	tags=40%, list=20%, signal=50%
HALLMARK_G2M_CHECKPOINT	193	0.5348426	1.6469444	0	0.01303049	0.923	2659	tags=45%, list=20%, signal=55%
REACTOME_GABA_RECEPTOR_ACTIVATION	31	0.6588519	1.6470529	0	0.01306969	0.923	2330	tags=35%, list=18%, signal=43%
KEGG_TIGHT_JUNCTION	95	0.56288934	1.6459825	0	0.01308557	0.927	3262	tags=41%, list=25%, signal=54%
KEGG_LONG_TERM_POTENTIATION	54	0.6150731	1.6436421	0	0.01335647	0.929	2722	tags=54%, list=21%, signal=68%
PID_FOXO_PATHWAY	45	0.62425727	1.6420901	0	0.01338436	0.932	3712	tags=64%, list=29%, signal=90%
REACTOME_HEMOSTASIS	480	0.5181846	1.642982	0	0.01341211	0.931	2059	tags=31%, list=16%, signal=36%
WP_SIGNALING_OF_HEPATOCYTE_GROWTH_FACTOR_RECEPTOR	34	0.6487172	1.6431112	0	0.01344661	0.931	2458	tags=53%, list=19%, signal=65%
REACTOME_MYOGENESIS	22	0.70125955	1.6399641	0	0.01359807	0.939	2008	tags=45%, list=15%, signal=54%
REACTOME_ONCOGENIC_MAPK_SIGNALING	74	0.5820543	1.6367216	0	0.01399913	0.948	3395	tags=51%, list=26%, signal=69%
WP_ALLOGRAFT_REJECTION	52	0.6057872	1.6368803	0	0.01402016	0.948	2066	tags=21%, list=16%, signal=25%
WP_MECP2_AND_ASSOCIATED_RETT_SYNDROME	55	0.6062363	1.6359589	0	0.01406869	0.95	2384	tags=51%, list=18%, signal=62%
PID_ERBB1_DOWNSTREAM_PATHWAY	103	0.56565094	1.6339498	0	0.01437093	0.957	2345	tags=39%, list=18%, signal=47%
PID_HDAC_CLASSI_PATHWAY	64	0.5918893	1.6314538	0	0.01474034	0.961	3185	tags=48%, list=24%, signal=64%
REACTOME_NEUROTRANSMITTER_RECEPTORS_AND_POSTSYNAPTIC_SIGNAL_TRANSMISSION	125	0.54705864	1.6304809	0	0.01492646	0.963	3110	tags=38%, list=24%, signal=49%
REACTOME_DNA_METHYLATION	23	0.6859534	1.624368	0	0.01611526	0.976	240	tags=26%, list=2%, signal=27%
WP_BRAINDERIVED_NEUROTROPHIC_FACTOR_BDNF_SIGNALING_PATHWAY	117	0.55133915	1.6222559	0	0.01652084	0.98	2149	tags=40%, list=17%, signal=48%
WP_TGFBETA_SIGNALING_PATHWAY	125	0.5379741	1.6192049	0	0.01727508	0.983	3578	tags=50%, list=27%, signal=68%
REACTOME_FCGR3A_MEDIATED_IL10_SYNTHESIS	34	0.6424358	1.6185812	0	0.01736201	0.983	2274	tags=41%, list=17%, signal=50%
REACTOME_CHROMATIN_MODIFYING_ENZYMES	222	0.52020615	1.6175526	0	0.01752481	0.984	3199	tags=45%, list=25%, signal=59%
REACTOME_RHO_GTPASE_CYCLE	409	0.5091111	1.6141326	0	0.01793562	0.988	2942	tags=38%, list=23%, signal=48%
REACTOME_SIGNALING_BY_BRAF_AND_RAF_FUSIONS	58	0.5965476	1.6071893	0	0.01881491	0.994	3395	tags=57%, list=26%, signal=77%
REACTOME_RHO_GTPASES_ACTIVATE_FORMINS	126	0.5394642	1.6073081	0	0.01883495	0.994	2121	tags=34%, list=16%, signal=40%

Supplemental Table 3: UP-REGULATED PATHWAYS: BRQ-GMP vs Veh-GMP

WP_ELECTRON_TRANSPORT_CHAIN_OXPHOS_SYSTEM_IN_MITOCHONDRIA	98	0.5545261	1.6077259	0	0.01888689	0.994	1491	tags=34%, list=11%, signal=38%
REACTOME_RHO_GTPASES_ACTIVATE_PAKS	18	0.70337296	1.6046921	0	0.01925847	0.995	683	tags=39%, list=5%, signal=41%
WP_RAS_SIGNALING	145	0.5313799	1.6042308	0	0.01929867	0.995	3160	tags=40%, list=24%, signal=52%
PID_RHOA_REG_PATHWAY	43	0.6218266	1.6026495	0	0.01939191	0.995	2420	tags=37%, list=19%, signal=46%
WP_SENESCENCE_AND_AUTOPHAGY_IN_CANCER	87	0.5531858	1.5976133	0	0.02019049	0.997	2696	tags=38%, list=21%, signal=48%
HALLMARK_COAGULATION	105	0.5446693	1.5986688	0	0.02021244	0.997	2464	tags=25%, list=19%, signal=30%
REACTOME_RHOA_GTPASE_CYCLE	136	0.5271041	1.5982049	0	0.02022412	0.997	2852	tags=40%, list=22%, signal=50%
PID_BETA_CATENIN_NUC_PATHWAY	56	0.5808633	1.5987004	0	0.02027359	0.997	3132	tags=52%, list=24%, signal=68%
WP_SPINAL_CORD_INJURY	83	0.5581856	1.5957199	0	0.02073561	0.997	2181	tags=29%, list=17%, signal=35%
PID_REG_GR_PATHWAY	63	0.5727669	1.5946584	0	0.02078289	0.997	2626	tags=48%, list=20%, signal=59%
REACTOME_G_PROTEIN_MEDIATED_EVENTS	46	0.601795	1.5948166	0	0.02080782	0.997	2262	tags=41%, list=17%, signal=50%
KEGG_OXIDATIVE_PHOSPHORYLATION	116	0.5421111	1.5889452	0	0.02238802	0.998	1491	tags=32%, list=11%, signal=36%
WP_ENDODERM_DIFFERENTIATION	102	0.5506205	1.5874027	0	0.0225687	0.998	2607	tags=40%, list=20%, signal=50%
PID_BCR_5PATHWAY	63	0.5674296	1.5842752	0	0.02370579	0.998	2345	tags=40%, list=18%, signal=48%
WP_RETINOBLASTOMA_GENE_IN_CANCER	86	0.54952115	1.5803679	0	0.02468649	0.999	4110	tags=62%, list=32%, signal=89%
REACTOME_ESR_MEDIATED_SIGNALING	161	0.5190732	1.5756907	0	0.02513302	1	2652	tags=39%, list=20%, signal=49%
KEGG_T_CELL_RECEPTOR_SIGNALING_PATHWAY	94	0.542233	1.5782766	0	0.02517653	1	2345	tags=33%, list=18%, signal=40%
WP_SIGNALING_PATHWAYS_IN_GLIOBLASTOMA	77	0.55662024	1.5770017	0	0.02522533	1	3396	tags=45%, list=26%, signal=61%
REACTOME_TRANSMISSION_ACROSS_CHEMICAL_SYNAPSES	170	0.5097441	1.5744501	0	0.02527981	1	2793	tags=30%, list=21%, signal=38%
HALLMARK_APOPTOSIS	141	0.5208793	1.5733632	0	0.02542233	1	2933	tags=37%, list=23%, signal=47%
HALLMARK_COMPLEMENT	167	0.516432	1.5682577	0	0.02690757	1	1401	tags=25%, list=11%, signal=27%
REACTOME_SUMOYLATION	159	0.5151872	1.5677955	0	0.02692337	1	3651	tags=50%, list=28%, signal=68%
WP_INTERFERON_TYPE_I_SIGNALING_PATHWAYS	53	0.5766841	1.5665909	0	0.02700892	1	2565	tags=49%, list=20%, signal=61%
HALLMARK_INTERFERON_ALPHA_RESPONSE	92	0.5437913	1.5634451	0	0.02764973	1	2719	tags=32%, list=21%, signal=40%
WP_NONALCOHOLIC_FATTY_LIVER_DISEASE	138	0.5251954	1.5582888	0	0.02940126	1	2191	tags=36%, list=17%, signal=43%
REACTOME_SIGNALING_BY_NUCLEAR_RECEPTORS	217	0.49767607	1.5487932	0	0.03193693	1	2894	tags=37%, list=22%, signal=47%
HALLMARK_OXIDATIVE_PHOSPHORYLATION	198	0.49257815	1.5271436	0	0.03791223	1	2865	tags=39%, list=22%, signal=49%
REACTOME_SIGNALING_BY_RECEPTOR_TYROSINE_KINASES	399	0.47598282	1.5213286	0	0.03932732	1	2598	tags=32%, list=20%, signal=39%
HALLMARK_APICAL_JUNCTION	152	0.49792892	1.5217936	0	0.03937436	1	2438	tags=30%, list=19%, signal=36%
WP_CALCIUM_REGULATION_IN_THE_CARDIAC_CELL	105	0.51911116	1.5215527	0	0.03938206	1	2262	tags=34%, list=17%, signal=41%
HALLMARK_INTERFERON_GAMMA_RESPONSE	179	0.49685407	1.5159123	0	0.04091343	1	2664	tags=30%, list=20%, signal=37%
REACTOME_SIGNALING_BY_GPCR	389	0.4733811	1.5060515	0	0.04478271	1	2534	tags=24%, list=19%, signal=29%
REACTOME_RHOG_GTPASE_CYCLE	72	0.5291789	1.4932646	0	0.04938989	1	2942	tags=46%, list=23%, signal=59%
REACTOME_TRANSCRIPTIONAL_REGULATION_BY_TP53	343	0.47204828	1.4910303	0	0.05042708	1	3262	tags=39%, list=25%, signal=51%
WP_IL18_SIGNALING_PATHWAY	216	0.46410456	1.44394	0	0.07072894	1	2909	tags=31%, list=22%, signal=40%
REACTOME_DISEASES_OF_SIGNAL_TRANSDUCTION_BY_GROWTH_FACTOR_RECEPTORS_AND_SECOND_MESSENGERS	345	0.44555038	1.4115448	0	0.08985397	1	3132	tags=37%, list=24%, signal=47%
NABA_MATRISOME_ASSOCIATED	365	0.42632747	1.3512862	0.00101215	0.13471055	1	2748	tags=19%, list=21%, signal=23%
REACTOME_NEURONAL_SYSTEM	229	0.48095396	1.5031931	0.00102459	0.04571464	1	2800	tags=28%, list=22%, signal=35%
WP_CILIARY_LANDSCAPE	203	0.46939296	1.4470253	0.00104058	0.0702666	1	2739	tags=35%, list=21%, signal=44%
HALLMARK_E2F_TARGETS	199	0.477345	1.4689553	0.00104275	0.06013922	1	2510	tags=36%, list=19%, signal=44%
KEGG_ALZHEIMERS_DISEASE	144	0.5041197	1.5207012	0.00106724	0.03948158	1	3076	tags=45%, list=24%, signal=58%
KEGG_ENDOCYTOSIS	154	0.49044546	1.4944216	0.00107066	0.0489805	1	2703	tags=33%, list=21%, signal=41%
REACTOME_G_ALPHA_Q_SIGNALLING_EVENTS	130	0.52455133	1.5756621	0.00108578	0.02505906	1	2722	tags=30%, list=21%, signal=38%
REACTOME_SIGNALING_BY_NTRKS	109	0.5206859	1.5249019	0.00108578	0.03853428	1	3143	tags=40%, list=24%, signal=53%
REACTOME_RESOLUTION_OF_SISTER_CHROMATID_COHESION	112	0.5106495	1.5168815	0.0010917	0.04072001	1	1895	tags=31%, list=15%, signal=36%
WP_TCELL_ANTIGEN_RECEPTOR_TCR_SIGNALING_PATHWAY	81	0.5589727	1.5980235	0.00110132	0.02020056	0.997	2345	tags=36%, list=18%, signal=43%
REACTOME_MITOTIC_SPINDLE_CHECKPOINT	107	0.50829554	1.4683906	0.00110497	0.06018681	1	4020	tags=51%, list=31%, signal=74%
KEGG_VASCULAR_SMOOTH_MUSCLE_CONTRACTION	80	0.5971244	1.686663	0.00111235	0.00843658	0.696	2269	tags=36%, list=17%, signal=44%
WP_RAC1PAK1P38MMP2_PATHWAY	64	0.6074259	1.6611241	0.00113636	0.01139159	0.855	2492	tags=41%, list=19%, signal=50%
WP_HIPPOMERLIN_SIGNALING_DYSREGULATION	86	0.56064034	1.5992429	0.00114025	0.02016705	0.997	2746	tags=36%, list=21%, signal=45%
REACTOME_PLATELET_HOMEOSTASIS	68	0.5625486	1.5780045	0.00114025	0.02522608	1	1734	tags=29%, list=13%, signal=34%
KEGG_B_CELL_RECEPTOR_SIGNALING_PATHWAY	72	0.5476197	1.556425	0.00114025	0.0297576	1	2345	tags=39%, list=18%, signal=47%
KEGG_ADHERENS_JUNCTION	66	0.5827847	1.6221923	0.0011534	0.01647413	0.98	1982	tags=39%, list=15%, signal=46%
WP_CORTICOTROPINRELEASING_HORMONE_SIGNALING_PATHWAY	76	0.5520107	1.5794097	0.00115607	0.02500421	1	2345	tags=37%, list=18%, signal=45%
KEGG_RENAL_CELL_CARCINOMA	64	0.5830464	1.6139746	0.00116144	0.01793874	0.988	3354	tags=53%, list=26%, signal=71%
WP_MELANOMA	60	0.5933165	1.6176645	0.00116414	0.01756658	0.984	2345	tags=40%, list=18%, signal=49%
REACTOME_TRANSCRIPTIONAL_REGULATION_BY_MECP2	45	0.596189	1.5653414	0.00116822	0.02738815	1	1970	tags=40%, list=15%, signal=47%
REACTOME_RNA_POLYMERASE_I_TRANSCRIPTION_INITIATION	46	0.6092613	1.6065637	0.00116959	0.01887957	0.994	3132	tags=48%, list=24%, signal=63%
REACTOME_FOXO_MEDIATED_TRANSCRIPTION	54	0.6076131	1.639638	0.00117096	0.01354999	0.939	3572	tags=65%, list=27%, signal=89%
REACTOME_ADORA2B_MEDIATED_ANTI_INFLAMMATORY_CYTOKINES_PRODUCTION	60	0.61682993	1.691335	0.00118064	0.00823621	0.658	1669	tags=28%, list=13%, signal=32%
WP_HEPATITIS_C_AND_HEPATOCELLULAR_CARCINOMA	43	0.63721997	1.6678194	0.00118064	0.01075781	0.82	2513	tags=42%, list=19%, signal=52%
REACTOME_PROTEIN_PROTEIN_INTERACTIONS_AT_SYNAPSES	48	0.5944664	1.5776583	0.00118203	0.02525172	1	2170	tags=29%, list=17%, signal=35%
REACTOME_INTERFERON_ALPHA_BETA_SIGNALING	48	0.6123551	1.6468142	0.00118765	0.01300211	0.923	2637	tags=35%, list=20%, signal=44%
WP_TGFBETA_RECEPTOR_SIGNALING	43	0.60872567	1.6054592	0.00120048	0.01909442	0.995	1980	tags=37%, list=15%, signal=44%
PID_RHOA_PATHWAY	41	0.66782886	1.7443794	0.00120627	0.00486199	0.312	2232	tags=49%, list=17%, signal=59%
REACTOME_G_ALPHA_Z_SIGNALLING_EVENTS	33	0.6554725	1.6548351	0.00120627	0.01219263	0.879	1669	tags=36%, list=13%, signal=42%
PID_A6B1_A6B4_INTEGRIN_PATHWAY	36	0.65036607	1.6736764	0.00120773	0.0101482	0.779	1229	tags=36%, list=9%, signal=40%
WP_AMYOTROPHIC_LATERAL_SCLEROSIS_ALS	34	0.6805858	1.6903147	0.00121507	0.0081877	0.667	882	tags=26%, list=7%, signal=28%
SIG_PIP3_SIGNALING_IN_B_LYMPHOCYTES	34	0.6515694	1.6692826	0.001221	0.01068302	0.81	2345	tags=53%, list=18%, signal=64%
BIOCARTA_FMLP_PATHWAY	31	0.6893929	1.7304021	0.00122399	0.00551952	0.385	2290	tags=52%, list=18%, signal=62%
PID_ER_NONGENOMIC_PATHWAY	36	0.6615682	1.6944383	0.00122399	0.00809839	0.633	2806	tags=56%, list=22%, signal=71%
WP_PHOTODYNAMIC_THERAPYINDUCED_HIF1_SURVIVAL_SIGNALING	30	0.65007514	1.6112915	0.00122549	0.01817971	0.991	1889	tags=27%, list=15%, signal=31%
PID_NECTIN_PATHWAY	30	0.70627904	1.7570859	0.00123001	0.00472966	0.255	2372	tags=53%, list=18%, signal=65%
PID_EPHB_FWD_PATHWAY	34	0.6673328	1.6926315	0.00123001	0.0083176	0.65	2716	tags=62%, list=21%, signal=78%
WP_GLYCOGEN_SYNTHESIS_AND_DEGRADATION	35	0.66512066	1.6741486	0.00123001	0.01016471	0.776	1776	tags=43%, list=14%, signal=49%
WP_REGUCALCIN_IN_PROXIMAL_TUBULE_EPITHELIAL_KIDNEY_CELLS	26	0.69790304	1.7035105	0.00123457	0.0073627	0.566	2345	tags=54%, list=18%, signal=66%
REACTOME_REGULATION_OF_TP53_ACTIVITY_THROUGH_ACETYLATION	30	0.64203614	1.6155367	0.00123762	0.01776798	0.985	3262	tags=63%, list=25%, signal=84%
BIOCARTA_IL2RB_PATHWAY	34	0.67814904	1.7392226	0.00124069	0.00505239	0.332	2608	tags=62%, list=20%, signal=77%
WP_PRADERWILLI_AND_ANGELMAN_SYNDROME	31	0.6648938	1.6532788	0.00124069	0.01236082	0.889	1630	tags=35%, list=13%, signal=40%
REACTOME_ACTIVATION_OF_KAINATE_RECEPTORS_UPON_GLUTAMATE_BINDING	22	0.71923786	1.6965318	0.00125156	0.00787229	0.621	1669	tags=41%, list=13%, signal=47%
PID_NCADHERIN_PATHWAY	29	0.6897645	1.7130036	0.00125313	0.00671021	0.496	2519	tags=48%, list=19%, signal=60%
REACTOME_THE_ROLE_OF_NEF_IN_HIV_1_REPLICATION_AND_DISEASE_PATHOGENESIS	28	0.65340406	1.6031989	0.00125313	0.01941982	0.995	3449	tags=61%, list=27%, signal=82%
SIG_REGULATION_OF_THE_ACTIN_CYTOSKELETON_BY_RHO_GTPASES	25	0.709929	1.7269905	0.00125786	0.00568058	0.404	2535	tags=64%, list=19%, signal=79%
SA_B_CELL_RECEPTOR_COMPLEXES	23	0.67506146	1.613634	0.00126422	0.01793773	0.988	2057	tags=52%, list=16%, signal=62%
WP_GASTRIC_CANCER_NETWORK_1	23	0.6886254	1.6419507	0.00126582	0.01336555	0.934	1676	tags=52%, list=13%, signal=60%
BIOCARTA_RAC1_PATHWAY	21	0.7328452	1.7212669	0.00126904	0.00591722	0.439	2162	tags=62%, list=17%, signal=74%
REACTOME_RHO_GTPASES_ACTIVATE_IQGAPS	22	0.7286523	1.7240366	0.00127226	0.00574777	0.425	1571	tags=50%, list=12%, signal=57%

PID_IL2_STAT5_PATHWAY	27	0.6822872	1.6711143	0.00127714	0.01043929	0.797	2492	tags=52%, list=19%, signal=64%
WP_OXIDATIVE_STRESS	27	0.67667705	1.6564562	0.00127714	0.01197895	0.872	2178	tags=41%, list=17%, signal=49%
PID_GMCSF_PATHWAY	33	0.6552873	1.642283	0.00127714	0.01341816	0.932	3105	tags=61%, list=24%, signal=79%
BIOCARTA_CALCINEURIN_PATHWAY	17	0.7370992	1.6378953	0.00129199	0.01383463	0.946	923	tags=47%, list=7%, signal=51%
WP_CARDIAC_PROGENITOR_DIFFERENTIATION	24	0.70853156	1.7030449	0.00129366	0.00733677	0.568	2149	tags=42%, list=17%, signal=50%
REACTOME_GLUCAGON_SIGNALING_IN_METABOLIC_REGULATION	25	0.739445	1.7739719	0.00130039	0.00382685	0.177	2262	tags=52%, list=17%, signal=63%
WP_GLUTATHIONE_METABOLISM	18	0.7400074	1.6898981	0.00130548	0.0081959	0.673	1552	tags=33%, list=12%, signal=38%
REACTOME_RHO_GTPASES_ACTIVATE_ROCKS	16	0.75319374	1.6607877	0.00130548	0.01139183	0.856	2019	tags=63%, list=16%, signal=74%
REACTOME_OTHER_SEMAPHORIN_INTERACTIONS	16	0.77625513	1.7127206	0.00131234	0.00661811	0.497	467	tags=25%, list=4%, signal=26%
BIOCARTA_CTCF_PATHWAY	21	0.69765276	1.6365126	0.00131406	0.01401081	0.948	2492	tags=57%, list=19%, signal=71%
REACTOME_SIGNALING_BY_CYTOSOLIC_FGFR1_FUSION_MUTANTS	18	0.732622	1.6732876	0.0013369	0.01009355	0.784	2565	tags=72%, list=20%, signal=90%
KEGG_PATHWAYS_IN_CANCER	251	0.4593264	1.4328706	0.00205128	0.07640938	1	2565	tags=32%, list=20%, signal=39%
HALLMARK_HEME_METABOLISM	184	0.49327692	1.520293	0.00206825	0.03957978	1	2452	tags=30%, list=19%, signal=37%
REACTOME_MITOTIC_PROMETAPHASE	187	0.47824785	1.4724727	0.00209424	0.05895212	1	3246	tags=39%, list=25%, signal=51%
REACTOME_CELLULAR_SENESCENCE	145	0.4736847	1.4184577	0.00214592	0.08603547	1	2050	tags=30%, list=16%, signal=35%
WP_ECTODERM_DIFFERENTIATION	97	0.5121305	1.4965365	0.00220264	0.04815481	1	2921	tags=32%, list=22%, signal=41%
REACTOME_L1CAM_INTERACTIONS	87	0.537598	1.5463182	0.00222222	0.03265702	1	2598	tags=34%, list=20%, signal=43%
PID_AVB3_INTEGRIN_PATHWAY	55	0.5844713	1.5885701	0.00225734	0.02235974	0.998	2458	tags=40%, list=19%, signal=49%
WP_FRAGILE_X_SYNDROME	93	0.52468574	1.5058496	0.00226501	0.04468329	1	3139	tags=42%, list=24%, signal=55%
WP_LEPTIN_SIGNALING_PATHWAY	74	0.5602392	1.5638336	0.00226757	0.02765314	1	1954	tags=38%, list=15%, signal=44%
REACTOME_TRANSLOCATION_OF_SLC2A4_GLUT4_TO_THE_PLASMA_MEMBRANE	61	0.6101601	1.6761689	0.00227531	0.0099931	0.767	1949	tags=36%, list=15%, signal=42%
REACTOME_RAC3_GTPASE_CYCLE	83	0.57042634	1.6150507	0.0022779	0.01779693	0.985	2134	tags=40%, list=16%, signal=47%
KEGG_PANCREATIC_CANCER	64	0.5757097	1.5669216	0.00229358	0.02710563	1	3105	tags=45%, list=24%, signal=59%
WP_PROLACTIN_SIGNALING_PATHWAY	73	0.5692042	1.581204	0.00230681	0.02452848	0.999	2565	tags=47%, list=20%, signal=58%
WP_HUMAN_THYROID_STIMULATING_HORMONE_TSH_SIGNALING_PATHWAY	61	0.59423953	1.640414	0.00230947	0.0135408	0.937	2565	tags=54%, list=20%, signal=67%
REACTOME_RNA_POLYMERASE_II_TRANSCRIPTION_TERMINATION	65	0.56915253	1.5700268	0.00230947	0.02647831	1	3248	tags=54%, list=25%, signal=71%
PID_VEGFR1_2_PATHWAY	68	0.5772078	1.5996737	0.00231481	0.02011224	0.996	2473	tags=51%, list=19%, signal=63%
WP_REGULATORY_CIRCUITS_OF_THE_STAT3_SIGNALING_PATHWAY	67	0.56130916	1.5769583	0.00232829	0.02516571	1	3712	tags=42%, list=29%, signal=58%
PID_MYC_REPRESS_PATHWAY	57	0.6060921	1.6475712	0.00235294	0.01296366	0.918	2933	tags=47%, list=23%, signal=61%
PID_TXA2PATHWAY	53	0.59458363	1.6129754	0.00235294	0.01805033	0.988	2722	tags=43%, list=21%, signal=55%
PID_TGFBR_PATHWAY	49	0.6009567	1.6163025	0.00235849	0.01759378	0.985	1933	tags=37%, list=15%, signal=43%
WP_PATHOGENIC_ESCHERICHIA_COLI_INFECTION	45	0.6219501	1.6519874	0.00236407	0.01250874	0.894	1816	tags=44%, list=14%, signal=51%
REACTOME_SENSORY_PROCESSING_OF_SOUND_BY_OUTER_HAIR_CELLS_OF_THE_COCHLEA	37	0.63681304	1.6398152	0.00236967	0.01356447	0.939	1681	tags=32%, list=13%, signal=37%
PID_LYSOPHOSPHOLIPID_PATHWAY	53	0.59889686	1.629574	0.00237812	0.0151196	0.967	2699	tags=43%, list=21%, signal=55%
PID_FCER1_PATHWAY	57	0.5711525	1.5442665	0.00238095	0.03325477	1	2555	tags=42%, list=20%, signal=52%
KEGG_PATHOGENIC_ESCHERICHIA_COLI_INFECTION	45	0.62195003	1.6315358	0.00239808	0.01479481	0.961	1816	tags=44%, list=14%, signal=51%
PID_IFNG_PATHWAY	39	0.6254085	1.5918837	0.00239808	0.02143116	0.998	2934	tags=62%, list=23%, signal=79%
PID_HEDGEHOG_GLI_PATHWAY	39	0.6223438	1.5774509	0.00240096	0.02525438	1	3369	tags=64%, list=26%, signal=86%
PID_PLK1_PATHWAY	45	0.6062151	1.5880746	0.00240674	0.02238066	0.998	2050	tags=42%, list=16%, signal=50%
PID_AJDISS_2PATHWAY	38	0.6367818	1.6516824	0.00242131	0.01254097	0.897	1277	tags=34%, list=10%, signal=38%
BIOCARTA_FCER1_PATHWAY	38	0.62068427	1.5955269	0.00242424	0.02073615	0.997	2290	tags=50%, list=18%, signal=61%
KEGG_AMYOTROPHIC_LATERAL_SCLEROSIS_ALS	42	0.6230702	1.629016	0.002442	0.01520508	0.967	2290	tags=38%, list=18%, signal=46%
KEGG_ARACHIDONIC_ACID_METABOLISM	32	0.6466087	1.6119989	0.002442	0.01813952	0.99	1025	tags=22%, list=8%, signal=24%
PID_ENDOTHELIN_PATHWAY	51	0.5970333	1.6073124	0.00246305	0.01890602	0.994	2810	tags=43%, list=22%, signal=55%
PID_LKB1_PATHWAY	44	0.6330235	1.6427177	0.00246609	0.01340286	0.931	2492	tags=48%, list=19%, signal=59%
PID_CDC42_REG_PATHWAY	29	0.6510238	1.6167347	0.00248447	0.0176267	0.985	1946	tags=48%, list=15%, signal=57%
KEGG_STARCH_AND_SUCROSE_METABOLISM	25	0.6801851	1.6447185	0.0025	0.01321547	0.928	2210	tags=40%, list=17%, signal=48%
PID_EPO_PATHWAY	32	0.64856696	1.6335387	0.00250941	0.01442148	0.959	2939	tags=56%, list=23%, signal=72%
BIOCARTA_RACCYCD_PATHWAY	26	0.7014242	1.7009146	0.00252845	0.00753598	0.584	2345	tags=54%, list=18%, signal=66%
PID_IGF1_PATHWAY	26	0.66354626	1.5773636	0.00253165	0.02521381	1	2458	tags=54%, list=19%, signal=66%
REACTOME_ANTIGEN_ACTIVATES_B_CELL_RECEPTOR_BCR_LEADING_TO_GENERATION_OF_SECOND_MESSENGERS	32	0.638386	1.5791934	0.00254453	0.02499412	1	2069	tags=47%, list=16%, signal=56%
BIOCARTA_PAR1_PATHWAY	18	0.76786697	1.7452229	0.00254777	0.00486668	0.305	1989	tags=67%, list=15%, signal=79%
REACTOME_PROSTACYCLIN_SIGNALLING_THROUGH_PROSTACYCLIN_RECEPTOR	16	0.79052097	1.7317886	0.00256082	0.00566134	0.379	1669	tags=50%, list=13%, signal=57%
WP_REGULATION_OF_APOPTOSIS_BY_PARATHYROID_HORMONERELATED_PROTEIN	20	0.71180266	1.6760505	0.00256082	0.00995554	0.768	2492	tags=45%, list=19%, signal=56%
PID_HDAC_CLASSIII_PATHWAY	23	0.68821114	1.5985107	0.0025641	0.02019601	0.997	2652	tags=61%, list=20%, signal=76%
REACTOME_G_PROTEIN_ACTIVATION	20	0.74098957	1.7204075	0.00256739	0.00593992	0.445	889	tags=35%, list=7%, signal=38%
BIOCARTA_MAL_PATHWAY	16	0.7818637	1.7445697	0.00257732	0.00489696	0.31	773	tags=50%, list=6%, signal=53%
BIOCARTA_NOS1_PATHWAY	16	0.759075	1.6892285	0.00258065	0.00822545	0.678	1554	tags=56%, list=12%, signal=64%
WP_INTERACTOME_OF_POLYCOMB_REPRESSIVE_COMPLEX_2_PRC2	16	0.7307569	1.5976429	0.00258732	0.0202504	0.997	2926	tags=75%, list=22%, signal=97%
BIOCARTA_SPPA_PATHWAY	16	0.74605525	1.6667017	0.00263158	0.01083082	0.83	1954	tags=63%, list=15%, signal=73%
PID_S1P_S1P2_PATHWAY	21	0.7134511	1.6826218	0.00265252	0.00895022	0.716	1302	tags=43%, list=10%, signal=48%
WP_PHYSIOLOGICAL_AND_PATHOLOGICAL_HYPERTROPHY_OF_THE_HEART	21	0.71751994	1.6913068	0.00265604	0.0081852	0.658	2534	tags=62%, list=19%, signal=77%
PID_EPHA2_FWD_PATHWAY	18	0.72657686	1.6412411	0.00266312	0.01341963	0.934	716	tags=44%, list=6%, signal=47%
HALLMARK_IL2_STAT5_SIGNALING	179	0.46606788	1.4188641	0.0031679	0.08580639	1	2083	tags=22%, list=16%, signal=26%
WP_HEPATITIS_B_INFECTION	128	0.50271285	1.5076833	0.00322234	0.04449519	1	2810	tags=38%, list=22%, signal=47%
KEGG_JAK_STAT_SIGNALING_PATHWAY	101	0.5185926	1.5281179	0.00326442	0.03831075	1	2810	tags=34%, list=22%, signal=43%
NABA_ECM_AFFILIATED	86	0.54712576	1.5636847	0.00334448	0.02764249	1	1167	tags=16%, list=9%, signal=18%
REACTOME_RNA_POLYMERASE_I_TRANSCRIPTION	68	0.5478936	1.5452251	0.00337458	0.03305946	1	2989	tags=43%, list=23%, signal=55%
REACTOME_PI3K_AKT_SIGNALING_IN_CANCER	79	0.5310209	1.5175976	0.00343249	0.04054083	1	3449	tags=39%, list=27%, signal=53%
WP_CARDIAC_HYPERTROPHIC_RESPONSE	50	0.5967962	1.5948441	0.00352941	0.02087265	0.997	4120	tags=64%, list=32%, signal=93%
PID_FAK_PATHWAY	56	0.5995787	1.6342913	0.00353357	0.01435631	0.955	2569	tags=46%, list=20%, signal=58%
REACTOME_B_WICH_COMPLEX_POSITIVELY_REGULATES_RRNA_EXPRESSION	49	0.5801307	1.5410316	0.00353357	0.034141	1	2652	tags=41%, list=20%, signal=51%
BIOCARTA_PPARA_PATHWAY	47	0.61415434	1.6497542	0.00353774	0.01270339	0.902	2492	tags=47%, list=19%, signal=58%
PID_AR_NONGENOMIC_PATHWAY	27	0.6898886	1.6638665	0.00359281	0.01106079	0.845	1954	tags=48%, list=15%, signal=57%
PID_IL12_2PATHWAY	51	0.57621723	1.5598378	0.00359281	0.02892217	1	2954	tags=39%, list=23%, signal=51%
BIOCARTA_PYK2_PATHWAY	26	0.6681143	1.6477797	0.00366748	0.01304688	0.917	2232	tags=58%, list=17%, signal=69%
REACTOME_INTERLEUKIN_2_FAMILY_SIGNALING	37	0.62001216	1.6080371	0.00369458	0.01888351	0.994	2810	tags=54%, list=22%, signal=69%
REACTOME_DAP12_INTERACTIONS	33	0.65213007	1.6505308	0.00376412	0.01274987	0.9	703	tags=24%, list=5%, signal=26%
BIOCARTA_HER2_PATHWAY	19	0.70863795	1.6122357	0.00376412	0.01816557	0.99	1954	tags=58%, list=15%, signal=68%
WP_TUMOR_SUPPRESSOR_ACTIVITY_OF_SMARCB1	25	0.68472767	1.6258328	0.00378788	0.01585548	0.973	1154	tags=36%, list=9%, signal=39%
PID_NETRIN_PATHWAY	27	0.6566741	1.6147227	0.00380228	0.01782147	0.987	2131	tags=48%, list=16%, signal=57%
BIOCARTA_GPCR_PATHWAY	27	0.66549754	1.6330816	0.00380711	0.01449411	0.96	893	tags=37%, list=7%, signal=40%
BIOCARTA_AGR_PATHWAY	23	0.6956489	1.6396092	0.00381679	0.01349317	0.939	2526	tags=57%, list=19%, signal=70%
WP_MIRNAS_INVOLVED_IN_DNA_DAMAGE_RESPONSE	15	0.73516726	1.6267864	0.00389105	0.01566774	0.972	1124	tags=40%, list=9%, signal=44%
WP_ESTROGEN_SIGNALING_PATHWAY	22	0.688756	1.6170988	0.00391134	0.01759313	0.984	2345	tags=55%, list=18%, signal=66%

REACTOME_RHO_GTPASES_ACTIVATE_CIT	16	0.73785144	1.6476556	0.00393185	0.01299726	0.917	2019	tags=56%, list=16%, signal=67%
BIOCARTA_GCR_PATHWAY	16	0.75432867	1.6962583	0.00394737	0.00783641	0.621	893	tags=50%, list=7%, signal=54%
REACTOME_CELL_CYCLE_MITOTIC	490	0.40796924	1.3023995	0.00401204	0.17811206	1	3262	tags=36%, list=25%, signal=46%
REACTOME_INTRACELLULAR_SIGNALING_BY_SECOND_MESSENGERS	268	0.43597636	1.3733459	0.0040568	0.11761728	1	2361	tags=31%, list=18%, signal=38%
REACTOME_SIGNALING_BY_WNT	244	0.43779996	1.3712202	0.00409417	0.11852387	1	2722	tags=33%, list=21%, signal=41%
REACTOME_INTERFERON_SIGNALING	153	0.46402022	1.4006256	0.00419727	0.09779321	1	2810	tags=37%, list=22%, signal=46%
REACTOME_TRANSCRIPTIONAL_REGULATION_BY_RUNX1	181	0.4758024	1.4583509	0.00422387	0.06427941	1	1950	tags=29%, list=15%, signal=33%
WP_DNA_DAMAGE_RESPONSE_ONLY_ATM_DEPENDENT	94	0.52876717	1.5268637	0.00437637	0.03792599	1	2554	tags=40%, list=20%, signal=50%
REACTOME_TRANSPORT_OF_MATURE_TRANSCRIPT_TO_CYTOPLASM	81	0.5279086	1.4972993	0.00446429	0.04796569	1	3248	tags=44%, list=25%, signal=59%
WP_VIRAL_ACUTE_MYOCARDITIS	75	0.54596263	1.532582	0.0045045	0.03682989	1	2645	tags=37%, list=20%, signal=47%
PID_SMAD2_3NUCLEAR_PATHWAY	70	0.5489515	1.5224476	0.0045403	0.03914041	1	2492	tags=41%, list=19%, signal=51%
WP_APOPTOSIS	73	0.54497844	1.5295713	0.00457143	0.03782171	1	2816	tags=33%, list=22%, signal=42%
WP_NONSMALL_CELL_LUNG_CANCER	66	0.55409265	1.5633453	0.0045819	0.02761588	1	3262	tags=48%, list=25%, signal=64%
WP_KIT_RECEPTOR_SIGNALING_PATHWAY	56	0.56616646	1.5426075	0.00461361	0.03351763	1	2083	tags=45%, list=16%, signal=53%
KEGG_PHOSPHATIDYLINOSITOL_SIGNALING_SYSTEM	69	0.5450625	1.5256352	0.00464576	0.03829693	1	2421	tags=33%, list=19%, signal=41%
PID_IL4_2PATHWAY	52	0.5835629	1.5696653	0.00466744	0.02653581	1	2406	tags=33%, list=18%, signal=40%
PID_TRKR_PATHWAY	51	0.6065513	1.6343403	0.00473934	0.01441502	0.955	3154	tags=53%, list=24%, signal=70%
PID_ECADHERIN_STABILIZATION_PATHWAY	38	0.61341876	1.576847	0.00484849	0.02505432	1	2362	tags=42%, list=18%, signal=51%
REACTOME_RUNX1_INTERACTS_WITH_CO_FACTORS_WHOSE_PRECISE_EFFECT_ON_RUNX1_TARGETS_IS_NOT_KNOWN	35	0.6092838	1.557044	0.00494438	0.02966319	1	1950	tags=43%, list=15%, signal=50%
WP_TYPE_II_INTERFERON_SIGNALING_IFNG	28	0.65270454	1.5840472	0.00501253	0.02371325	0.998	2128	tags=43%, list=16%, signal=51%
KEGG_GRAFT_VERSUS_HOST_DISEASE	20	0.72797424	1.7027284	0.00502513	0.00730289	0.569	975	tags=15%, list=7%, signal=16%
BIOCARTA_CELLCYCLE_PATHWAY	21	0.71473235	1.673354	0.00505689	0.01013041	0.782	2036	tags=48%, list=16%, signal=56%
KEGG_ALLOGRAFT_REJECTION	20	0.6991887	1.6164916	0.00506971	0.01763104	0.985	975	tags=15%, list=7%, signal=16%
REACTOME_INWARDLY_RECTIFYING_K_CHANNELS	21	0.7013553	1.6453052	0.00516129	0.0131735	0.927	1669	tags=33%, list=13%, signal=38%
BIOCARTA_GSK3_PATHWAY	18	0.7090134	1.6087594	0.00525624	0.01873313	0.994	2519	tags=56%, list=19%, signal=69%
HALLMARK_KRAS_SIGNALING_UP	149	0.49001312	1.4714686	0.00533618	0.05940607	1	3500	tags=30%, list=27%, signal=40%
KEGG_UBIQUITIN_MEDIATED_PROTEOLYSIS	128	0.47246107	1.4130931	0.00539374	0.08875426	1	3912	tags=53%, list=30%, signal=75%
HALLMARK_UV_RESPONSE_DN	122	0.49712253	1.4686613	0.00539957	0.06013603	1	2534	tags=32%, list=19%, signal=39%
PID_P53_DOWNSTREAM_PATHWAY	116	0.4829851	1.4426439	0.00541126	0.07144105	1	3188	tags=32%, list=25%, signal=42%
KEGG_AXON_GUIDANCE	94	0.5115788	1.5028434	0.00545256	0.04559545	1	3449	tags=37%, list=27%, signal=50%
REACTOME_CELL_SURFACE_INTERACTIONS_AT_THE_VASCULAR_WALL	106	0.49841884	1.4666992	0.00547645	0.06093353	1	2763	tags=31%, list=21%, signal=39%
KEGG_WNT_SIGNALING_PATHWAY	112	0.51150304	1.5030029	0.00551876	0.04561737	1	2797	tags=38%, list=21%, signal=47%
REACTOME_ESTROGEN_DEPENDENT_GENE_EXPRESSION	103	0.5057624	1.4821557	0.00553097	0.05377061	1	2652	tags=41%, list=20%, signal=51%
REACTOME_MHC_CLASS_II_ANTIGEN_PRESENTATION	102	0.500281	1.4673405	0.00553097	0.06065967	1	3357	tags=43%, list=26%, signal=58%
REACTOME_RHOB_GTPASE_CYCLE	67	0.541628	1.5015776	0.0056243	0.0459525	1	2019	tags=36%, list=16%, signal=42%
WP_PATHWAYS_REGULATING_HIPPO_SIGNALING	71	0.5337825	1.4980023	0.00566251	0.04766111	1	2722	tags=34%, list=21%, signal=43%
WP_ANDROGEN_RECEPTOR_SIGNALING_PATHWAY	84	0.54046655	1.5491838	0.00567537	0.03187208	1	2652	tags=48%, list=20%, signal=59%
REACTOME_MRNA_SPLICING_MINOR_PATHWAY	52	0.5758787	1.5577598	0.00580046	0.02945738	1	2766	tags=50%, list=21%, signal=63%
BIOCARTA_HIVNEF_PATHWAY	54	0.572555	1.5743726	0.00584795	0.02524418	1	2816	tags=41%, list=22%, signal=52%
REACTOME_NRAGE_SIGNALS_DEATH_THROUGH_JNK	53	0.5569651	1.5121459	0.00587544	0.0426703	1	1415	tags=26%, list=11%, signal=30%
WP_TGFBETA_RECEPTOR_SIGNALLING_IN_SKELETAL_DYSPLASIAS	47	0.5897832	1.5582548	0.00592417	0.02933411	1	1980	tags=34%, list=15%, signal=40%
PID_PS1_PATHWAY	37	0.60400224	1.5273314	0.00596659	0.03803235	1	2519	tags=49%, list=19%, signal=60%
REACTOME_COPI_INDEPENDENT_GOLGI_TO_ER_RETROGRADE_TRAFFIC	41	0.606602	1.5737461	0.0060024	0.02537809	1	2784	tags=49%, list=21%, signal=62%
BIOCARTA_BCR_PATHWAY	32	0.61934686	1.5552286	0.0060241	0.0301235	1	2290	tags=47%, list=18%, signal=57%
WP_FAS_LIGAND_FASL_PATHWAY_AND_STRESS_INDUCTION_OF_HEAT_SHOCK_PROTEINS_HSP_REGULATION	38	0.6016356	1.5441875	0.00603136	0.03318705	1	2608	tags=45%, list=20%, signal=56%
REACTOME_RECYCLING_PATHWAY_OF_L1	37	0.6197587	1.5884975	0.00606796	0.02230946	0.998	2598	tags=41%, list=20%, signal=51%
REACTOME_SIGNALING_BY_MODERATE_KINASE_ACTIVITY_BRAF_MUTANTS	40	0.61437684	1.594631	0.00611247	0.0206446	0.997	3395	tags=60%, list=26%, signal=81%
REACTOME_SIGNALING_BY_FGFR_IN_DISEASE	49	0.5913546	1.576856	0.00614251	0.02512609	1	3808	tags=57%, list=29%, signal=80%
BIOCARTA_EIF4_PATHWAY	23	0.67703617	1.5928078	0.00617284	0.02117552	0.998	2345	tags=61%, list=18%, signal=74%
BIOCARTA_AT1R_PATHWAY	25	0.66699296	1.5703114	0.00619579	0.02647079	1	2057	tags=52%, list=16%, signal=62%
PID_CXCR3_PATHWAY	38	0.6185802	1.6028423	0.00622665	0.01942477	0.995	2716	tags=45%, list=21%, signal=56%
REACTOME_TRIGLYCERIDE_CATABOLISM	16	0.72229004	1.6071355	0.0062422	0.01876405	0.994	1316	tags=38%, list=10%, signal=42%
WP_THE_EFFECT_OF_PROGERIN_ON_THE_INVOLVED_GENES_IN_HUTCHINSONGILFORD_PROGERIA_SYNDROME	27	0.67733735	1.6738046	0.00625782	0.01019238	0.779	1899	tags=52%, list=15%, signal=61%
WP_MITOCHONDRIAL_CIV_ASSEMBLY	33	0.6265402	1.5768138	0.00633714	0.02498294	1	1575	tags=42%, list=12%, signal=48%
REACTOME_FGFR1_MUTANT_RECEPTOR_ACTIVATION	23	0.6793035	1.6017678	0.00639386	0.0195543	0.996	2565	tags=57%, list=20%, signal=70%
WP_MESODERMAL_COMMITMENT_PATHWAY	105	0.49323586	1.4357802	0.00650759	0.07517006	1	2612	tags=32%, list=20%, signal=40%
KEGG_TASTE_TRANSDUCTION	18	0.71437305	1.6242257	0.00652742	0.01609148	0.976	462	tags=22%, list=4%, signal=23%
WP_REGULATION_OF_TOLLLIKE_RECEPTOR_SIGNALING_PATHWAY	110	0.49761283	1.4505758	0.00654308	0.06855543	1	2345	tags=29%, list=18%, signal=35%
WP_MBDNF_AND_PROBDNF_REGULATION_OF_GABA_NEUROTRANSMISSION	19	0.69927776	1.5790431	0.0065445	0.02496375	1	876	tags=42%, list=7%, signal=45%
PID_THROMBIN_PAR4_PATHWAY	15	0.7362841	1.6394688	0.00665779	0.01344605	0.939	634	tags=40%, list=5%, signal=42%
WP_REGULATION_OF_SISTER_CHROMATID_SEPARATION_AT_THE_METAPHASEANAPHASE_TRANSITION	15	0.7206461	1.6113985	0.00668449	0.01823761	0.991	2439	tags=60%, list=19%, signal=74%
WP_CHROMOSOMAL_AND_MICROSATELLITE_INSTABILITY_IN_COLORECTAL_CANCER	68	0.53600794	1.4847918	0.00672646	0.05274444	1	2669	tags=43%, list=21%, signal=53%
REACTOME_G2_M_DNA_DAMAGE_CHECKPOINT	68	0.54317206	1.5209043	0.00696056	0.03945767	1	1195	tags=21%, list=9%, signal=23%
PID_E2F_PATHWAY	65	0.5325135	1.4895484	0.00699301	0.05094867	1	2652	tags=43%, list=20%, signal=54%
SIG_PIP3_SIGNALING_IN_CARDIAC_MYOCTES	59	0.5519014	1.5065334	0.00700117	0.04471295	1	2939	tags=47%, list=23%, signal=61%
KEGG_SMALL_CELL_LUNG_CANCER	71	0.5346648	1.4990003	0.00702576	0.04726487	1	3707	tags=44%, list=28%, signal=61%
REACTOME_ACTIVATION_OF_ANTERIOR_HOX_GENES_IN_HINDBRAIN_DEVELOPMENT_DURING_EARLY_EMBRYOGENESIS	75	0.5290518	1.5087829	0.007034	0.04405196	1	2612	tags=33%, list=20%, signal=41%
PID_AR_PATHWAY	53	0.5529029	1.5016066	0.00707547	0.04602328	1	2561	tags=47%, list=20%, signal=58%
REACTOME_MAPK_FAMILY_SIGNALING_CASCADES	267	0.42596734	1.3355165	0.00714286	0.14690699	1	2279	tags=27%, list=18%, signal=32%
WP_IL6_SIGNALING_PATHWAY	39	0.5875334	1.5167719	0.00722892	0.04066566	1	2939	tags=49%, list=23%, signal=63%
WP_COPPER_HOMEOSTASIS	41	0.5836154	1.5114107	0.00724638	0.04278951	1	2509	tags=51%, list=19%, signal=63%
WP_GLYCOLYSIS_AND_GLUCONEOGENESIS	37	0.6073585	1.5651331	0.00728155	0.02730316	1	1889	tags=32%, list=15%, signal=38%
BIOCARTA_TCR_PATHWAY	41	0.59562165	1.5434507	0.0072904	0.03329821	1	2290	tags=41%, list=18%, signal=50%
WP_KISSPEPTINKISSPEPTIN_RECEPTOR_SYSTEM_IN_THE_OVARY	35	0.60403067	1.5146637	0.00732601	0.04154675	1	3262	tags=49%, list=25%, signal=65%
PID_S1P_S1P3_PATHWAY	27	0.6498518	1.5814835	0.00741656	0.02445574	0.999	2810	tags=56%, list=22%, signal=71%
WP_CANNABINOID_RECEPTOR_SIGNALING	21	0.6690209	1.5737364	0.00759494	0.02530792	1	2262	tags=48%, list=17%, signal=58%
BIOCARTA_HDAC_PATHWAY	22	0.67150927	1.5666136	0.00765306	0.02706904	1	2345	tags=55%, list=18%, signal=66%
WP_TCELL_RECEPTOR_AND_COSTIMULATORY_SIGNALING	26	0.6218941	1.529787	0.00767263	0.03781766	1	876	tags=27%, list=7%, signal=29%
REACTOME_NEF_MEDIATES_DOWN_MODULATION_OF_CELL_SURFACE_RECEPTORS_BY_RECRUITING_THEM_TO_CLATHRIN_ADAPTERS	21	0.6570008	1.5114791	0.00768246	0.04285688	1	3203	tags=57%, list=25%, signal=76%
BIOCARTA_EDG1_PATHWAY	19	0.7090896	1.5946321	0.00776197	0.0207153	0.997	2372	tags=63%, list=18%, signal=77%
REACTOME_CD209_DC_SIGN_SIGNALING	19	0.6786115	1.5691527	0.00777202	0.02662678	1	3788	tags=79%, list=29%, signal=111%
REACTOME_NEGATIVE_REGULATION_OF_THE_PI3K_AKT_NETWORK	87	0.5167615	1.4710858	0.0078125	0.05936008	1	3449	tags=39%, list=27%, signal=53%
WP_TNF_ALPHA_SIGNALING_PATHWAY	90	0.50903904	1.4812949	0.00782123	0.05396841	1	3712	tags=44%, list=29%, signal=62%

REACTOME_BETA_CATENIN_PHOSPHORYLATION_CASCADE	17	0.7132913	1.6041944	0.0078637	0.01923951	0.995	1226	tags=41%, list=9%, signal=45%
REACTOME_ACTIVATION_OF_NMDA_RECEPTORS_AND_POSTSYNAPTIC_EVENTS	66	0.5449925	1.5271616	0.00807382	0.03800336	1	3044	tags=41%, list=23%, signal=53%
KEGG_MELANOMA	53	0.55245733	1.4937282	0.00811124	0.04934496	1	3683	tags=45%, list=28%, signal=63%
KEGG_GLIOMA	58	0.5431943	1.4948604	0.00818714	0.04882156	1	2345	tags=36%, list=18%, signal=44%
WP_HISTONE_MODIFICATIONS	42	0.5833257	1.5444379	0.00850547	0.03324368	1	2671	tags=48%, list=21%, signal=60%
REACTOME_DEADENYLATION_OF_MRNA	25	0.6526737	1.5765604	0.00858896	0.02499901	1	1862	tags=48%, list=14%, signal=56%
PID_FGF_PATHWAY	40	0.5827238	1.4971877	0.00859951	0.04792865	1	2565	tags=48%, list=20%, signal=59%
BIOCARTA_VDR_PATHWAY	24	0.64823335	1.5428025	0.00863132	0.0335239	1	2652	tags=54%, list=20%, signal=68%
WP_FIBRIN_COMPLEMENT_RECEPTOR_3_SIGNALING_PATHWAY	31	0.6387191	1.576149	0.00872818	0.02505667	1	700	tags=23%, list=5%, signal=24%
REACTOME_ADHERENS_JUNCTIONS_INTERACTIONS	20	0.68504906	1.5886933	0.00875	0.02239664	0.998	1040	tags=25%, list=8%, signal=27%
PID_AURORA_A_PATHWAY	30	0.64097255	1.5756267	0.00880503	0.02499509	1	2345	tags=57%, list=18%, signal=69%
PID_REELIN_PATHWAY	23	0.6585152	1.5362965	0.00881612	0.03571114	1	2345	tags=52%, list=18%, signal=64%
KEGG_TYPE_I_DIABETES_MELLITUS	24	0.6577636	1.5679013	0.00884956	0.02697207	1	975	tags=13%, list=7%, signal=13%
REACTOME_RHO_GTPASES_ACTIVATE_NADPH_OXIDASES	22	0.6629279	1.5668676	0.00886076	0.02705706	1	1954	tags=45%, list=15%, signal=53%
REACTOME_ACTIVATION_OF_BH3_ONLY_PROTEINS	28	0.63873285	1.5534412	0.00886076	0.03065188	1	2345	tags=50%, list=18%, signal=61%
BIOCARTA_CARM_ER_PATHWAY	22	0.6597812	1.5528849	0.00890585	0.03069095	1	2626	tags=50%, list=20%, signal=63%
REACTOME_RHOC_GTPASE_CYCLE	71	0.5415504	1.5240865	0.0090703	0.03883452	1	2005	tags=38%, list=15%, signal=45%
REACTOME_THE_CITRIC_ACID_TCA_CYCLE_AND_RESPIRATORY_ELECTRON_TRANSPORT	169	0.44568408	1.3486179	0.00935551	0.13676111	1	1491	tags=24%, list=11%, signal=26%
REACTOME_TRANSCRIPTIONAL_REGULATION_OF_PLURIPOTENT_STEM_CELLS	15	0.70053166	1.5341275	0.00937082	0.0364124	1	1610	tags=40%, list=12%, signal=46%
KEGG_COLORECTAL_CANCER	58	0.56058383	1.5330306	0.00942285	0.03682677	1	2519	tags=43%, list=19%, signal=53%
WP_ENERGY_METABOLISM	41	0.59153473	1.5387992	0.00954654	0.03475285	1	2641	tags=46%, list=20%, signal=58%
REACTOME_APOPTOTIC_CLEAVAGE_OF_CELLULAR_PROTEINS	34	0.614001	1.5543278	0.00970874	0.0303727	1	2311	tags=44%, list=18%, signal=54%
BIOCARTA_P38MAPK_PATHWAY	32	0.5967033	1.5034094	0.00986437	0.04566533	1	2664	tags=47%, list=20%, signal=59%

Supplemental Table 4: DOWN-REGULATED PATHWAYS: BRQ-GMP vs Veh-GMP

Supplemental Table 4	SIZE	ES	NES	NOM p-val	FDR q-val	FWER p-val	RANK AT MAX	LEADING EDGE
REACTOME_SRP_DEPENDENT_COTRANSLATIONAL_PROTEIN_TARGETING_TO_MEMBRANE	107	-0.9337201	-3.5745528	0	0	0	480	tags=79%, list=4%, signal=82%
REACTOME_EUKARYOTIC_TRANSLATION_INITIATION	113	-0.9139136	-3.5224223	0	0	0	336	tags=65%, list=3%, signal=66%
REACTOME_EUKARYOTIC_TRANSLATION_ELONGATION	87	-0.9443803	-3.4750917	0	0	0	336	tags=80%, list=3%, signal=82%
REACTOME_INFLUENZA_INFECTION	147	-0.8656409	-3.4545207	0	0	0	359	tags=49%, list=3%, signal=50%
REACTOME_SELENOAMINO_ACID_METABOLISM	109	-0.9081095	-3.4260304	0	0	0	336	tags=61%, list=3%, signal=63%
WP_CYTOPLASMIC_RIBOSOMAL_PROTEINS	86	-0.9383369	-3.42471	0	0	0	336	tags=77%, list=3%, signal=78%
REACTOME_REGULATION_OF_EXPRESSION_OF_SLITS_AND_ROBOS	159	-0.8481666	-3.4163477	0	0	0	448	tags=48%, list=3%, signal=50%
KEGG_RIBOSOME	82	-0.9469042	-3.393256	0	0	0	336	tags=80%, list=3%, signal=82%
REACTOME_RESPONSE_OF_EIF2AK4_GCN2_TO_AMINO_ACID_DEFICIENCY	96	-0.9290957	-3.3891149	0	0	0	336	tags=72%, list=3%, signal=73%
REACTOME_CELLULAR_RESPONSE_TO_STARVATION	146	-0.8403084	-3.3638842	0	0	0	353	tags=48%, list=3%, signal=49%
REACTOME_NONSENSE_MEDIATED_DECAY_NMD	110	-0.9077806	-3.316824	0	0	0	336	tags=61%, list=3%, signal=62%
REACTOME_RRNA_PROCESSING	194	-0.8360649	-3.2801313	0	0	0	336	tags=37%, list=3%, signal=37%
REACTOME_TRANSLATION	285	-0.7796342	-3.2706668	0	0	0	596	tags=41%, list=5%, signal=42%
REACTOME_SIGNALING_BY_ROBO_RECEPTORS	197	-0.7768456	-3.2095714	0	0	0	448	tags=39%, list=3%, signal=40%
REACTOME_ACTIVATION_OF_THE_MRNA_UPON_BINDING_OF_THE_CAP_BINDING_COMPLEX_AND_EIFS_AND _SUBSEQUENT_BINDING_TO_43S	57	-0.8863238	-2.9747493	0	0	0	302	tags=58%, list=2%, signal=59%
REACTOME_METABOLISM_OF_AMINO_ACIDS_AND_DERIVATIVES	307	-0.6890312	-2.9287043	0	0	0	543	tags=28%, list=4%, signal=29%
REACTOME_NERVOUS_SYSTEM_DEVELOPMENT	455	-0.5478396	-2.790151	0	0	0	448	tags=19%, list=3%, signal=19%
REACTOME_UNFOLDED_PROTEIN_RESPONSE_UPR	85	-0.6221827	-2.296202	0	0	0	1202	tags=39%, list=9%, signal=42%
KEGG_PROTEIN_EXPORT	23	-0.8212944	-2.262725	0	4.10E-05	0.001	542	tags=57%, list=4%, signal=59%
REACTOME_N_GLYCAN_TRIMMING_IN_THE_ER_AND_CALNEXIN_CALRETICULIN_CYCLE	35	-0.7286229	-2.2376773	0	1.59E-04	0.004	548	tags=29%, list=4%, signal=30%
WP_PHOTODYNAMIC_THERAPYINDUCED_UNFOLDED_PROTEIN_RESPONSE	26	-0.7999774	-2.1983676	0	1.91E-04	0.005	188	tags=31%, list=1%, signal=31%
REACTOME_CALNEXIN_CALRETICULIN_CYCLE	26	-0.7760189	-2.175563	0	2.55E-04	0.007	548	tags=31%, list=4%, signal=32%
WP_CHOLESTEROL_BIOSYNTHESIS_PATHWAY	15	-0.8405249	-2.0575922	0	0.00171069	0.048	1803	tags=87%, list=14%, signal=100%
REACTOME_RRNA_MODIFICATION_IN_THE_NUCLEUS_AND_CYTOSOL	59	-0.6138612	-2.0286787	0	0.00266869	0.077	900	tags=22%, list=7%, signal=24%
HALLMARK_UNFOLDED_PROTEIN_RESPONSE	110	-0.5509399	-2.0080414	0	0.00330801	0.098	1008	tags=31%, list=8%, signal=33%
REACTOME_CHOLESTEROL_BIOSYNTHESIS	24	-0.7116122	-1.99994	0	0.00355584	0.11	1803	tags=67%, list=14%, signal=77%
REACTOME_AUF1_HNRNP_D0_BINDS_AND_DESTABILIZES_MRNA	53	-0.5935123	-1.9796346	0	0.00456288	0.144	626	tags=32%, list=5%, signal=34%
REACTOME_HEDGEHOG_LIGAND_BIOGENESIS	59	-0.5822507	-1.9724836	0	0.00496464	0.156	846	tags=31%, list=7%, signal=32%
REACTOME_IRE1ALPHA_ACTIVATES_CHAPERONES	48	-0.6248173	-1.9493847	0	0.00667388	0.219	1085	tags=44%, list=8%, signal=48%
REACTOME_DEFECTIVE_CFTR_CAUSES_CYSTIC_FIBROSIS	60	-0.5674661	-1.9499	0	0.00687689	0.218	846	tags=30%, list=7%, signal=32%
HALLMARK_MTORC1_SIGNALING	197	-0.466544	-1.9296147	0	0.00811426	0.264	778	tags=24%, list=6%, signal=26%
REACTOME_COLLAGEN_BIOSYNTHESIS_AND_MODIFYING_ENZYMES	36	-0.6151813	-1.9136735	0	0.00946909	0.311	591	tags=19%, list=5%, signal=20%
REACTOME_PROTEIN_METHYLATION	17	-0.7620436	-1.892931	0	0.01187918	0.386	1516	tags=65%, list=12%, signal=73%
WP_GLYCOSYLATION_AND_RELATED_CONGENITAL_DEFECTS	25	-0.6372768	-1.8443274	0	0.01951279	0.559	1542	tags=52%, list=12%, signal=59%
REACTOME_PERK_REGULATES_GENE_EXPRESSION	28	-0.616363	-1.8409991	0	0.01959873	0.567	1202	tags=39%, list=9%, signal=43%
REACTOME_ABC_TRANSPORTER_DISORDERS	68	-0.538091	-1.822298	0	0.02282162	0.635	846	tags=26%, list=7%, signal=28%
REACTOME_ASYMMETRIC_LOCALIZATION_OF_PCP_PROTEINS	60	-0.5070729	-1.7386498	0	0.04616153	0.868	626	tags=23%, list=5%, signal=24%
WP_CHOLESTEROL_METABOLISM_INCLUDES_BOTH_BLOCH_AND_KANDUTSCHRUSSELL_PATHWAYS	43	-0.5433023	-1.7341737	0	0.04661391	0.879	1081	tags=40%, list=8%, signal=43%
REACTOME_DEGRADATION_OF_DVL	55	-0.5113286	-1.7067629	0	0.05377228	0.925	626	tags=25%, list=5%, signal=27%
REACTOME_DECTIN_1_MEDIATED_NONCANONICAL_NF_KB_SIGNALING	60	-0.4965798	-1.7075714	0	0.05469405	0.925	626	tags=23%, list=5%, signal=24%
REACTOME_DEGRADATION_OF_AXIN	54	-0.5058331	-1.6582625	0	0.07632586	0.98	626	tags=26%, list=5%, signal=27%
REACTOME_THE_ROLE_OF_GTSE1_IN_G2_M_PROGRESSION_AFTER_G2_CHECKPOINT	68	-0.4719695	-1.6367961	0	0.08761337	0.986	626	tags=25%, list=5%, signal=26%
REACTOME_ANTIGEN_PROCESSING_CROSS_PRESENTATION	97	-0.4426257	-1.6217774	0	0.09364338	0.992	626	tags=23%, list=5%, signal=24%
HALLMARK_MYC_TARGETS_V1	200	-0.3946473	-1.6132886	0	0.09800789	0.994	846	tags=23%, list=7%, signal=24%
REACTOME_REGULATION_OF_MRNA_STABILITY_BY_PROTEINS_THAT_BIND_AU_RICH_ELEMENTS	84	-0.4230844	-1.5156132	0	0.13667181	1	846	tags=25%, list=7%, signal=27%
REACTOME_ASPARAGINE_N_LINKED_GLYCOSYLATION	267	-0.3662106	-1.512116	0	0.13782023	1	1250	tags=24%, list=10%, signal=26%
REACTOME_ER_QUALITY_CONTROL_COMPARTMENT_ERQC	21	-0.661042	-1.811263	0.00442478	0.02481808	0.667	548	tags=24%, list=4%, signal=25%
REACTOME_REGULATION_OF_RAS_BY_GAPS	67	-0.4395101	-1.5204958	0.00680272	0.13662735	1	626	tags=21%, list=5%, signal=22%
REACTOME_REGULATION_OF_HMOX1_EXPRESSION_AND_ACTIVITY	64	-0.4420617	-1.4988371	0.00806452	0.14480956	1	650	tags=25%, list=5%, signal=26%
REACTOME_ABC_FAMILY_PROTEINS_MEDIATED_TRANSPORT	93	-0.420309	-1.5309639	0.00847458	0.13580425	1	846	tags=23%, list=7%, signal=24%
REACTOME_ASSEMBLY_OF_THE_PRE_REPLICATIVE_COMPLEX	67	-0.4465702	-1.5240201	0.00869565	0.13811071	1	1012	tags=25%, list=8%, signal=27%
REACTOME_HEDGEHOG_ON_STATE	78	-0.4101905	-1.4664121	0.00892857	0.16049236	1	689	tags=21%, list=5%, signal=22%

Supplemental Table 5: UP-REGULATED PATHWAYS: BRQ-GN vs Veh-GN

Supplemental Table 5	SIZE	ES	NES	NOM p-val	FDR q-val	FWER p-val	RANK AT MAX	LEADING EDGE
KEGG_LEUKOCYTE_TRANSENDOTHELIAL_MIGRATION	97	0.7164845	1.934291	0	0.00313839	0.003	1209	tags=32%, list=9%, signal=35%
HALLMARK_CHOLESTEROL_HOMEOSTASIS	69	0.7240756	1.8655325	0	0.00974144	0.018	1213	tags=29%, list=9%, signal=32%
REACTOME_NEUTROPHIL_DEGRANULATION	418	0.5996581	1.8433788	0	0.01125359	0.032	1337	tags=33%, list=10%, signal=36%
HALLMARK_EPITHELIAL_MESENCHYMAL_TRANSITION	149	0.64361894	1.8185018	0	0.0158909	0.061	1009	tags=11%, list=7%, signal=12%
PID_TXA2PATHWAY	54	0.71949303	1.8154807	0	0.01372754	0.064	1092	tags=35%, list=8%, signal=38%
REACTOME_CELL_JUNCTION_ORGANIZATION	61	0.7124767	1.8080487	0	0.01507272	0.085	1107	tags=20%, list=8%, signal=21%
REACTOME_SMOOTH_MUSCLE_CONTRACTION	31	0.787712	1.795267	0	0.01659129	0.107	1776	tags=39%, list=13%, signal=44%
PID_EPHA_FWDPATHWAY	29	0.8166542	1.792563	0	0.01606945	0.115	1092	tags=31%, list=8%, signal=34%
REACTOME_EXTRACELLULAR_MATRIX_ORGANIZATION	212	0.61287975	1.7896739	0	0.01486119	0.118	1504	tags=12%, list=11%, signal=14%
REACTOME_DEGRADATION_OF_THE_EXTRACELLULAR_MATRIX	89	0.6632536	1.7740549	0	0.02120122	0.183	1504	tags=16%, list=11%, signal=18%
PID_RAS_PATHWAY	29	0.79789245	1.7671517	0	0.02282877	0.216	797	tags=24%, list=6%, signal=26%
REACTOME_G_ALPHA_Q_SIGNALLING_EVENTS	138	0.62513083	1.7564623	0	0.02527596	0.258	2443	tags=27%, list=18%, signal=32%
REACTOME_SIGNALLING_TO_RAS	16	0.8846146	1.7543879	0	0.02451923	0.27	889	tags=44%, list=6%, signal=47%
HALLMARK_APICAL_JUNCTION	165	0.614069	1.75273	0	0.02373174	0.278	1545	tags=18%, list=11%, signal=20%
KEGG_REGULATION_OF_ACTIN_CYTOSKELETON	177	0.6100955	1.7449411	0	0.02660599	0.32	2094	tags=29%, list=15%, signal=34%
REACTOME_SIGNALING_BY_GPCR	421	0.561979	1.7425723	0	0.02654481	0.334	2483	tags=24%, list=18%, signal=28%
KEGG_COMPLEMENT_AND_COAGULATION_CASCADES	40	0.7365309	1.7396641	0	0.02681227	0.355	995	tags=13%, list=7%, signal=13%
REACTOME_ANTIMICROBIAL_PEPTIDES	30	0.7797465	1.7377787	0	0.02652975	0.37	458	tags=27%, list=3%, signal=28%
REACTOME_RAC1_GTPASE_CYCLE	174	0.60602355	1.7339098	0	0.02713607	0.394	1865	tags=29%, list=14%, signal=33%
KEGG_FC_GAMMA_R_MEDIATED_PHAGOCYTOSIS	88	0.6506446	1.7312739	0	0.02732647	0.412	1209	tags=30%, list=9%, signal=32%
REACTOME_PLATELET_ACTIVATION_SIGNALING_AND_AGGREGATION	231	0.5847637	1.7286787	0	0.02725101	0.428	1711	tags=29%, list=12%, signal=32%
REACTOME_GENERATION_OF_SECOND_MESSENGER_MOLECULES	27	0.7940304	1.7246616	0	0.02721448	0.454	1226	tags=26%, list=9%, signal=28%
REACTOME_SIGNALING_BY_VEGF	103	0.63331574	1.7156466	0	0.03092491	0.508	1575	tags=29%, list=11%, signal=33%
KEGG_CHEMOKINE_SIGNALING_PATHWAY	152	0.60798025	1.7147543	0	0.03037955	0.512	1947	tags=35%, list=14%, signal=40%
REACTOME_FCGAMMA_RECEPTOR_FCGR_DEPENDENT_PHAGOCYTOSIS	89	0.64490354	1.711823	0	0.02949776	0.53	1227	tags=30%, list=9%, signal=33%
KEGG_FOCAL_ADHESION	168	0.59190226	1.7094609	0	0.02966014	0.548	1398	tags=20%, list=10%, signal=22%
HALLMARK_COAGULATION	110	0.6194446	1.7056173	0	0.02993559	0.576	1824	tags=20%, list=13%, signal=23%
KEGG_DRUG_METABOLISM_CYTOCHROME_P450	28	0.76167256	1.6923105	0	0.03487949	0.661	471	tags=7%, list=3%, signal=7%
REACTOME_MUSCLE_CONTRACTION	131	0.6041764	1.675669	0	0.04089837	0.748	1332	tags=14%, list=10%, signal=15%
HALLMARK_COMPLEMENT	175	0.585876	1.6727974	0	0.04098331	0.763	1433	tags=22%, list=10%, signal=25%
PID_LYSOPHOSPHOLIPID_PATHWAY	57	0.667878	1.67026	0	0.04056428	0.783	1209	tags=26%, list=9%, signal=29%
PID_CXCR4_PATHWAY	96	0.622639	1.6687378	0	0.04062139	0.792	2159	tags=39%, list=16%, signal=45%
REACTOME_CELL_CELL_COMMUNICATION	93	0.62253857	1.6554124	0	0.04681785	0.856	1575	tags=22%, list=11%, signal=24%
REACTOME_EPH_EPHRIN_SIGNALING	80	0.625491	1.6495496	0	0.04653272	0.891	1313	tags=24%, list=10%, signal=26%
REACTOME_PARASITE_INFECTION	63	0.65102327	1.6398162	0	0.05127881	0.921	1146	tags=29%, list=8%, signal=31%
REACTOME_PI_METABOLISM	81	0.6216375	1.6349881	0	0.05334614	0.938	2182	tags=33%, list=16%, signal=39%
HALLMARK_INTERFERON_GAMMA_RESPONSE	189	0.5538499	1.6192564	0	0.05646509	0.976	2252	tags=38%, list=16%, signal=45%
HALLMARK_KRAS_SIGNALING_UP	163	0.5493703	1.585623	0	0.0779572	0.997	1274	tags=18%, list=9%, signal=20%
REACTOME_G_ALPHA_12_13_SIGNALLING_EVENTS	72	0.6147129	1.5733299	0	0.08580992	0.999	2148	tags=33%, list=16%, signal=39%
REACTOME_RHO_GTPASE_CYCLE	426	0.49364114	1.5330615	0	0.11494865	1	1769	tags=23%, list=13%, signal=26%
REACTOME_G_ALPHA_I_SIGNALLING_EVENTS	181	0.5057403	1.4760382	0	0.16415307	1	2296	tags=22%, list=17%, signal=26%
REACTOME_CDC42_GTPASE_CYCLE	146	0.5485071	1.542045	0.00146413	0.10888727	1	1469	tags=25%, list=11%, signal=27%
PID_PDGFRB_PATHWAY	126	0.5831861	1.6069478	0.00146628	0.06312773	0.985	1405	tags=32%, list=10%, signal=35%
REACTOME_CLASS_A_1_RHODOPSIN_LIKE_RECEPTORS	164	0.5246208	1.5017902	0.00149477	0.14035746	1	1573	tags=11%, list=11%, signal=12%
PID_TCR_PATHWAY	62	0.6552091	1.6299314	0.0015361	0.052831	0.954	2387	tags=39%, list=17%, signal=47%
REACTOME_NRAGE_SIGNALS_DEATH_THROUGH_JNK	53	0.64173096	1.5944878	0.0015625	0.07213771	0.995	2064	tags=28%, list=15%, signal=33%
REACTOME_RESPONSE_TO_ELEVATED_PLATELET_CYTOSOLIC_CA2	112	0.5491434	1.4956965	0.0015748	0.14561155	1	1838	tags=27%, list=13%, signal=31%
PID_RAC1_PATHWAY	52	0.6648724	1.651511	0.00158228	0.04591709	0.881	1405	tags=29%, list=10%, signal=32%
PID_ERBB1_INTERNALIZATION_PATHWAY	37	0.7320936	1.6972934	0.00166113	0.03291164	0.632	1209	tags=30%, list=9%, signal=33%
PID_P38_ALPHA_BETA_PATHWAY	30	0.7417454	1.6602015	0.00168634	0.04529069	0.834	1226	tags=27%, list=9%, signal=29%
KEGG_GLYCEROLIPID_METABOLISM	41	0.7144673	1.676833	0.00169205	0.04114628	0.739	1379	tags=27%, list=10%, signal=30%
REACTOME_FORMATION_OF_THE_CORNIFIED_ENVELOPE	30	0.75408953	1.6783766	0.00170068	0.04134559	0.727	1045	tags=13%, list=8%, signal=14%
REACTOME_NICOTINATE_METABOLISM	25	0.75418293	1.6714622	0.00171821	0.04104156	0.772	1486	tags=32%, list=11%, signal=36%
REACTOME_KERATINIZATION	30	0.75408953	1.7118679	0.00172414	0.03063229	0.53	1045	tags=13%, list=8%, signal=14%
REACTOME_SIGNALING_BY_ERYTHROPOIETIN	24	0.7701799	1.6742396	0.00172712	0.04095612	0.756	1684	tags=42%, list=12%, signal=47%
PID_RHOA_REG_PATHWAY	45	0.69274896	1.6546553	0.0017331	0.04632526	0.862	1527	tags=33%, list=11%, signal=37%
BIOCARTA_PAR1_PATHWAY	18	0.8195235	1.6494299	0.00177305	0.04569681	0.892	1154	tags=50%, list=8%, signal=55%
REACTOME_RAP1_SIGNALLING	15	0.85796684	1.6627856	0.00180505	0.0441623	0.822	1341	tags=60%, list=10%, signal=66%
REACTOME_RHO_GTPASE_EFFECTORS	263	0.47513142	1.4157305	0.00275482	0.21345559	1	1769	tags=24%, list=13%, signal=27%
REACTOME_GPCR_LIGAND_BINDING	218	0.50171816	1.4809793	0.00277008	0.16109689	1	1573	tags=11%, list=11%, signal=13%
KEGG_MAPK_SIGNALING_PATHWAY	219	0.4764886	1.3996041	0.00283688	0.23250449	1	2094	tags=25%, list=15%, signal=29%
REACTOME_LEISHMANIA_INFECTION	189	0.5013053	1.4554532	0.00286533	0.18748923	1	1948	tags=28%, list=14%, signal=32%
REACTOME_BIOLOGICAL_OXIDATIONS	126	0.5563073	1.5304793	0.00289855	0.11604548	1	2412	tags=19%, list=18%, signal=23%
HALLMARK_HYPOXIA	173	0.49859503	1.4430792	0.00296296	0.19642772	1	1817	tags=29%, list=13%, signal=34%
KEGG_ERBB_SIGNALING_PATHWAY	76	0.57946056	1.5279512	0.00314465	0.11728464	1	2305	tags=37%, list=17%, signal=44%
REACTOME_ASSEMBLY_OF_COLLAGEN_FIBRILS_AND_OTHER_MULTIMERIC_STRUCTURES	40	0.7318018	1.7261333	0.00314961	0.0276543	0.445	1185	tags=10%, list=9%, signal=11%
REACTOME_SYNTHESIS_OF_PIPS_AT_THE_PLASMA_MEMBRANE	52	0.64814556	1.6117606	0.00327332	0.06016732	0.984	1515	tags=31%, list=11%, signal=34%
KEGG_FC_EPSILON_RI_SIGNALING_PATHWAY	65	0.6379839	1.6186225	0.00335008	0.05612093	0.977	1209	tags=29%, list=9%, signal=32%
PID_FGF_PATHWAY	43	0.70426995	1.7074075	0.003367	0.02976553	0.56	1163	tags=30%, list=8%, signal=33%
PID_ECADHERIN_STABILIZATION_PATHWAY	38	0.6692254	1.5828494	0.003367	0.07783977	0.999	1129	tags=29%, list=8%, signal=31%
REACTOME_SIGNALLING_TO_ERKS	29	0.72841823	1.615351	0.00338983	0.05782375	0.981	1066	tags=45%, list=8%, signal=48%
PID_INTEGRIN2_PATHWAY	21	0.810814	1.7045716	0.00341297	0.02953537	0.586	995	tags=33%, list=7%, signal=36%
REACTOME_RHO_GTPASES_ACTIVATE_WASPS_AND_WAVES	34	0.72555	1.6535542	0.0034188	0.04519227	0.871	1146	tags=35%, list=8%, signal=38%
REACTOME_PEPTIDE_LIGAND_BINDING_RECEPTORS	93	0.58294487	1.564694	0.00468019	0.09233984	1	478	tags=9%, list=3%, signal=9%
REACTOME_PHASE_II_CONJUGATION_OF_COMPOUNDS	63	0.62679225	1.6015633	0.00478469	0.06594141	0.993	2242	tags=17%, list=16%, signal=21%
REACTOME_ONCOGENIC_MAPK_SIGNALING	75	0.5834509	1.5104296	0.00494234	0.13161702	1	2094	tags=39%, list=15%, signal=45%
REACTOME_FCERI_MEDIATED_MAPK_ACTIVATION	38	0.6791834	1.590199	0.00510204	0.07551187	0.996	1226	tags=29%, list=9%, signal=32%
REACTOME_EPHB_MEDIATED_FORWARD_SIGNALING	38	0.6873157	1.5864042	0.00513699	0.07782194	0.996	1092	tags=24%, list=8%, signal=26%
HALLMARK_APICAL_SURFACE	32	0.7440226	1.6569725	0.0052356	0.04675512	0.848	1092	tags=16%, list=8%, signal=17%
KEGG_METABOLISM_OF_XENOBIOTICS_BY_CYTOCHROME_P450	26	0.7547864	1.6302524	0.00530035	0.05350729	0.954	2	tags=4%, list=0%, signal=4%
BIOCARTA_RAC1_PATHWAY	21	0.77414155	1.6309761	0.0053286	0.05376917	0.95	1154	tags=38%, list=8%, signal=42%
REACTOME_PD_1_SIGNALING	16	0.8209414	1.6052382	0.00549451	0.06297684	0.986	14	tags=6%, list=0%, signal=6%
KEGG_NICOTINATE_AND_NICOTINAMIDE_METABOLISM	21	0.8131919	1.6899097	0.00550459	0.03530935	0.673	771	tags=24%, list=6%, signal=25%
REACTOME_PHOSPHOLIPID_METABOLISM	183	0.4962532	1.442213	0.00585652	0.19541262	1	1898	tags=23%, list=14%, signal=26%
KEGG_T_CELL_RECEPTOR_SIGNALING_PATHWAY	99	0.5920735	1.5842117	0.00592593	0.07847575	0.999	1969	tags=29%, list=14%, signal=34%
PID_AMB2_NEUTROPHILS_PATHWAY	35	0.7006793	1.631854	0.00672269	0.0549048	0.95	1092	tags=34%, list=8%, signal=37%
PID_MAPK_TRK_PATHWAY	31	0.7104418	1.5834157	0.00699301	0.07830712	0.999	2094	tags=58%, list=15%, signal=68%
REACTOME_RHO_GTPASES_ACTIVATE_PAKS	20	0.80514693	1.6538118	0.00704225	0.04591896	0.867	1338	tags=35%, list=10%, signal=39%
HALLMARK_ESTROGEN_RESPONSE_LATE	162	0.50164574	1.4431142	0.00706215	0.19766794	1	1664	tags=18%, list=12%, signal=20%
REACTOME_CELL_EXTRACELLULAR_MATRIX_INTERACTIONS	15	0.8198675	1.6195134	0.00733945	0.057055	0.976	546	tags=40%, list=4%, signal=42%
REACTOME_INTERFERON_GAMMA_SIGNALING	70	0.57747567	1.5215628	0.0078125	0.12132271	1	2591	tags=41%, list=19%, signal=51%
BIOCARTA_MAPK_PATHWAY	80	0.6031359	1.5823535	0.00783699	0.07737227	0.999	2349	tags=45%, list=17%, signal=54%
REACTOME_RHO_GTPASES_ACTIVATE_NADPH_OXIDASES	22	0.78153265	1.6368239	0.00827815	0.05287379	0.933	889	tags=41%, list=6%, signal=44%
REACTOME_APOPTOTIC_CLEAVAGE_OF_CELLULAR_PROTEINS	35	0.6845755	1.564909	0.00841751	0.09330522	1	1065	tags=29%, list=8%, signal=31%
REACTOME_ACTIVATION_OF_MATRIX_METALLOPROTEINASES	20	0.7833858	1.6313832	0.00851789	0.05434502	0.95	148	tags=20%, list=1%, signal=20%
PID_EPHB_FWD_PATHWAY	37	0.70919424	1.6472032	0.00856164	0.04633215	0.896	1575	tags=32%, list=11%, signal=37%
BIOCARTA_GH_PATHWAY	24	0.71531147	1.519106	0.00859107	0.12368738	1	1747	tags=42%, list=13%, signal=48%
REACTOME_INTERACTION_BETWEEN_L1_AND_ANKYRINS	21	0.7687661	1.6210817	0.00860585	0.05665061	0.974	1977	tags=19%, list=14%, signal=22%
REACTOME_GLUTATHIONE_CONJUGATION	28	0.7042233	1.5688922	0.00880282	0.08965283	1	1586	tags=18%, list=12%, signal=20%
REACTOME_TRIGLYCERIDE_METABOLISM	25	0.74700123	1.6251407	0.00884956	0.05482911	0.965	1652	tags=40%, list=12%, signal=45%
PID_CDC42_PATHWAY	68	0.58169645	1.4947695	0.00949367	0.1458197	1	1444	tags=26%, list=11%, signal=29%
REACTOME_NCAM_SIGNALING_FOR_NEURITE_OUT_GROWTH	51	0.62672454	1.5261468	0.00972447	0.11864248	1	2622	tags=22%, list=19%, signal=27%
REACTOME_COLLAGEN_DEGRADATION	43	0.6515085	1.5484486	0.00980392	0.10524058	1	43	tags=5%, list=0%, signal=5%

Supplemental Table 6	SIZE	ES	NES	NOM p-val	FDR q-val	FWER p-val	RANK AT MAX	LEADING EDGE
REACTOME_EUKARYOTIC_TRANSLATION_INITIATION	113	-0.861016	-2.5609612	0	0	0	1553	tags=84%, list=11%, signal=94%
REACTOME_SRP_DEPENDENT_COTRANSLATIONAL_PROTEIN_TARGETING_TO_MEMBRANE	107	-0.8668971	-2.5345783	0	0	0	1152	tags=77%, list=8%, signal=83%
REACTOME_SELENOAMINO_ACID_METABOLISM	110	-0.8411351	-2.5052927	0	0	0	1120	tags=65%, list=8%, signal=70%
REACTOME_INFLUENZA_INFECTION	147	-0.8063818	-2.5045288	0	0	0	1170	tags=61%, list=9%, signal=65%
KEGG_RIBOSOME	82	-0.8774783	-2.499195	0	0	0	1120	tags=84%, list=8%, signal=91%
REACTOME_EUKARYOTIC_TRANSLATION_ELONGATION	87	-0.8755678	-2.4847841	0	0	0	1120	tags=83%, list=8%, signal=90%
REACTOME_TRANSLATION	285	-0.7501218	-2.4736266	0	0	0	1553	tags=52%, list=11%, signal=57%
REACTOME_RESPONSE_OF_EIF2AK4_GCN2_TO_AMINO_ACID_DEFICIENCY	96	-0.8494104	-2.4669185	0	0	0	1120	tags=76%, list=8%, signal=82%
REACTOME_REGULATION_OF_EXPRESSION_OF_SLITS_AND_ROBOS	162	-0.7868773	-2.4408035	0	0	0	1592	tags=64%, list=12%, signal=72%
REACTOME_RRNA_PROCESSING	195	-0.7646021	-2.418813	0	0	0	1553	tags=51%, list=11%, signal=57%
REACTOME_NONSENSE_MEDIATED_DECAY_NMD	110	-0.8135799	-2.3792336	0	0	0	1553	tags=75%, list=11%, signal=84%
REACTOME_CELLULAR_RESPONSE_TO_STARVATION	147	-0.758913	-2.346863	0	0	0	1553	tags=60%, list=11%, signal=67%
REACTOME_ACTIVATION_OF_THE_MRNA_UPON_BINDING_OF_THE_CAP_BINDING_COMPLEX_AND_EIFS_AND_SUBSEQUENT_BINDING_TO_43S	57	-0.8696287	-2.3355045	0	0	0	1553	tags=89%, list=11%, signal=100%
HALLMARK_MYC_TARGETS_V1	200	-0.7238857	-2.3241816	0	0	0	1951	tags=67%, list=14%, signal=77%
REACTOME_SIGNALING_BY_ROBO_RECEPTORS	202	-0.6940554	-2.2048593	0	0	0	1471	tags=51%, list=11%, signal=56%
REACTOME_METABOLISM_OF_AMINO_ACIDS_AND_DERIVATIVES	311	-0.6638078	-2.1921306	0	0	0	1592	tags=37%, list=12%, signal=41%
REACTOME_MRNA_SPLICING	185	-0.6872135	-2.177875	0	0	0	1967	tags=55%, list=14%, signal=63%
REACTOME_RESPIRATORY_ELECTRON_TRANSPORT_ATP_SYNTHESIS_BY_CHEMIOSMOTIC_COUPLING_AND_HEAT_PRODUCTION_BY_UNCOUPLING_PROTEINS	124	-0.7077466	-2.1283238	0	0	0	2163	tags=55%, list=16%, signal=64%
REACTOME_PROCESSING_OF_CAPPED_INTRON_CONTAINING_PRE_MRNA	236	-0.6500097	-2.0816872	0	0	0	1967	tags=49%, list=14%, signal=56%
REACTOME_RESPIRATORY_ELECTRON_TRANSPORT	101	-0.6925972	-2.0564792	0	0	0	1925	tags=50%, list=14%, signal=57%
KEGG_PARKINSONS_DISEASE	117	-0.6857938	-2.04176	0	0	0	1447	tags=47%, list=11%, signal=52%
REACTOME_THE_CITRIC_ACID_TCA_CYCLE_AND_RESPIRATORY_ELECTRON_TRANSPORT	171	-0.6402451	-2.0074384	0	4.34E-05	0.001	1968	tags=45%, list=14%, signal=52%
HALLMARK_OXIDATIVE_PHOSPHORYLATION	199	-0.6268606	-1.9860283	0	4.15E-05	0.001	1484	tags=43%, list=11%, signal=47%
KEGG_OXIDATIVE_PHOSPHORYLATION	117	-0.6671923	-1.9810016	0	3.98E-05	0.001	1447	tags=45%, list=11%, signal=50%
KEGG_SPLICEOSOME	122	-0.6617242	-1.9705527	0	1.17E-04	0.003	2288	tags=61%, list=17%, signal=72%
HALLMARK_MYC_TARGETS_V2	57	-0.7191074	-1.9290876	0	6.22E-04	0.017	1711	tags=44%, list=12%, signal=50%
KEGG_HUNTINGTONS_DISEASE	160	-0.6214435	-1.9148005	0	8.11E-04	0.023	2186	tags=51%, list=16%, signal=60%
REACTOME_CRISTAE_FORMATION	31	-0.7670196	-1.8607117	0	0.002931799	0.082	2163	tags=71%, list=16%, signal=84%
REACTOME_COMPLEX_I_BIOGENESIS	56	-0.6890822	-1.8354876	0	0.004882379	0.136	1925	tags=50%, list=14%, signal=58%
REACTOME_MITOCHONDRIAL_PROTEIN_IMPORT	63	-0.6724734	-1.8276519	0	0.00570634	0.163	1635	tags=44%, list=12%, signal=50%
REACTOME_BINDING_AND_UPTAKE_OF_LIGANDS_BY_SCAVENGER_RECEPTORS	36	-0.7150986	-1.7875823	0	0.011543506	0.317	1784	tags=25%, list=13%, signal=29%
KEGG_ALZHEIMERS_DISEASE	150	-0.5849877	-1.7823967	0	0.011890529	0.343	1447	tags=38%, list=11%, signal=42%
HALLMARK_E2F_TARGETS	200	-0.5499778	-1.753029	0	0.019265678	0.494	2091	tags=37%, list=15%, signal=42%
KEGG_CARDIAC_MUSCLE_CONTRACTION	58	-0.6504694	-1.7397833	0	0.02265231	0.554	1033	tags=28%, list=8%, signal=30%
BIOCARTA_PROTEASOME_PATHWAY	19	-0.7892262	-1.7273735	0	0.026834548	0.635	1793	tags=63%, list=13%, signal=73%
REACTOME_SYNTHESIS_OF_DNA	118	-0.5750705	-1.7169662	0	0.029716274	0.701	2126	tags=46%, list=15%, signal=54%
REACTOME_REGULATION_OF_MRNA_STABILITY_BY_PROTEINS_THAT_BIND_AU_RICH_ELEMENTS	85	-0.5834261	-1.6720952	0	0.048830353	0.904	2126	tags=52%, list=15%, signal=61%
REACTOME_SWITCHING_OF_ORIGINS_TO_A_POST_REPLICATIVE_STATE	89	-0.5888977	-1.668523	0	0.049824033	0.914	2126	tags=52%, list=15%, signal=61%
REACTOME_HOST_INTERACTIONS_OF_HIV_FACTORS	126	-0.5482371	-1.6647508	0	0.051343437	0.928	2243	tags=45%, list=16%, signal=54%
REACTOME_NEGATIVE_REGULATION_OF_NOTCH4_SIGNALING	54	-0.6297957	-1.6489433	0	0.05743918	0.956	2126	tags=54%, list=15%, signal=63%
REACTOME_DNA_REPLICATION	146	-0.5334984	-1.6271523	0	0.0674319	0.985	2013	tags=39%, list=15%, signal=45%
REACTOME_SARS_COV_INFECTIONS	148	-0.5342217	-1.6203835	0	0.07013419	0.988	1606	tags=39%, list=12%, signal=44%
REACTOME_DNA_REPLICATION_PRE_INITIATION	104	-0.5523534	-1.6104624	0	0.07024686	0.992	1896	tags=39%, list=14%, signal=45%
HALLMARK_G2M_CHECKPOINT	194	-0.5045474	-1.6060194	0	0.071259424	0.992	1866	tags=35%, list=14%, signal=40%
REACTOME_NERVOUS_SYSTEM_DEVELOPMENT	487	-0.4653095	-1.5991567	0	0.07562005	0.994	1553	tags=26%, list=11%, signal=29%
REACTOME_G2_M_CHECKPOINTS	141	-0.5230995	-1.5902766	0	0.07956751	0.997	2200	tags=37%, list=16%, signal=43%
REACTOME_APC_C_MEDIATED_DEGRADATION_OF_CELL_CYCLE_PROTEINS	86	-0.5415922	-1.5527455	0	0.10145661	0.999	2126	tags=45%, list=15%, signal=53%
REACTOME_HIV_INFECTION	222	-0.476989	-1.5169659	0	0.11587834	1	1632	tags=31%, list=12%, signal=34%
REACTOME_S_PHASE	159	-0.4775032	-1.491976	0	0.13338329	1	2386	tags=42%, list=17%, signal=50%
REACTOME_ABC_FAMILY_PROTEINS_MEDIATED_TRANSPORT	94	-0.5081672	-1.4824399	0	0.13936308	1	2126	tags=37%, list=15%, signal=44%
REACTOME_DISORDERS_OF_TRANSMEMBRANE_TRANSPORTERS	145	-0.4787316	-1.4721701	0	0.1421347	1	2219	tags=30%, list=16%, signal=36%
REACTOME_M_PHASE	357	-0.4319616	-1.4505732	0	0.1576273	1	2027	tags=29%, list=15%, signal=33%
REACTOME_CELL_CYCLE_CHECKPOINTS	261	-0.4236648	-1.390465	0	0.20867841	1	2084	tags=30%, list=15%, signal=34%
REACTOME_CELL_CYCLE_MITOTIC	496	-0.3927282	-1.3468442	0	0.2441862	1	2027	tags=27%, list=15%, signal=31%
REACTOME_FORMATION_OF_ATP_BY_CHEMIOSMOTIC_COUPLING	18	-0.8288731	-1.7833368	0.002267574	0.012141007	0.34	2163	tags=89%, list=16%, signal=105%
REACTOME_ABC_TRANSPORTER_DISORDERS	70	-0.5752536	-1.5916544	0.0025	0.079529166	0.997	2126	tags=43%, list=15%, signal=50%
REACTOME_MRNA_SPLICING_MINOR_PATHWAY	52	-0.6345361	-1.6841272	0.002512563	0.043033022	0.862	1807	tags=50%, list=13%, signal=57%
REACTOME_AUF1_HNRNP_D0_BINDS_AND_DESTABILIZES_MRNA	54	-0.6362101	-1.7240211	0.002557545	0.027187692	0.65	2126	tags=57%, list=15%, signal=68%
REACTOME_REGULATION_OF_HMOX1_EXPRESSION_AND_ACTIVITY	64	-0.5687773	-1.5696417	0.002564103	0.09134496	0.999	1407	tags=44%, list=10%, signal=49%
REACTOME_THE_ROLE_OF_GTSE1_IN_G2_M_PROGRESSION_AFTER_G2_CHECKPOINT	71	-0.5966849	-1.6520289	0.002617801	0.056670666	0.95	1896	tags=42%, list=14%, signal=49%
REACTOME_ORC1_REMOVAL_FROM_CHROMATIN	69	-0.6246299	-1.7051281	0.002624672	0.034084298	0.759	2126	tags=57%, list=15%, signal=67%
REACTOME_DECTIN_1_MEDIATED_NONCANONICAL_NF_KB_SIGNALING	60	-0.5892913	-1.6193573	0.00265252	0.069515094	0.988	2133	tags=50%, list=16%, signal=59%
REACTOME_REGULATION_OF_PTEN_STABILITY_AND_ACTIVITY	67	-0.5785998	-1.5742171	0.002659574	0.08781141	0.998	1896	tags=46%, list=14%, signal=53%
REACTOME_TRANSCRIPTION_COUPLED_NUCLEOTIDE_EXCISION_REPAIR_TC_NER	77	-0.5368074	-1.5140841	0.002793296	0.117595874	1	2328	tags=43%, list=17%, signal=51%
REACTOME_CYTOPROTECTION_BY_HMOX1	116	-0.5158236	-1.5432162	0.002941177	0.100447744	1	1490	tags=37%, list=11%, signal=41%
HALLMARK_UNFOLDED_PROTEIN_RESPONSE	111	-0.5519248	-1.6173009	0.002985075	0.06861457	0.99	1654	tags=37%, list=12%, signal=42%
REACTOME_PTEN_REGULATION	133	-0.5053533	-1.540273	0.003012048	0.10196963	1	1423	tags=35%, list=10%, signal=38%
REACTOME_HIV_LIFE_CYCLE	144	-0.450478	-1.3938993	0.003021148	0.20511097	1	1632	tags=26%, list=12%, signal=29%
REACTOME_MITOTIC_METAPHASE_AND_ANAPHASE	223	-0.4432003	-1.4242445	0.003412969	0.18318221	1	2084	tags=31%, list=15%, signal=36%
KEGG_PROTEIN_EXPORT	23	-0.7514619	-1.6885673	0.004555809	0.04183259	0.845	2247	tags=65%, list=16%, signal=78%
KEGG_PROTEASOME	42	-0.6465458	-1.6627342	0.004878049	0.051567577	0.931	2126	tags=62%, list=15%, signal=73%
REACTOME_RNA_POLYMERASE_II_TRANSCRIPTION_TERMINATION	65	-0.5873712	-1.6166179	0.004950495	0.0679217	0.99	1809	tags=45%, list=13%, signal=51%
REACTOME_CDT1_ASSOCIATION_WITH_THE_CDC6_ORC_ORIGIN_COMPLEX	58	-0.6027808	-1.6174737	0.005025126	0.06968576	0.989	2126	tags=52%, list=15%, signal=61%
REACTOME_MATURATION_OF_SARS_COV_2_SPIKE_PROTEIN	29	-0.6905243	-1.6417319	0.005050505	0.06010371	0.969	1328	tags=45%, list=10%, signal=50%
REACTOME_HEDGEHOG_LIGAND_BIOGENESIS	59	-0.6017358	-1.6258354	0.005063291	0.06701546	0.985	2126	tags=51%, list=15%, signal=60%
REACTOME_SUMOYLATION_OF_DNA_REPLICATION_PROTEINS	45	-0.6143332	-1.6113282	0.005063291	0.0705918	0.992	1786	tags=38%, list=13%, signal=43%
REACTOME_DEGRADATION_OF_AXIN	54	-0.5606728	-1.4784385	0.005249344	0.14100094	1	2126	tags=50%, list=15%, signal=59%
REACTOME_RRNA_MODIFICATION_IN_THE_NUCLEUS_AND_CYTOSOL	60	-0.5676805	-1.5287625	0.005347594	0.10861292	1	1489	tags=30%, list=11%, signal=33%
REACTOME_REGULATION_OF_RUNX2_EXPRESSION_AND_ACTIVITY	68	-0.5685437	-1.5512245	0.005390836	0.10145102	0.999	1896	tags=41%, list=14%, signal=48%
REACTOME_G1_S_DNA_DAMAGE_CHECKPOINTS	66	-0.5334764	-1.4552951	0.005509642	0.15430619	1	1896	tags=42%, list=14%, signal=49%
REACTOME_UCH_PROTEINASES	88	-0.5358867	-1.543611	0.005617978	0.101313315	1	1896	tags=39%, list=14%, signal=45%
REACTOME_NUCLEOTIDE_EXCISION_REPAIR	109	-0.4996934	-1.4931264	0.005633803	0.13324912	1	2342	tags=42%, list=17%, signal=50%
REACTOME_MITOCHONDRIAL_TRANSLATION	94	-0.5607279	-1.6369292	0.005665722	0.06254936	0.976	2015	tags=37%, list=15%, signal=43%
REACTOME_MITOTIC_G1_PHASE_AND_G1_S_TRANSITION	145	-0.4518994	-1.3889076	0.006116208	0.20973451	1	2151	tags=37%, list=16%, signal=43%
REACTOME_MITOTIC_G2_G2_M_PHASES	188	-0.4254198	-1.3328292	0.006756757	0.25401443	1	1815	tags=29%, list=13%, signal=33%
REACTOME_INTRACELLULAR_SIGNALING_BY_SECOND_MESSENGERS	271	-0.3867681	-1.2668557	0.007380074	0.31778604	1	1423	tags=22%, list=10%, signal=24%
REACTOME_INTERACTIONS_OF_REV_WITH_HOST_CELLULAR_PROTEINS	36	-0.6469389	-1.6338332	0.007444169	0.06338676	0.978	2480	tags=42%, list=18%, signal=51%
REACTOME_DEGRADATION_OF_DVL	55	-0.5946562	-1.5874388	0.007537689	0.08076895	0.997	1896	tags=49%, list=14%, signal=57%
REACTOME_DEGRADATION_OF_GLI1_BY_THE_PROTEASOME	57	-0.5750332	-1.5455338	0.0078125	0.100867294	0.999	1896	tags=47%, list=14%, signal=55%
REACTOME_REGULATION_OF_HSF1_MEDIATED_HEAT_SHOCK_RESPONSE	77	-0.5195742	-1.4732065	0.008108108	0.14211638	1	2637	tags=39%, list=19%, signal=48%
REACTOME_TRANSPORT_OF_MATURE_TRANSCRIPT_TO_CYTOPLASM	81	-0.5183928	-1.4832851	0.008241759	0.13975607	1	1809	tags=35%, list=13%, signal=40%
REACTOME_REGULATION_OF_INSULIN_LIKE_GROWTH_FACTOR_IGF_TRANSPORT_AND_UPTAKE_BY_INSULIN_LIKE_GROWTH_FACTOR_BINDING_PROTEINS_IGFBPS	75	-0.5393115	-1.5073025	0.008333334	0.12017395	1	1243	tags=23%, list=9%, signal=25%
REACTOME_PIWI_INTERACTING_RNA_PIRNA_BIOGENESIS	20	-0.7508026	-1.6548569	0.00952381	0.055866733	0.946	1440	tags=40%, list=10%, signal=45%
REACTOME_HSF1_ACTIVATION	27	-0.6883146	-1.6102238	0.00954654	0.06926607	0.992	1443	tags=37%, list=11%, signal=41%
REACTOME_SEPARATION_OF_SISTER_CHROMATIDS	180	-0.4358749	-1.3646924	0.009677419	0.2337185	1	2126	tags=32%, list=15%, signal=38%
REACTOME_ASYMMETRIC_LOCALIZATION_OF_PCP_PROTEINS	61	-0.5741949	-1.5277969	0.011019284	0.10835646	1	2126	tags=44%, list=15%, signal=52%
KEGG_RNA_DEGRADATION	55	-0.5601219	-1.5015616	0.011019284	0.1251207	1	2071	tags=42%, list=15%, signal=49%
KEGG_DNA_REPLICATION	35	-0.6322073	-1.5553936	0.011389522	0.101644866	0.999	1736	tags=43%, list=13%, signal=49%
REACTOME_RUNX1_REGULATES_TRANSCRIPTION_OF_GENES_INVOLVED_IN_DIFFERENTIATION_OF_HSCS	86	-0.527875	-1.5129621	0.011494253	0.11746818	1	2151	tags=41%, list=16%, signal=48%

Supplemental Table 6: DOWN-REGULATED PATHWAYS: BRQ-GN vs Veh-GN

REACTOME_COOPERATION_OF_PREFOLDIN_AND_TRIC_CCT_IN_ACTIN_AND_TUBULIN_FOLDING	28	-0.6381112	-1.5099748	0.011574074	0.119670704	1	1239	tags=29%, list=9%, signal=31%
REACTOME_VIRAL_MESSENGER_RNA_SYNTHESIS	43	-0.6025637	-1.536299	0.011990408	0.104504146	1	2649	tags=47%, list=19%, signal=57%
KEGG_N_GLYCAN_BIOSYNTHESIS	43	-0.585035	-1.4860065	0.012406948	0.1379605	1	1694	tags=33%, list=12%, signal=37%
REACTOME_SCF_SKP2_MEDIATED_DEGRADATION_OF_P27_P21	58	-0.5882057	-1.574863	0.0132626	0.088548586	0.998	1896	tags=48%, list=14%, signal=56%
REACTOME_SARS_COV_2_INFECTION	67	-0.568251	-1.5651019	0.013774104	0.09408773	0.999	1328	tags=37%, list=10%, signal=41%
REACTOME_FGFR2_ALTERNATIVE_SPLICING	24	-0.714372	-1.6484938	0.014184397	0.056560576	0.956	1440	tags=54%, list=10%, signal=60%
REACTOME_CROSS_PRESENTATION_OF_SOLUBLE_EXOGENOUS_ANTIGENS_ENDOSOMES	48	-0.5944954	-1.5486376	0.014184397	0.101475626	0.999	2126	tags=52%, list=15%, signal=61%
REACTOME_DEFECTIVE_CFTR_CAUSES_CYSTIC_FIBROSIS	60	-0.5864057	-1.5956422	0.015189873	0.07748186	0.996	2126	tags=48%, list=15%, signal=57%
REACTOME_TRANSLATION_OF_SARS_COV_2_STRUCTURAL_PROTEINS	49	-0.5812824	-1.5326757	0.015189873	0.10686377	1	1328	tags=43%, list=10%, signal=47%
REACTOME_CELLULAR_RESPONSE_TO_HYPOXIA	73	-0.5250921	-1.4742512	0.015345269	0.14332446	1	2145	tags=48%, list=16%, signal=57%
REACTOME_APC_C_CDH1_MEDIATED_DEGRADATION_OF_CDC20_AND_OTHER_APC_C_CDH1_TARGETED_PROTEINS_IN_LATE_MITOSIS_EARLY_G1	73	-0.5453634	-1.5089161	0.016216217	0.1196597	1	2126	tags=47%, list=15%, signal=55%
REACTOME_DNA_STRAND_ELONGATION	32	-0.6456295	-1.547988	0.016627079	0.100741185	0.999	2354	tags=50%, list=17%, signal=60%
REACTOME_NEDDYLATION	221	-0.4006723	-1.2869934	0.017301038	0.2976214	1	2181	tags=29%, list=16%, signal=34%
REACTOME_POTENTIAL_THERAPEUTICS_FOR_SARS	82	-0.51764	-1.4690243	0.01734104	0.14442268	1	1606	tags=40%, list=12%, signal=45%
REACTOME_FCERI_MEDIATED_NF_KB_ACTIVATION	87	-0.4919767	-1.4077125	0.017857144	0.19409935	1	1896	tags=38%, list=14%, signal=44%
REACTOME_DUAL_INCISION_IN_TC_NER	64	-0.5383188	-1.4734195	0.017902814	0.1431468	1	1704	tags=36%, list=12%, signal=41%
REACTOME_HEDGEHOG_OFF_STATE	104	-0.4821051	-1.4172151	0.018404908	0.18854682	1	1407	tags=25%, list=10%, signal=28%
REACTOME_RECOGNITION_OF_DNA_DAMAGE_BY_PCNA_CONTAINING_REPLICATION_COMPLEX	30	-0.6413103	-1.5489354	0.019002376	0.10245636	0.999	1282	tags=37%, list=9%, signal=40%
KEGG_UBIQUITIN_MEDIATED_PROTEOLYSIS	129	-0.4479283	-1.3565503	0.020231213	0.24039702	1	2013	tags=33%, list=15%, signal=38%
BIOCARTA_ERAD_PATHWAY	19	-0.7416878	-1.5870174	0.02027027	0.079956435	0.997	1328	tags=53%, list=10%, signal=58%
PID_HIF1A_PATHWAY	19	-0.7102327	-1.5471733	0.020930232	0.10043418	0.999	2003	tags=53%, list=15%, signal=62%
REACTOME_METABOLISM_OF_POLYAMINES	57	-0.5409756	-1.4326944	0.021333333	0.17337397	1	1896	tags=47%, list=14%, signal=55%
REACTOME_HIV_ELONGATION_ARREST_AND_RECOVERY	32	-0.6385186	-1.554748	0.021582734	0.10090577	0.999	1481	tags=44%, list=11%, signal=49%
BIOCARTA_EIF_PATHWAY	16	-0.7726824	-1.5766196	0.021951219	0.08818027	0.998	1631	tags=75%, list=12%, signal=85%
REACTOME_INTERLEUKIN_1_SIGNALING	98	-0.4816415	-1.396915	0.022012578	0.20360172	1	1407	tags=33%, list=10%, signal=36%
REACTOME_STABILIZATION_OF_P53	56	-0.5739636	-1.5307473	0.022613065	0.107806355	1	1896	tags=46%, list=14%, signal=54%
REACTOME_CELLULAR_RESPONSE_TO_CHEMICAL_STRESS	146	-0.41674	-1.2890155	0.023569023	0.2974365	1	1490	tags=34%, list=11%, signal=38%
REACTOME_TNFR2_NON_CANONICAL_NF_KB_PATHWAY	95	-0.4729902	-1.3491366	0.024193548	0.24207571	1	2133	tags=32%, list=16%, signal=37%
REACTOME_INTERACTIONS_OF_VPR_WITH_HOST_CELLULAR_PROTEINS	35	-0.5981895	-1.4682012	0.024813896	0.14420216	1	3015	tags=54%, list=22%, signal=69%
REACTOME_PROCESSING_OF_CAPPED_INTRONLESS_PRE_MRNA	28	-0.653962	-1.5275117	0.025125628	0.10742713	1	1767	tags=50%, list=13%, signal=57%
REACTOME_GENE_SILENCING_BY_RNA	87	-0.4892694	-1.4214619	0.025352113	0.18564555	1	2213	tags=28%, list=16%, signal=33%
REACTOME_CELLULAR_RESPONSE_TO_HEAT_STRESS	96	-0.4610886	-1.3457646	0.025352113	0.24450763	1	2683	tags=39%, list=20%, signal=48%
REACTOME_ORGANELLE_BIOGENESIS_AND_MAINTENANCE	280	-0.3778667	-1.2354454	0.025362318	0.34243593	1	2683	tags=30%, list=20%, signal=36%
REACTOME_FORMATION_OF_TC_NER_PRE_INCISION_COMPLEX	52	-0.5523957	-1.4613882	0.02676399	0.15058315	1	2328	tags=48%, list=17%, signal=58%
REACTOME_ASPARAGINE_N_LINKED_GLYCOSYLATION	275	-0.3750697	-1.2346565	0.027149322	0.34252074	1	1725	tags=22%, list=13%, signal=25%
REACTOME_HOMOLOGY_DIRECTED_REPAIR	110	-0.4306255	-1.2923319	0.028301887	0.29623154	1	2371	tags=26%, list=17%, signal=32%
REACTOME_TRANSCRIPTIONAL_REGULATION_BY_THE_AP_2_TFAP2_FAMILY_OF_TRANSCRIPTION_FACTORS	29	-0.6252137	-1.4867082	0.029268293	0.13845117	1	1764	tags=31%, list=13%, signal=36%
REACTOME_SUMOYLATION_OF_RNA_BINDING_PROTEINS	45	-0.5499868	-1.4200047	0.029569892	0.18591586	1	3019	tags=47%, list=22%, signal=60%
REACTOME_DNA_DOUBLE_STRAND_BREAK_REPAIR	139	-0.4333729	-1.3114326	0.030487806	0.27655533	1	2211	tags=25%, list=16%, signal=30%
REACTOME_TP53_REGULATES_TRANSCRIPTION_OF_DNA_REPAIR_GENES	61	-0.5338938	-1.4402924	0.03076923	0.1665876	1	2262	tags=39%, list=16%, signal=47%
REACTOME_ANTIGEN_PROCESSING_UBIQUITINATION_PROTEASOME_DEGRADATION	286	-0.381839	-1.2498502	0.030927835	0.33258426	1	2145	tags=29%, list=16%, signal=34%
REACTOME_CLEC7A_DECTIN_1_SIGNALING	97	-0.4763679	-1.3763069	0.031073445	0.22118993	1	2145	tags=42%, list=16%, signal=50%
REACTOME_DOWNSTREAM_SIGNALING_EVENTS_OF_B_CELL_RECEPTOR_BCR	80	-0.4784466	-1.3558244	0.032258064	0.24026223	1	2126	tags=44%, list=15%, signal=51%
REACTOME_REGULATION_OF_RAS_BY_GAPS	67	-0.489654	-1.36475	0.032828283	0.2351648	1	1896	tags=40%, list=14%, signal=47%
REACTOME_REGULATION_OF_RUNX3_EXPRESSION_AND_ACTIVITY	55	-0.5233873	-1.3835487	0.033591732	0.21452866	1	1896	tags=45%, list=14%, signal=53%
REACTOME_CALNEXIN_CALRETICULIN_CYCLE	26	-0.6522686	-1.5192063	0.033816423	0.11467499	1	774	tags=38%, list=6%, signal=41%
REACTOME_DOWNREGULATION_OF_TGF_BETA_RECEPTOR_SIGNALING	26	-0.6219175	-1.4442781	0.035128806	0.16290675	1	690	tags=31%, list=5%, signal=32%
REACTOME_NUCLEAR_IMPORT_OF_REV_PROTEIN	33	-0.5902041	-1.4598415	0.035532996	0.15118088	1	3015	tags=48%, list=22%, signal=62%
REACTOME_DNA_DOUBLE_STRAND_BREAK_RESPONSE	52	-0.5307611	-1.3989482	0.03562341	0.20381168	1	1751	tags=25%, list=13%, signal=29%
PID_MYC_ACTIV_PATHWAY	75	-0.4700304	-1.3338863	0.03626943	0.2554375	1	1690	tags=33%, list=12%, signal=38%
REACTOME_TRANSCRIPTIONAL_REGULATION_BY_RUNX3	93	-0.4480117	-1.3011894	0.036827195	0.28667238	1	1409	tags=30%, list=10%, signal=33%
REACTOME_SNRNP_ASSEMBLY	52	-0.521635	-1.3979479	0.03892944	0.20369233	1	2637	tags=40%, list=19%, signal=50%
REACTOME_PROCESSIVE_SYNTHESIS_ON_THE_LAGGING_STRAND	15	-0.7178278	-1.4763408	0.03908046	0.14228244	1	1708	tags=40%, list=12%, signal=46%
REACTOME_GLOBAL_GENOME_NUCLEOTIDE_EXCISION_REPAIR_GG_NER	84	-0.4700673	-1.3313725	0.04011461	0.25500736	1	2342	tags=38%, list=17%, signal=46%
REACTOME_UNFOLDED_PROTEIN_RESPONSE_UPR	86	-0.4719456	-1.3575081	0.04071247	0.24210317	1	1852	tags=36%, list=13%, signal=41%
REACTOME_PINK1_PRKN_MEDIATED_MITOPHAGY	22	-0.6362678	-1.4356754	0.040816326	0.17108385	1	1196	tags=36%, list=9%, signal=40%
REACTOME_ESTROGEN_DEPENDENT_GENE_EXPRESSION	103	-0.4430836	-1.2927089	0.041009463	0.2970646	1	1440	tags=24%, list=10%, signal=27%
REACTOME_HEDGEHOG_ON_STATE	79	-0.4641874	-1.3198375	0.04278075	0.26559165	1	1407	tags=30%, list=10%, signal=34%
HALLMARK_DNA_REPAIR	149	-0.4210083	-1.2855537	0.045454547	0.29708397	1	1717	tags=28%, list=12%, signal=32%
REACTOME_NUCLEAR_EVENTS_STIMULATED_BY_ALK_SIGNALING_IN_CANCER	18	-0.6681303	-1.4595625	0.048309177	0.15019952	1	1435	tags=50%, list=10%, signal=56%
REACTOME_CYCLIN_A_CDK2_ASSOCIATED_EVENTS_AT_S_PHASE_ENTRY	83	-0.4527416	-1.2895584	0.048780486	0.2980266	1	2151	tags=40%, list=16%, signal=47%
REACTOME_GLYOXYLATE_METABOLISM_AND_GLYCINE_DEGRADATION	26	-0.6336204	-1.4538622	0.051454138	0.15478939	1	1419	tags=19%, list=10%, signal=21%
REACTOME_MITOCHONDRIAL_BIOGENESIS	91	-0.447957	-1.2989393	0.051575933	0.28752643	1	2448	tags=40%, list=18%, signal=48%
KEGG_CYTOKINE_CYTOKINE_RECEPTOR_INTERACTION	179	-0.3978962	-1.2497834	0.052173913	0.33118546	1	1193	tags=9%, list=9%, signal=10%
REACTOME_PERK_REGULATES_GENE_EXPRESSION	29	-0.6082838	-1.4160275	0.053117782	0.18872009	1	1654	tags=38%, list=12%, signal=43%
REACTOME_TGF_BETA_RECEPTOR_SIGNALING_ACTIVATES_SMADS	32	-0.5806195	-1.4078187	0.053658538	0.19534907	1	1057	tags=34%, list=8%, signal=37%
REACTOME_ACTIVATION_OF_THE_PRE_REPLICATIVE_COMPLEX	32	-0.5800198	-1.4144943	0.053932585	0.18778731	1	2005	tags=38%, list=15%, signal=44%
REACTOME_RMTS_METHYLATE_HISTONE_ARGININES	42	-0.5238581	-1.3551991	0.054054055	0.23960596	1	1707	tags=26%, list=12%, signal=30%
REACTOME_N_GLYCAN_TRIMMING_IN_THE_ER_AND_CALNEXIN_CALRETICULIN_CYCLE	35	-0.5712462	-1.3967595	0.054117646	0.20236564	1	774	tags=31%, list=6%, signal=33%
REACTOME_UB_SPECIFIC_PROCESSING_PROTEASES	168	-0.3838679	-1.2047119	0.055016182	0.38130632	1	1412	tags=25%, list=10%, signal=28%
REACTOME_DNA_REPAIR	294	-0.3709993	-1.2122973	0.055555556	0.37409866	1	2371	tags=28%, list=17%, signal=33%
REACTOME_SIGNALING_BY_HEDGEHOG	134	-0.4137477	-1.243353	0.05654762	0.33551833	1	1407	tags=22%, list=10%, signal=24%
REACTOME_EXPORT_OF_VIRAL_RIBONUCLEOPROTEINS_FROM_NUCLEUS	32	-0.5796922	-1.4008108	0.05764411	0.20250276	1	3015	tags=47%, list=22%, signal=60%
REACTOME_SIGNALING_BY_FGFR	68	-0.5018342	-1.3887472	0.057788946	0.20849681	1	1440	tags=25%, list=10%, signal=28%
REACTOME_TP53_REGULATES_TRANSCRIPTION_OF_ADDITIONAL_CELL_CYCLE_GENES_WHOSE_EXACT_ROLE_IN_THE_P53_PATHWAY_REMAIN_UNCERTAIN	21	-0.6664584	-1.4790444	0.058295965	0.14162782	1	2503	tags=52%, list=18%, signal=64%
REACTOME_TRANSCRIPTIONAL_REGULATION_BY_RUNX2	106	-0.4266934	-1.2586538	0.059490085	0.3238245	1	1896	tags=33%, list=14%, signal=38%
REACTOME_HIV_TRANSCRIPTION_ELONGATION	42	-0.5345593	-1.3740691	0.06027397	0.22290812	1	1481	tags=33%, list=11%, signal=37%
REACTOME_FORMATION_OF_RNA_POL_II_ELONGATION_COMPLEX	57	-0.5012296	-1.3496749	0.06027397	0.24425232	1	2262	tags=39%, list=16%, signal=46%
REACTOME_DEGRADATION_OF_BETA_CATENIN_BY_THE_DESTRUCTION_COMPLEX	84	-0.4515068	-1.281806	0.061662197	0.30073512	1	1896	tags=39%, list=14%, signal=45%
REACTOME_DEADENYLATION_DEPENDENT_MRNA_DECAY	55	-0.5134686	-1.3567991	0.065	0.2415668	1	2071	tags=42%, list=15%, signal=49%
REACTOME_MITOTIC_PROMETAPHASE	191	-0.3825622	-1.2039616	0.06521739	0.38136014	1	2073	tags=24%, list=15%, signal=28%
REACTOME_ACTIVATION_OF_ANTERIOR_HOX_GENES_IN_HINDBRAIN_DEVELOPMENT_DURING_EARLY_EMBRYOGENESIS	76	-0.4529551	-1.2857246	0.065989845	0.29824412	1	1440	tags=21%, list=10%, signal=23%
REACTOME_FGFR2_MUTANT_RECEPTOR_ACTIVATION	22	-0.6460241	-1.4490395	0.06728538	0.15808412	1	1440	tags=32%, list=10%, signal=35%
REACTOME_SIGNALING_BY_THE_B_CELL_RECEPTOR_BCR	117	-0.4141647	-1.2422545	0.068249255	0.33604315	1	2126	tags=33%, list=15%, signal=39%
KEGG_ANTIGEN_PROCESSING_AND_PRESENTATION	48	-0.5023792	-1.3212017	0.06987952	0.26463974	1	2084	tags=33%, list=15%, signal=39%
REACTOME_PCP_CE_PATHWAY	85	-0.4371873	-1.2622336	0.07065217	0.32012138	1	2126	tags=38%, list=15%, signal=44%
REACTOME_SARS_COV_1_INFECTION	46	-0.5189384	-1.3281765	0.0719603	0.25759113	1	1694	tags=33%, list=12%, signal=37%
REACTOME_SUMOYLATION_OF_SUMOYLATION_PROTEINS	34	-0.5502672	-1.3493081	0.07281554	0.24329133	1	3015	tags=50%, list=22%, signal=64%
REACTOME_EPIGENETIC_REGULATION_OF_GENE_EXPRESSION	106	-0.4207015	-1.2506042	0.074927956	0.33270302	1	1587	tags=27%, list=12%, signal=31%
REACTOME_SIGNALING_BY_FGFR2_IIIA_TM	18	-0.6462374	-1.4153056	0.075294115	0.18815921	1	1440	tags=39%, list=10%, signal=43%
REACTOME_COPI_DEPENDENT_GOLGI_TO_ER_RETROGRADE_TRAFFIC	88	-0.4457475	-1.2572571	0.07611549	0.3248734	1	1725	tags=23%, list=13%, signal=26%
REACTOME_TRANSCRIPTION_OF_THE_HIV_GENOME	68	-0.4692996	-1.2884359	0.07650273	0.29687518	1	1632	tags=28%, list=12%, signal=32%
REACTOME_MITOPHAGY	29	-0.5821548	-1.367344	0.07746479	0.23234534	1	1635	tags=38%, list=12%, signal=43%
REACTOME_FORMATION_OF_INCISION_COMPLEX_IN_GG_NER	43	-0.5172877	-1.3017733	0.07913669	0.2871423	1	1335	tags=33%, list=10%, signal=36%
REACTOME_SYNTHESIS_OF_ACTIVE_UBIQUITIN_ROLES_OF_E1_AND_E2_ENZYMES	30	-0.5794462	-1.3643509	0.07981221	0.23273304	1	1813	tags=47%, list=13%, signal=54%
REACTOME_DUAL_INCISION_IN_GG_NER	41	-0.5187874	-1.3030059	0.07990315	0.28815123	1	2262	tags=37%, list=16%, signal=44%
BIOCARTA_NDKDYNAMIN_PATHWAY	16	-0.6592829	-1.3818681	0.08061002	0.21560813	1	1202	tags=50%, list=9%, signal=55%
REACTOME_MRNA_CAPPING	29	-0.5729129	-1.3450031	0.08252427	0.2441355	1	2693	tags=55%, list=20%, signal=68%
REACTOME_MRNA_DECAY_BY_3_TO_5_EXORIBONUCLEASE	15	-0.6741684	-1.3802799	0.08352145	0.21673486	1	1994	tags=40%, list=15%, signal=47%
REACTOME_TRANSPORT_OF_MATURE_MRNAS_DERIVED_FROM_INTRONLESS_TRANSCRIPTS	42	-0.5106927	-1.3022403	0.08638743	0.28792855	1	2480	tags=38%, list=18%, signal=46%
REACTOME_POSITIVE_EPIGENETIC_REGULATION_OF_RRNA_EXPRESSION	64	-0.4506977	-1.243407	0.08672087	0.3384548	1	1587	tags=30%, list=12%, signal=33%

Supplemental Table 6: DOWN-REGULATED PATHWAYS: BRQ-GN vs Veh-GN

REACTOME_DNA_DAMAGE_RECOGNITION_IN_GG_NER	38	-0.5323355	-1.3301473	0.08685446	0.25580132	1	1907	tags=37%, list=14%, signal=43%
KEGG_TYPE_I_DIABETES_MELLITUS	26	-0.5832569	-1.3437872	0.087804876	0.2445479	1	423	tags=8%, list=3%, signal=8%
REACTOME_TRANSCRIPTIONAL_REGULATION_BY_SMALL_RNAS	64	-0.4733174	-1.2775855	0.08816121	0.30548462	1	2649	tags=33%, list=19%, signal=40%
REACTOME_PROGRAMMED_CELL_DEATH	194	-0.3629035	-1.1556901	0.090277776	0.4378148	1	1435	tags=24%, list=10%, signal=27%
REACTOME_INTERLEUKIN_1_FAMILY_SIGNALING	127	-0.4034115	-1.2097716	0.09063444	0.37594366	1	1664	tags=30%, list=12%, signal=34%
REACTOME_NUCLEAR_ENVELOPE_NE_REASSEMBLY	67	-0.4659897	-1.2726071	0.09375	0.31002802	1	1945	tags=25%, list=14%, signal=29%
REACTOME_GAP_FILLING_DNA_REPAIR_SYNTHESIS_AND_LIGATION_IN_GG_NER	25	-0.5898333	-1.3410739	0.09462915	0.24584548	1	1704	tags=36%, list=12%, signal=41%
REACTOME_CHROMOSOME_MAINTENANCE	105	-0.4158221	-1.2203923	0.09659091	0.36109814	1	1821	tags=25%, list=13%, signal=28%
REACTOME_TELOMERE_MAINTENANCE	79	-0.4385549	-1.2394739	0.09944751	0.33660114	1	2191	tags=29%, list=16%, signal=34%
KEGG_GRAFT_VERSUS_HOST_DISEASE	23	-0.595586	-1.3508474	0.09977324	0.24352477	1	10	tags=4%, list=0%, signal=4%

Supplemental Table 7	Source	Identifier
**Antibodies**		
BUV563 anti mouse CD48	BD Bioscience	Cat # 741258
BUV395 anti mouse CD11b	BD Biosciences	Cat # 565976
APC anti mouse CD11b	Biolegend	Cat # 101212
PE anti mouse CD11b	eBioscience	Cat # 17-0112-82
BV711 anti mouse CD11b	Biolegend	Cat # 101241
PerCPCy5.5 anti mouse CD11b	BioLegend	Cat # 101228
FITC anti mouse Gr-1	Biolegend	Cat # 108405
BV785 anti mouse CD4	Biolegend	Cat # 100453
BV785 anti mouse CD4	BD Bioscience	Cat # 563727
BUV395 anti mouse CD8a	BD Horizon	Cat # 563786
PE anti mouse CD8a	Biolegend	Cat # 100708
AlexaFluor 647 anti mouse CD101	BD Bioscience	Cat # 307707
AlexaFluor 647 IgG2a Isotype Control	BD Bioscience	Cat # 558053
FITC anti mouse Ly6C	BD Bioscience	Cat # 553104
BV421 anti mouse Ly6C	Biolegend	Cat # 128031
BV785 anti mouse Ly6C	BioLegend	Cat # 128041
BV650 anti mouse Ly6C	BioLegend	Cat # 128049
BV570 anti mouse Ly6G	BioLegend	Cat # 127629
BV605 anti mouse Ly6G	BioLegend	Cat # 127639
PacBlue anti mouse Ly6G	Biolegend	Cat # 127612
PE anti mouse Ly6G	BD Bioscience	Cat # 551461
anti CD16/CD32 FC	BD Bioscience	Cat # 553142
PE anti mouse CD45	Biolegend	Cat # 103106
APC anti mouse PD-1	BioLegend	Cat # 135209
BV421 anti mouse Ki-67	BioLegend	Cat # 652411
BV605 anti mouse CD25	BioLegend	Cat # 102036
Ax700 anti mouse CD44	Biolegend	Cat # 103026
PeCy7 anti mouse ICOS	BioLegend	Cat # 313520
BV421 anti mouse F4/80	BioLegend	Cat # 123137
Biotin anti mouse CD81	BioLegend	Cat # 104903
BUV661 Streptavidin	BD Bioscience	Cat # 612979
BUV737 anti mouse CD11c	BD Bioscience	Cat # 612797
BUV805 anti mouse CXCR4	BD Bioscience	Cat # 741979
BV421 anti mouse CX3CR1	BioLegend	Cat # 149023
eFluor450 anti mouse CD105	ThermoFisher	Cat # 48-1057-42
BV480 anti mouse CD106	BD Bioscience	Cat # 746326
BV510 anti mouse Siglec F	BD Bioscience	Cat # 740158
BV605 anti mouse CD115	BioLegend	Cat # 135517
BV711 anti mouse CD16/32	BioLegend	Cat # 101337
BV785 anti mouse CD86	BioLegend	Cat # 105043
AlexaFluor 488 anti mouse PD-1	ThermoFisher	Cat # 53-9969-42
PE anti mouse VEGF	Novus	Cat # NB100-664PE
AlexaFluor 594 anti mouse iNOS	BioLegend	Cat # 696804
PE anti mouse CD101	ThermoFisher	Cat # 12-1011-82
PE-Dazzle 594 anti mouse Sca-1	BioLegend	Cat # 122527
PE-Cy5 anti mouse Flt3	BioLegend	Cat # 135311
PerCp-Cy5.5 anti mouse MHCII	BioLegend	Cat # 107625
PerCp-eFluor710 anti mouse CD41	ThermoFisher	Cat # 46-0411-82
PE-Cy7 anti mouse CD24	BioLegend	Cat # 101821
APC anti mouse CD172a	BioLegend	Cat # 144013
AlexaFluor 647 anti mouse CD150	BioLegend	Cat # 115918
AlexaFluor 700 anti mouse CD3	BioLegend	Cat # 300423
AlexaFluor 700 anti mouse B220	BioLegend	Cat # 103231
AlexaFluor 700 anti mouse NK1.1	BioLegend	Cat # 108729
AlexaFluor 700 anti mouse Ter119	BioLegend	Cat # 116220
APC-Cy7 anti mouse c-Kit	BioLegend	Cat # 105825
PE/Cy7 anti-mouse PD-L1	BioLegend	Cat # 124314
PE anti-mouse PD-L2	BioLegend	Cat # 107205
InVivoMAb anti mouse PD-1	BioXCell	Cat # BE0146
InVivoMAb rat IgG2a Isotype	BioXCell	Cat # BE0089
InVivoMAb anti mouse CD8a	BioXCell	Cat # BE0061
InVivoMAb rat IgG2b Isotype	BioXCell	Cat # BE0090
InVivoPlus anti mouse CTLA-4	BioXCell	Cat # BP0131
Syrian Hamster IgG Isotype	Leinco	Cat # I-444
Rat IgG2b Isotype	Leinco	Cat # R1371
Ly-6G(Gr-1) monoclonal AB	Invitrogen	Cat # 14-5931-82
CD45R monoclonal AB	Invitrogen	Cat # 14-0452-82
CD3 Monoclonal AB (17A2)	Invitrogen	Cat # 14-0032-82
TER-119 monoclonal AB	Invitrogen	Cat # 14-5921-82
CD11b monoclonal AB	Invitrogen	Cat # 14-0112-82
APC anti human CD101	Biolegend	Cat # 331007
PE anti human CD11b	BD Bioscience	Cat # 557321
APC anti human HLA-DR	Biolegend	Cat # 307610
PE/Cy7 anti human CD33	Biolegend	Cat # 366617

NA/LE mouse CD3e	BD Bioscience	Cat # 553057
**Biological Samples**		
Human bone marrow from healthy donors	Roswell Park Comprehensive Cancer Center	BDR 134520
**Chemicals, Peptides, and Recombinant Proteins**		
Mouse G-CSF	Peprotech	Cat # 250-05
Mouse GM-CSF	Peprotech	Cat # 315-03
Human G-CSF	Peprotech	Cat # 300-23
Human GM-CSF	Peprotech	Cat # 300-03
CellTrace Violet (CTV) Dye	Invitrogen	Cat # C34557
Dulbecco's PBS	Corning	Cat # 20-031-CV
FBS	Corning	Cat # 35-011-CV
EdTA	Corning	Cat # 46-034-CL
Trypsin EDTA 1X	Corning	Cat # 25-053-CL
Trypan Blue Solution, 0.4%	Gibco	Cat # 15250061
MACS Buffer	Miltenyi	Cat # 130-091-221
Lenflunomide	Krackeler Scientific	Cat # L5025-25mg
Brequinar Sodium	Tocris	Cat # 6196
Brequinar Sodium	Clear Creek Bio	N/A
Uridine $\geq$ 99%	Sigma-Aldrich	Cat # U3750
Hematrue Diluent Solution	Heska	Cat # 5613
Hematrue Enzymatic Cleaner	Heska	Cat # 5616
Hematrue Lysing Reagent	Heska	Cat # 5615
FITC Annexin V	BioLegend	Cat # 640906
10x Annexin V Binding Buffer	eBioscience	Cat # 00-0055-43
eBioscience Foxp3 Fixation/Permeabilization Kit	eBioscience	Cat # 00-5521-00
DAPI	Thermofisher	Cat # 62248
ACK Lysis Buffer	Gibco	Cat # A1049201
Matrigel	Corning Inc.	Cat # 356234
L-glutamine	Gibco	Cat # 25030081
Sodium Pyruvate	Gibco	Cat # 11360070
2-mercaptoethanol	Gibco	Cat # 31350010
Collagenase/ Hyaluronidase	Stemcell	Cat # 07912
LD Aqua	Invitrogen	Cat # L34957
Live/Dead Blue	ThermoFisher	Cat # L23105
BioMag Goat Anti-Rat IgG Beads	Qiagen	Cat # 310107
SYBR Green PCR Master Mix	Applied Biosystems	Cat # 4309155
**Critical Commercial Assays**		
Rneasy Mini Kit	Qiagen	Cat # 74104
MDSC Isolation Kit, mouse	Miltenyi	Cat # 130-094-538
EasySep Mouse MDSC Isolation Kit	StemCell	Cat # 19867
iScript cDNA synthesis kit	Bio-Rad	Cat # 1708890
Micro BCA Protein Assay kit	ThermoFisher	Cat # 23235
Arginase Activity Assay kit	Sigma-Aldrich	Cat # MAK112
**Depositied Data**		
RNA-Seq Data	This paper	GEO: GSE190232
	Netherby et al., 2017	GEO: GSE193263
**Experimental Models: Cell Lines**		
4T1	ATCC	Cat # CRL-2539
E0771.ML-1	Liu et al., 2021 (gift from Cornell Univ.)	
**Experimental Models: Organisms/ Strains**		
Irf8 $^{-/-}$ Mice (On both C57BL/6 and BALB/c backgrounds)	Ozato et al., 2006, Waight et al., 2013	
C57BL/6 Mice	Charles River Laboratories	Strain Code 556
BALB/c Mice	Charles River Laboratories	Strain Code 555
**Oligonucleotides**		
ARG1 Primer	Forward (5'-->3') TTTTTCCAGCAGACCAGCTT	Reverse (5'-->3') AGAGATTATCGGAGCGCCTT
NOS2 Primer	Forward (5'-->3') GTCGATGTCACATGCAGCTT	Reverse (5'-->3') GAAGAAAACCCCTTGTGCTG
VEGF Primer	Forward (5'-->3') CTGCTGTAACGATGAAGCCCTG	Reverse (5'-->3') GCTGTAGGAAGCTCATCTCTCC
TGF-beta Primer	Forward (5'-->3') GTGGAAATCAACGGGATCAG	Reverse (5'-->3') ACTTCCAACCCAGGTCCTTC
CD84 Primer	Forward (5'-->3') ATATAGCTGGAGTCCCTTTGGAG	Reverse (5'-->3') AAAGAGCACGGCCAATCCTC
JAML Primer	Forward (5'-->3') ATGCTTTGCCTCCTGAAACTG	Reverse (5'-->3') TGATTCACCCACATGCACTCT
PD-L1 Primer	Forward (5'-->3') TGCGGACTACAAGCGAATCACG	Reverse (5'-->3') CTCAGCTTCTGGATAACCCTCG
PD-L2 Primer	Forward (5'-->3'): CTGGGACTACAAGTACCTGACG	Reverse (5'-->3'): CTCTAGCCTGGCAGGTAAGCTG
S100A8	Forward (5'-->3'): TGCCACACCCACTTTTATCA	Reverse (5'-->3'): GAGTGTCCTCAGTTTGTGCAG
S100A9	Forward (5'-->3'): AGATGGCCAACAAAGCACCT	Reverse (5'-->3'): TAAAGGTTGCCAACTGTGCT
IRF8 Primer	Forward (5'->3') TGGAAGCATCCACCTCCTGATTGT	Reverse (5'->3') TGATCGAACAGATCGACAGCAGCA
DNAJB11 Primer	Forward (5'-->3'): CGCAGAACCTGAGCACCTTC	Reverse (5'-->3'): CAGTCCCGATGAGGTACAGCA
CHOP Primer	Forward (5'-->3'): CTGCCTTTCACCTTGGAGAC	Reverse (5'-->3'): CGTTTCCTGGGGATGAGATA
CDC42 Primer	Forward (5'-->3'): GTTGGTGATGGTGCTGTT	Reverse (5'-->3'): GGATAACTTAGCGGTCGT
PPIA Primer	Forward (5'-->3'): GGCAAATGCTGGACCAAAC	Reverse (5'-->3'): CATTCCTGGACCCAAAACG

Taqman probe for human iNOS	Thermofisher	Hs01075529_m1
Taqman probe for human Arg-1	Thermofisher	Hs00163660_m1
Taqman probe for human IL-10	Thermofisher	Hs00961622_m1
**Other**		
Hard-Shell PCR Plates 96 Well	Bio-Rad	Cat # HSP9601
75 cm Cell Culture Flask Vented	Thermofisher	Cat # 130190
Biolite 96 Well Multidish	Thermofisher	Cat # 130188
Cell Strainers	Corning	
Cell Strainers	MTC Bio	Cat # C4100
U-Bottom 96 Well Plate	Thermofisher	Cat # 163320
5 mL Polystyrene Rount-Bottom Tube	Falcon	Cat # 352052
LS MACS Separation Columns	Miltenyi	Cat # 130-042-401
QuadroMACS Separator	Miltenyi	Cat # 130-090-976
GentleMACS C Tubes	Miltenyi	Cat # 130-096-334
Non Treated Suspension Culture Dish	Corning	Cat # 430591
Microvette 100 EDTA	Sarstedt	Cat # 20-1278-100

Supplemental Tables 1-6, Related to Figure 7. Complete lists of pathways upregulated in Veh-GMPs versus NTB-GMPs (Table 1**)**; downregulated in Veh-GMPs versus NTB-GMPs (Table 2); upregulated in BRQ-GMPs versus Veh-GMPs (Table 3); downregulated in BRQ-GMPs versus Veh-GMPs (Table 4); upregulated in BRQ-GNs versus Veh-GNs (Table 5**)**; downregulated in BRQ-GNs versus Veh-GNs (Table 6). All pathways shown in tables were identified using Gene Set Enrichment Analysis with nominal (NOM) p-values < 0.01 and false discovery rate (FDR) q-values < 0.25. Supplemental Table 7 is a complete list of the key resources used in this study.
